# Supplementary material for: Genome-wide identification, characterization and gene expression of BES1 transcription factor family in grapevine (Vitis vinifera L.)
Source: Sci Rep. 2023 Jan 5;13:240. doi: 10.1038/s41598-022-24407-y (PMC9816167; doi:10.1038/s41598-022-24407-y)
Supplement: Supplementary file 3 — Supplementary Information. [file 41598_2022_24407_MOESM3_ESM.zip › Vvi_Vvi/Vitis_vinifera.PN40024.v4.dna_sm.toplevel.fa.vs.Vitis_vinifera.PN40024.v4.dna_sm.toplevel.fa.html/10.html]

|  |  |  |  |  |  |  |  |  |  |  |  |  |  |
| --- | --- | --- | --- | --- | --- | --- | --- | --- | --- | --- | --- | --- | --- |
| Duplication depth | Reference chromosome | Collinear blocks | | | | | | | | | | | |
| 0 | Vitvi10g04000\_t001 |  |  |  |  |  |  |  |  |  |  |  |  |
| 0 | Vitvi10g04001\_t001 |  |  |  |  |  |  |  |  |  |  |  |  |
| 0 | Vitvi10g04002\_t001 |  |  |  |  |  |  |  |  |  |  |  |  |
| 0 | Vitvi10g00002\_t001 |  |  |  |  |  |  |  |  |  |  |  |  |
| 0 | Vitvi10g00003\_t001 |  |  |  |  |  |  |  |  |  |  |  |  |
| 1 | Vitvi10g04003\_t001 |  | Vitvi12g00546\_t001 |  |  |  |  |  |  |  |  |  |  |
| 1 | Vitvi10g04004\_t001 |  | \| \| |  |  |  |  |  |  |  |  |  |  |
| 1 | Vitvi10g00007\_t001 |  | \| \| |  |  |  |  |  |  |  |  |  |  |
| 1 | Vitvi10g00008\_t001 |  | \| \| |  |  |  |  |  |  |  |  |  |  |
| 1 | Vitvi10g00011\_t001 |  | \| \| |  |  |  |  |  |  |  |  |  |  |
| 1 | Vitvi10g01605\_t001 |  | \| \| |  |  |  |  |  |  |  |  |  |  |
| 1 | Vitvi10g00012\_t001 |  | \| \| |  |  |  |  |  |  |  |  |  |  |
| 1 | Vitvi10g04005\_t001 |  | \| \| |  |  |  |  |  |  |  |  |  |  |
| 1 | Vitvi10g04006\_t001 |  | \| \| |  |  |  |  |  |  |  |  |  |  |
| 1 | Vitvi10g00015\_t001 |  | \| \| |  |  |  |  |  |  |  |  |  |  |
| 1 | Vitvi10g04007\_t001 |  | \| \| |  |  |  |  |  |  |  |  |  |  |
| 1 | Vitvi10g00016\_t001 |  | \| \| |  |  |  |  |  |  |  |  |  |  |
| 1 | Vitvi10g00017\_t001 |  | Vitvi12g00532\_t001 |  |  |  |  |  |  |  |  |  |  |
| 1 | Vitvi10g01606\_t001 |  | \| \| |  |  |  |  |  |  |  |  |  |  |
| 1 | Vitvi10g04008\_t001 |  | \| \| |  |  |  |  |  |  |  |  |  |  |
| 1 | Vitvi10g00019\_t001 |  | \| \| |  |  |  |  |  |  |  |  |  |  |
| 2 | Vitvi10g00020\_t001 |  | \| \| |  | Vitvi19g00431\_t001 |  |  |  |  |  |  |  |  |
| 2 | Vitvi10g00022\_t001 |  | \| \| |  | \| \| |  |  |  |  |  |  |  |  |
| 2 | Vitvi10g00023\_t002 |  | \| \| |  | \| \| |  |  |  |  |  |  |  |  |
| 2 | Vitvi10g00024\_t001 |  | Vitvi12g00531\_t001 |  | \| \| |  |  |  |  |  |  |  |  |
| 2 | Vitvi10g01607\_t001 |  | \| \| |  | \| \| |  |  |  |  |  |  |  |  |
| 2 | Vitvi10g01608\_t001 |  | Vitvi12g04158\_t002 |  | \| \| |  |  |  |  |  |  |  |  |
| 2 | Vitvi10g00025\_t001 |  | \| \| |  | \| \| |  |  |  |  |  |  |  |  |
| 2 | Vitvi10g00026\_t001 |  | \| \| |  | \| \| |  |  |  |  |  |  |  |  |
| 2 | Vitvi10g00027\_t001 |  | \| \| |  | Vitvi19g00441\_t001 |  |  |  |  |  |  |  |  |
| 2 | Vitvi10g00028\_t001 |  | \| \| |  | \| \| |  |  |  |  |  |  |  |  |
| 2 | Vitvi10g00029\_t001 |  | \| \| |  | \| \| |  |  |  |  |  |  |  |  |
| 2 | Vitvi10g00030\_t001 |  | \| \| |  | \| \| |  |  |  |  |  |  |  |  |
| 2 | Vitvi10g00031\_t001 |  | \| \| |  | \| \| |  |  |  |  |  |  |  |  |
| 2 | Vitvi10g01609\_t001 |  | \| \| |  | \| \| |  |  |  |  |  |  |  |  |
| 2 | Vitvi10g00032\_t001 |  | \| \| |  | Vitvi19g00442\_t001 |  |  |  |  |  |  |  |  |
| 2 | Vitvi10g00033\_t001 |  | Vitvi12g00522\_t001 |  | Vitvi19g00449\_t001 |  |  |  |  |  |  |  |  |
| 2 | Vitvi10g00034\_t001 |  | \| \| |  | \| \| |  |  |  |  |  |  |  |  |
| 2 | Vitvi10g04009\_t001 |  | \| \| |  | \| \| |  |  |  |  |  |  |  |  |
| 2 | Vitvi10g00035\_t001 |  | \| \| |  | \| \| |  |  |  |  |  |  |  |  |
| 2 | Vitvi10g01611\_t001 |  | \| \| |  | Vitvi19g04225\_t001 |  |  |  |  |  |  |  |  |
| 2 | Vitvi10g00037\_t001 |  | \| \| |  | Vitvi19g00457\_t001 |  |  |  |  |  |  |  |  |
| 2 | Vitvi10g00039\_t001 |  | \| \| |  | \| \| |  |  |  |  |  |  |  |  |
| 2 | Vitvi10g00040\_t001 |  | \| \| |  | \| \| |  |  |  |  |  |  |  |  |
| 2 | Vitvi10g00041\_t001 |  | \| \| |  | \| \| |  |  |  |  |  |  |  |  |
| 2 | Vitvi10g00043\_t001 |  | Vitvi12g00516\_t001 |  | \| \| |  |  |  |  |  |  |  |  |
| 1 | Vitvi10g00044\_t001 |  |  |  | \| \| |  |  |  |  |  |  |  |  |
| 1 | Vitvi10g00045\_t001 |  |  |  | \| \| |  |  |  |  |  |  |  |  |
| 1 | Vitvi10g01612\_t001 |  |  |  | Vitvi19g04226\_t001 |  |  |  |  |  |  |  |  |
| 1 | Vitvi10g00046\_t001 |  |  |  | Vitvi19g00470\_t001 |  |  |  |  |  |  |  |  |
| 0 | Vitvi10g00047\_t001 |  |  |  |  |  |  |  |  |  |  |  |  |
| 0 | Vitvi10g00048\_t002 |  |  |  |  |  |  |  |  |  |  |  |  |
| 0 | Vitvi10g00049\_t001 |  |  |  |  |  |  |  |  |  |  |  |  |
| 1 | Vitvi10g00050\_t001 |  | Vitvi12g00415\_t001 |  |  |  |  |  |  |  |  |  |  |
| 1 | Vitvi10g01613\_t001 |  | \| \| |  |  |  |  |  |  |  |  |  |  |
| 1 | Vitvi10g00051\_t001 |  | \| \| |  |  |  |  |  |  |  |  |  |  |
| 1 | Vitvi10g00052\_t004 |  | Vitvi12g00410\_t001 |  |  |  |  |  |  |  |  |  |  |
| 1 | Vitvi10g00053\_t001 |  | \| \| |  |  |  |  |  |  |  |  |  |  |
| 1 | Vitvi10g00054\_t001 |  | \| \| |  |  |  |  |  |  |  |  |  |  |
| 1 | Vitvi10g00055\_t001 |  | \| \| |  |  |  |  |  |  |  |  |  |  |
| 1 | Vitvi10g04010\_t001 |  | Vitvi12g00404\_t001 |  |  |  |  |  |  |  |  |  |  |
| 1 | Vitvi10g00056\_t001 |  | \| \| |  |  |  |  |  |  |  |  |  |  |
| 2 | Vitvi10g00057\_t001 |  | \| \| |  | Vitvi19g00537\_t002 |  |  |  |  |  |  |  |  |
| 2 | Vitvi10g01614\_t001 |  | \| \| |  | Vitvi19g00536\_t001 |  |  |  |  |  |  |  |  |
| 2 | Vitvi10g01615\_t001 |  | \| \| |  | \| \| |  |  |  |  |  |  |  |  |
| 2 | Vitvi10g01616\_t001 |  | \| \| |  | Vitvi19g02031\_t001 |  |  |  |  |  |  |  |  |
| 2 | Vitvi10g01617\_t001 |  | \| \| |  | \| \| |  |  |  |  |  |  |  |  |
| 2 | Vitvi10g01618\_t001 |  | \| \| |  | \| \| |  |  |  |  |  |  |  |  |
| 2 | Vitvi10g01619\_t001 |  | \| \| |  | Vitvi19g00534\_t001 |  |  |  |  |  |  |  |  |
| 2 | Vitvi10g01620\_t001 |  | \| \| |  | \| \| |  |  |  |  |  |  |  |  |
| 2 | Vitvi10g00060\_t001 |  | \| \| |  | \| \| |  |  |  |  |  |  |  |  |
| 2 | Vitvi10g00061\_t001 |  | \| \| |  | \| \| |  |  |  |  |  |  |  |  |
| 2 | Vitvi10g00062\_t001 |  | \| \| |  | \| \| |  |  |  |  |  |  |  |  |
| 2 | Vitvi10g00063\_t001 |  | Vitvi12g00388\_t001 |  | Vitvi19g00530\_t001 |  |  |  |  |  |  |  |  |
| 2 | Vitvi10g00064\_t001 |  | \| \| |  | Vitvi19g00529\_t001 |  |  |  |  |  |  |  |  |
| 2 | Vitvi10g01621\_t001 |  | Vitvi12g00387\_t001 |  | Vitvi19g00528\_t001 |  |  |  |  |  |  |  |  |
| 2 | Vitvi10g00065\_t001 |  | Vitvi12g00386\_t001 |  | \| \| |  |  |  |  |  |  |  |  |
| 2 | Vitvi10g04011\_t001 |  | Vitvi12g00385\_t001 |  | \| \| |  |  |  |  |  |  |  |  |
| 2 | Vitvi10g00068\_t001 |  | \| \| |  | \| \| |  |  |  |  |  |  |  |  |
| 2 | Vitvi10g00069\_t001 |  | \| \| |  | \| \| |  |  |  |  |  |  |  |  |
| 2 | Vitvi10g04012\_t001 |  | \| \| |  | \| \| |  |  |  |  |  |  |  |  |
| 2 | Vitvi10g04013\_t001 |  | \| \| |  | \| \| |  |  |  |  |  |  |  |  |
| 2 | Vitvi10g04014\_t001 |  | \| \| |  | \| \| |  |  |  |  |  |  |  |  |
| 2 | Vitvi10g04015\_t001 |  | \| \| |  | \| \| |  |  |  |  |  |  |  |  |
| 2 | Vitvi10g04016\_t001 |  | \| \| |  | \| \| |  |  |  |  |  |  |  |  |
| 2 | Vitvi10g00070\_t002 |  | \| \| |  | \| \| |  |  |  |  |  |  |  |  |
| 2 | Vitvi10g04017\_t001 |  | \| \| |  | \| \| |  |  |  |  |  |  |  |  |
| 2 | Vitvi10g00073\_t002 |  | \| \| |  | Vitvi19g00521\_t001 |  |  |  |  |  |  |  |  |
| 2 | Vitvi10g04018\_t001 |  | Vitvi12g00384\_t001 |  | \| \| |  |  |  |  |  |  |  |  |
| 2 | Vitvi10g00076\_t001 |  | \| \| |  | \| \| |  |  |  |  |  |  |  |  |
| 2 | Vitvi10g00077\_t001 |  | \| \| |  | \| \| |  |  |  |  |  |  |  |  |
| 2 | Vitvi10g00078\_t001.1.6037826a |  | \| \| |  | \| \| |  |  |  |  |  |  |  |  |
| 2 | Vitvi10g04019\_t001 |  | \| \| |  | \| \| |  |  |  |  |  |  |  |  |
| 2 | Vitvi10g00079\_t001 |  | \| \| |  | \| \| |  |  |  |  |  |  |  |  |
| 2 | Vitvi10g00080\_t001 |  | \| \| |  | \| \| |  |  |  |  |  |  |  |  |
| 2 | Vitvi10g04020\_t001 |  | \| \| |  | \| \| |  |  |  |  |  |  |  |  |
| 2 | Vitvi10g04021\_t001 |  | \| \| |  | \| \| |  |  |  |  |  |  |  |  |
| 2 | Vitvi10g04022\_t001 |  | \| \| |  | \| \| |  |  |  |  |  |  |  |  |
| 2 | Vitvi10g00082\_t001 |  | Vitvi12g00382\_t001 |  | \| \| |  |  |  |  |  |  |  |  |
| 2 | Vitvi10g00083\_t001 |  | \| \| |  | \| \| |  |  |  |  |  |  |  |  |
| 2 | Vitvi10g00084\_t001 |  | \| \| |  | \| \| |  |  |  |  |  |  |  |  |
| 2 | Vitvi10g00085\_t001 |  | \| \| |  | \| \| |  |  |  |  |  |  |  |  |
| 2 | Vitvi10g00086\_t001 |  | Vitvi12g00381\_t001 |  | \| \| |  |  |  |  |  |  |  |  |
| 2 | Vitvi10g04023\_t001 |  | \| \| |  | \| \| |  |  |  |  |  |  |  |  |
| 2 | Vitvi10g00087\_t001 |  | \| \| |  | Vitvi19g02023\_t001 |  |  |  |  |  |  |  |  |
| 2 | Vitvi10g00088\_t001 |  | Vitvi12g00380\_t001 |  | \| \| |  |  |  |  |  |  |  |  |
| 2 | Vitvi10g00090\_t001 |  | \| \| |  | \| \| |  |  |  |  |  |  |  |  |
| 2 | Vitvi10g00091\_t001 |  | Vitvi12g04114\_t001 |  | \| \| |  |  |  |  |  |  |  |  |
| 2 | Vitvi10g04024\_t001 |  | Vitvi12g00370\_t002 |  | Vitvi19g02021\_t001 |  |  |  |  |  |  |  |  |
| 2 | Vitvi10g00092\_t001 |  | Vitvi12g00369\_t001 |  | Vitvi19g02020\_t001 |  |  |  |  |  |  |  |  |
| 1 | Vitvi10g00093\_t001 |  |  |  | Vitvi19g04237\_t001 |  |  |  |  |  |  |  |  |
| 1 | Vitvi10g00094\_t001 |  |  |  | \| \| |  |  |  |  |  |  |  |  |
| 1 | Vitvi10g00095\_t001 |  |  |  | Vitvi19g00504\_t001 |  |  |  |  |  |  |  |  |
| 1 | Vitvi10g00096\_t001 |  |  |  | \| \| |  |  |  |  |  |  |  |  |
| 1 | Vitvi10g04025\_t001 |  |  |  | \| \| |  |  |  |  |  |  |  |  |
| 1 | Vitvi10g01641\_t001 |  |  |  | \| \| |  |  |  |  |  |  |  |  |
| 1 | Vitvi10g00098\_t001 |  |  |  | \| \| |  |  |  |  |  |  |  |  |
| 1 | Vitvi10g00100\_t001 |  |  |  | \| \| |  |  |  |  |  |  |  |  |
| 1 | Vitvi10g00101\_t001 |  |  |  | \| \| |  |  |  |  |  |  |  |  |
| 1 | Vitvi10g00102\_t001 |  |  |  | Vitvi19g00497\_t001 |  |  |  |  |  |  |  |  |
| 1 | Vitvi10g01642\_t001 |  |  |  | \| \| |  |  |  |  |  |  |  |  |
| 1 | Vitvi10g00105\_t001 |  |  |  | Vitvi19g00492\_t001 |  |  |  |  |  |  |  |  |
| 1 | Vitvi10g01643\_t001 |  |  |  | Vitvi19g02012\_t001 |  |  |  |  |  |  |  |  |
| 0 | Vitvi10g00106\_t001 |  |  |  |  |  |  |  |  |  |  |  |  |
| 0 | Vitvi10g00107\_t001 |  |  |  |  |  |  |  |  |  |  |  |  |
| 0 | Vitvi10g00109\_t001 |  |  |  |  |  |  |  |  |  |  |  |  |
| 0 | Vitvi10g01644\_t001 |  |  |  |  |  |  |  |  |  |  |  |  |
| 0 | Vitvi10g00110\_t001 |  |  |  |  |  |  |  |  |  |  |  |  |
| 0 | Vitvi10g00111\_t001 |  |  |  |  |  |  |  |  |  |  |  |  |
| 0 | Vitvi10g04026\_t001 |  |  |  |  |  |  |  |  |  |  |  |  |
| 0 | Vitvi10g04027\_t001 |  |  |  |  |  |  |  |  |  |  |  |  |
| 0 | Vitvi10g04028\_t001 |  |  |  |  |  |  |  |  |  |  |  |  |
| 0 | Vitvi10g04029\_t001 |  |  |  |  |  |  |  |  |  |  |  |  |
| 0 | Vitvi10g02102\_t001 |  |  |  |  |  |  |  |  |  |  |  |  |
| 0 | Vitvi10g02101\_t001 |  |  |  |  |  |  |  |  |  |  |  |  |
| 0 | Vitvi10g04030\_t001 |  |  |  |  |  |  |  |  |  |  |  |  |
| 0 | Vitvi10g04031\_t001 |  |  |  |  |  |  |  |  |  |  |  |  |
| 0 | Vitvi10g02099\_t001 |  |  |  |  |  |  |  |  |  |  |  |  |
| 0 | Vitvi10g02097\_t001 |  |  |  |  |  |  |  |  |  |  |  |  |
| 0 | Vitvi10g04032\_t001 |  |  |  |  |  |  |  |  |  |  |  |  |
| 0 | Vitvi10g04033\_t001 |  |  |  |  |  |  |  |  |  |  |  |  |
| 0 | Vitvi10g04034\_t004 |  |  |  |  |  |  |  |  |  |  |  |  |
| 0 | Vitvi10g02089\_t001 |  |  |  |  |  |  |  |  |  |  |  |  |
| 0 | Vitvi10g04035\_t001 |  |  |  |  |  |  |  |  |  |  |  |  |
| 0 | Vitvi10g04036\_t001 |  |  |  |  |  |  |  |  |  |  |  |  |
| 0 | Vitvi10g04037\_t001 |  |  |  |  |  |  |  |  |  |  |  |  |
| 0 | Vitvi10g00130\_t001 |  |  |  |  |  |  |  |  |  |  |  |  |
| 0 | Vitvi10g00129\_t001 |  |  |  |  |  |  |  |  |  |  |  |  |
| 0 | Vitvi10g00128\_t001 |  |  |  |  |  |  |  |  |  |  |  |  |
| 0 | Vitvi10g00127\_t001 |  |  |  |  |  |  |  |  |  |  |  |  |
| 0 | Vitvi10g01647\_t001 |  |  |  |  |  |  |  |  |  |  |  |  |
| 0 | Vitvi10g00125\_t001 |  |  |  |  |  |  |  |  |  |  |  |  |
| 0 | Vitvi10g00124\_t001 |  |  |  |  |  |  |  |  |  |  |  |  |
| 0 | Vitvi10g00123\_t002 |  |  |  |  |  |  |  |  |  |  |  |  |
| 0 | Vitvi10g00122\_t001 |  |  |  |  |  |  |  |  |  |  |  |  |
| 0 | Vitvi10g00121\_t001 |  |  |  |  |  |  |  |  |  |  |  |  |
| 0 | Vitvi10g00120\_t001 |  |  |  |  |  |  |  |  |  |  |  |  |
| 0 | Vitvi10g04038\_t001 |  |  |  |  |  |  |  |  |  |  |  |  |
| 0 | Vitvi10g00119\_t002 |  |  |  |  |  |  |  |  |  |  |  |  |
| 0 | Vitvi10g00117\_t001 |  |  |  |  |  |  |  |  |  |  |  |  |
| 0 | Vitvi10g00116\_t001 |  |  |  |  |  |  |  |  |  |  |  |  |
| 0 | Vitvi10g04039\_t001 |  |  |  |  |  |  |  |  |  |  |  |  |
| 0 | Vitvi10g01650\_t001 |  |  |  |  |  |  |  |  |  |  |  |  |
| 0 | Vitvi10g00131\_t001 |  |  |  |  |  |  |  |  |  |  |  |  |
| 0 | Vitvi10g00134\_t001 |  |  |  |  |  |  |  |  |  |  |  |  |
| 0 | Vitvi10g04040\_t001 |  |  |  |  |  |  |  |  |  |  |  |  |
| 0 | Vitvi10g04041\_t001 |  |  |  |  |  |  |  |  |  |  |  |  |
| 0 | Vitvi10g04042\_t001 |  |  |  |  |  |  |  |  |  |  |  |  |
| 0 | Vitvi10g04043\_t001 |  |  |  |  |  |  |  |  |  |  |  |  |
| 0 | Vitvi10g04044\_t001 |  |  |  |  |  |  |  |  |  |  |  |  |
| 0 | Vitvi10g01662\_t001 |  |  |  |  |  |  |  |  |  |  |  |  |
| 0 | Vitvi10g04045\_t001 |  |  |  |  |  |  |  |  |  |  |  |  |
| 0 | Vitvi10g04046\_t001 |  |  |  |  |  |  |  |  |  |  |  |  |
| 0 | Vitvi10g01655\_t001 |  |  |  |  |  |  |  |  |  |  |  |  |
| 0 | Vitvi10g00154\_t001 |  |  |  |  |  |  |  |  |  |  |  |  |
| 0 | Vitvi10g00136\_t001 |  |  |  |  |  |  |  |  |  |  |  |  |
| 0 | Vitvi10g00138\_t001 |  |  |  |  |  |  |  |  |  |  |  |  |
| 0 | Vitvi10g04047\_t001 |  |  |  |  |  |  |  |  |  |  |  |  |
| 0 | Vitvi10g04048\_t001 |  |  |  |  |  |  |  |  |  |  |  |  |
| 0 | Vitvi10g04049\_t001 |  |  |  |  |  |  |  |  |  |  |  |  |
| 0 | Vitvi10g04050\_t001 |  |  |  |  |  |  |  |  |  |  |  |  |
| 0 | Vitvi10g04051\_t001 |  |  |  |  |  |  |  |  |  |  |  |  |
| 0 | Vitvi10g04052\_t001 |  |  |  |  |  |  |  |  |  |  |  |  |
| 0 | Vitvi10g04053\_t001 |  |  |  |  |  |  |  |  |  |  |  |  |
| 0 | Vitvi10g04054\_t001 |  |  |  |  |  |  |  |  |  |  |  |  |
| 0 | Vitvi10g04055\_t001 |  |  |  |  |  |  |  |  |  |  |  |  |
| 0 | Vitvi10g04056\_t001 |  |  |  |  |  |  |  |  |  |  |  |  |
| 0 | Vitvi10g04057\_t001 |  |  |  |  |  |  |  |  |  |  |  |  |
| 0 | Vitvi10g04058\_t001 |  |  |  |  |  |  |  |  |  |  |  |  |
| 0 | Vitvi10g04059\_t001 |  |  |  |  |  |  |  |  |  |  |  |  |
| 0 | Vitvi10g04060\_t001 |  |  |  |  |  |  |  |  |  |  |  |  |
| 0 | Vitvi10g01657\_t001 |  |  |  |  |  |  |  |  |  |  |  |  |
| 0 | Vitvi10g04061\_t001 |  |  |  |  |  |  |  |  |  |  |  |  |
| 0 | Vitvi10g04062\_t001 |  |  |  |  |  |  |  |  |  |  |  |  |
| 0 | Vitvi10g00149\_t001 |  |  |  |  |  |  |  |  |  |  |  |  |
| 0 | Vitvi10g04063\_t001 |  |  |  |  |  |  |  |  |  |  |  |  |
| 0 | Vitvi10g04064\_t001 |  |  |  |  |  |  |  |  |  |  |  |  |
| 0 | Vitvi10g00158\_t001 |  |  |  |  |  |  |  |  |  |  |  |  |
| 0 | Vitvi10g00159\_t001 |  |  |  |  |  |  |  |  |  |  |  |  |
| 0 | Vitvi10g04065\_t001 |  |  |  |  |  |  |  |  |  |  |  |  |
| 0 | Vitvi10g04066\_t001 |  |  |  |  |  |  |  |  |  |  |  |  |
| 0 | Vitvi10g04067\_t001 |  |  |  |  |  |  |  |  |  |  |  |  |
| 0 | Vitvi10g04068\_t001 |  |  |  |  |  |  |  |  |  |  |  |  |
| 0 | Vitvi10g04069\_t001 |  |  |  |  |  |  |  |  |  |  |  |  |
| 1 | Vitvi10g00164\_t001 |  | Vitvi19g00561\_t001 |  |  |  |  |  |  |  |  |  |  |
| 1 | Vitvi10g04070\_t001 |  | \| \| |  |  |  |  |  |  |  |  |  |  |
| 1 | Vitvi10g04071\_t001 |  | \| \| |  |  |  |  |  |  |  |  |  |  |
| 1 | Vitvi10g04072\_t001 |  | \| \| |  |  |  |  |  |  |  |  |  |  |
| 1 | Vitvi10g04073\_t001 |  | Vitvi19g00568\_t001 |  |  |  |  |  |  |  |  |  |  |
| 1 | Vitvi10g00167\_t001 |  | \| \| |  |  |  |  |  |  |  |  |  |  |
| 1 | Vitvi10g04074\_t001 |  | \| \| |  |  |  |  |  |  |  |  |  |  |
| 1 | Vitvi10g04075\_t001 |  | \| \| |  |  |  |  |  |  |  |  |  |  |
| 1 | Vitvi10g00174\_t002 |  | Vitvi19g00570\_t001 |  |  |  |  |  |  |  |  |  |  |
| 1 | Vitvi10g04076\_t001 |  | \| \| |  |  |  |  |  |  |  |  |  |  |
| 1 | Vitvi10g01665\_t001 |  | \| \| |  |  |  |  |  |  |  |  |  |  |
| 1 | Vitvi10g01666\_t001 |  | \| \| |  |  |  |  |  |  |  |  |  |  |
| 1 | Vitvi10g00176\_t001 |  | \| \| |  |  |  |  |  |  |  |  |  |  |
| 1 | Vitvi10g00177\_t001 |  | \| \| |  |  |  |  |  |  |  |  |  |  |
| 1 | Vitvi10g00178\_t001 |  | \| \| |  |  |  |  |  |  |  |  |  |  |
| 1 | Vitvi10g00179\_t001 |  | \| \| |  |  |  |  |  |  |  |  |  |  |
| 1 | Vitvi10g00182\_t001 |  | \| \| |  |  |  |  |  |  |  |  |  |  |
| 1 | Vitvi10g01667\_t001 |  | Vitvi19g00580\_t001 |  |  |  |  |  |  |  |  |  |  |
| 1 | Vitvi10g00183\_t001 |  | Vitvi19g00585\_t001 |  |  |  |  |  |  |  |  |  |  |
| 1 | Vitvi10g04077\_t001 |  | \| \| |  |  |  |  |  |  |  |  |  |  |
| 1 | Vitvi10g00185\_t001 |  | \| \| |  |  |  |  |  |  |  |  |  |  |
| 1 | Vitvi10g00186\_t001 |  | \| \| |  |  |  |  |  |  |  |  |  |  |
| 1 | Vitvi10g00187\_t001 |  | \| \| |  |  |  |  |  |  |  |  |  |  |
| 1 | Vitvi10g04078\_t001 |  | Vitvi19g00586\_t001 |  |  |  |  |  |  |  |  |  |  |
| 1 | Vitvi10g04079\_t001 |  | \| \| |  |  |  |  |  |  |  |  |  |  |
| 1 | Vitvi10g00191\_t001 |  | Vitvi19g00590\_t001 |  |  |  |  |  |  |  |  |  |  |
| 1 | Vitvi10g04080\_t001 |  | Vitvi19g00591\_t001 |  |  |  |  |  |  |  |  |  |  |
| 0 | Vitvi10g04081\_t001 |  |  |  |  |  |  |  |  |  |  |  |  |
| 0 | Vitvi10g04082\_t001 |  |  |  |  |  |  |  |  |  |  |  |  |
| 0 | Vitvi10g00194\_t001 |  |  |  |  |  |  |  |  |  |  |  |  |
| 0 | Vitvi10g00195\_t001 |  |  |  |  |  |  |  |  |  |  |  |  |
| 0 | Vitvi10g00196\_t001 |  |  |  |  |  |  |  |  |  |  |  |  |
| 0 | Vitvi10g04083\_t001 |  |  |  |  |  |  |  |  |  |  |  |  |
| 0 | Vitvi10g00197\_t001 |  |  |  |  |  |  |  |  |  |  |  |  |
| 0 | Vitvi10g04084\_t001 |  |  |  |  |  |  |  |  |  |  |  |  |
| 0 | Vitvi10g04085\_t001 |  |  |  |  |  |  |  |  |  |  |  |  |
| 0 | Vitvi10g00198\_t001 |  |  |  |  |  |  |  |  |  |  |  |  |
| 0 | Vitvi10g04086\_t001 |  |  |  |  |  |  |  |  |  |  |  |  |
| 0 | Vitvi10g00204\_t001 |  |  |  |  |  |  |  |  |  |  |  |  |
| 0 | Vitvi10g01674\_t001 |  |  |  |  |  |  |  |  |  |  |  |  |
| 0 | Vitvi10g01675\_t001 |  |  |  |  |  |  |  |  |  |  |  |  |
| 0 | Vitvi10g01676\_t001 |  |  |  |  |  |  |  |  |  |  |  |  |
| 0 | Vitvi10g01678\_t001 |  |  |  |  |  |  |  |  |  |  |  |  |
| 0 | Vitvi10g04087\_t001 |  |  |  |  |  |  |  |  |  |  |  |  |
| 0 | Vitvi10g04088\_t001 |  |  |  |  |  |  |  |  |  |  |  |  |
| 0 | Vitvi10g01679\_t001 |  |  |  |  |  |  |  |  |  |  |  |  |
| 0 | Vitvi10g04089\_t001 |  |  |  |  |  |  |  |  |  |  |  |  |
| 0 | Vitvi10g04090\_t001 |  |  |  |  |  |  |  |  |  |  |  |  |
| 0 | Vitvi10g04091\_t001 |  |  |  |  |  |  |  |  |  |  |  |  |
| 0 | Vitvi10g04092\_t001 |  |  |  |  |  |  |  |  |  |  |  |  |
| 0 | Vitvi10g01682\_t001 |  |  |  |  |  |  |  |  |  |  |  |  |
| 0 | Vitvi10g00209\_t001 |  |  |  |  |  |  |  |  |  |  |  |  |
| 0 | Vitvi10g01683\_t001 |  |  |  |  |  |  |  |  |  |  |  |  |
| 0 | Vitvi10g01684\_t001 |  |  |  |  |  |  |  |  |  |  |  |  |
| 0 | Vitvi10g00210\_t001 |  |  |  |  |  |  |  |  |  |  |  |  |
| 0 | Vitvi10g04093\_t001 |  |  |  |  |  |  |  |  |  |  |  |  |
| 0 | Vitvi10g00212\_t001 |  |  |  |  |  |  |  |  |  |  |  |  |
| 0 | Vitvi10g00213\_t001 |  |  |  |  |  |  |  |  |  |  |  |  |
| 0 | Vitvi10g01685\_t001 |  |  |  |  |  |  |  |  |  |  |  |  |
| 0 | Vitvi10g04094\_t001 |  |  |  |  |  |  |  |  |  |  |  |  |
| 0 | Vitvi10g00214\_t002 |  |  |  |  |  |  |  |  |  |  |  |  |
| 0 | Vitvi10g01686\_t001 |  |  |  |  |  |  |  |  |  |  |  |  |
| 0 | Vitvi10g00215\_t001 |  |  |  |  |  |  |  |  |  |  |  |  |
| 0 | Vitvi10g01687\_t001 |  |  |  |  |  |  |  |  |  |  |  |  |
| 0 | Vitvi10g00217\_t001 |  |  |  |  |  |  |  |  |  |  |  |  |
| 0 | Vitvi10g04095\_t002 |  |  |  |  |  |  |  |  |  |  |  |  |
| 0 | Vitvi10g04096\_t001 |  |  |  |  |  |  |  |  |  |  |  |  |
| 0 | Vitvi10g00219\_t001 |  |  |  |  |  |  |  |  |  |  |  |  |
| 0 | Vitvi10g00220\_t001 |  |  |  |  |  |  |  |  |  |  |  |  |
| 0 | Vitvi10g04097\_t001 |  |  |  |  |  |  |  |  |  |  |  |  |
| 0 | Vitvi10g00221\_t001 |  |  |  |  |  |  |  |  |  |  |  |  |
| 0 | Vitvi10g04098\_t001 |  |  |  |  |  |  |  |  |  |  |  |  |
| 0 | Vitvi10g00222\_t001 |  |  |  |  |  |  |  |  |  |  |  |  |
| 0 | Vitvi10g00223\_t001 |  |  |  |  |  |  |  |  |  |  |  |  |
| 0 | Vitvi10g00224\_t001 |  |  |  |  |  |  |  |  |  |  |  |  |
| 0 | Vitvi10g00225\_t003 |  |  |  |  |  |  |  |  |  |  |  |  |
| 0 | Vitvi10g00226\_t001 |  |  |  |  |  |  |  |  |  |  |  |  |
| 0 | Vitvi10g00227\_t001 |  |  |  |  |  |  |  |  |  |  |  |  |
| 0 | Vitvi10g00228\_t001 |  |  |  |  |  |  |  |  |  |  |  |  |
| 0 | Vitvi10g00229\_t001 |  |  |  |  |  |  |  |  |  |  |  |  |
| 0 | Vitvi10g01689\_t001 |  |  |  |  |  |  |  |  |  |  |  |  |
| 0 | Vitvi10g04099\_t001 |  |  |  |  |  |  |  |  |  |  |  |  |
| 0 | Vitvi10g00230\_t001 |  |  |  |  |  |  |  |  |  |  |  |  |
| 0 | Vitvi10g04100\_t001 |  |  |  |  |  |  |  |  |  |  |  |  |
| 0 | Vitvi10g04101\_t001 |  |  |  |  |  |  |  |  |  |  |  |  |
| 0 | Vitvi10g04102\_t001 |  |  |  |  |  |  |  |  |  |  |  |  |
| 0 | Vitvi10g04103\_t001 |  |  |  |  |  |  |  |  |  |  |  |  |
| 0 | Vitvi10g01696\_t001 |  |  |  |  |  |  |  |  |  |  |  |  |
| 0 | Vitvi10g04104\_t001 |  |  |  |  |  |  |  |  |  |  |  |  |
| 0 | Vitvi10g00231\_t001 |  |  |  |  |  |  |  |  |  |  |  |  |
| 0 | Vitvi10g04105\_t001 |  |  |  |  |  |  |  |  |  |  |  |  |
| 1 | Vitvi10g00232\_t001 |  | Vitvi00g04639\_t001 |  |  |  |  |  |  |  |  |  |  |
| 1 | Vitvi10g01697\_t001 |  | \| \| |  |  |  |  |  |  |  |  |  |  |
| 1 | Vitvi10g04106\_t001 |  | \| \| |  |  |  |  |  |  |  |  |  |  |
| 1 | Vitvi10g01698\_t001 |  | \| \| |  |  |  |  |  |  |  |  |  |  |
| 1 | Vitvi10g00233\_t001 |  | \| \| |  |  |  |  |  |  |  |  |  |  |
| 1 | Vitvi10g04107\_t001 |  | \| \| |  |  |  |  |  |  |  |  |  |  |
| 1 | Vitvi10g04108\_t001 |  | Vitvi00g04640\_t001 |  |  |  |  |  |  |  |  |  |  |
| 1 | Vitvi10g04109\_t001 |  | Vitvi00g04641\_t001 |  |  |  |  |  |  |  |  |  |  |
| 1 | Vitvi10g04110\_t001 |  | \| \| |  |  |  |  |  |  |  |  |  |  |
| 1 | Vitvi10g01699\_t001 |  | Vitvi00g04642\_t001 |  |  |  |  |  |  |  |  |  |  |
| 1 | Vitvi10g01700\_t001 |  | \| \| |  |  |  |  |  |  |  |  |  |  |
| 1 | Vitvi10g00235\_t001 |  | \| \| |  |  |  |  |  |  |  |  |  |  |
| 1 | Vitvi10g04111\_t001 |  | \| \| |  |  |  |  |  |  |  |  |  |  |
| 1 | Vitvi10g04112\_t001 |  | \| \| |  |  |  |  |  |  |  |  |  |  |
| 1 | Vitvi10g04113\_t001 |  | \| \| |  |  |  |  |  |  |  |  |  |  |
| 1 | Vitvi10g04114\_t001 |  | \| \| |  |  |  |  |  |  |  |  |  |  |
| 1 | Vitvi10g04115\_t001 |  | Vitvi10g02309\_t001 |  |  |  |  |  |  |  |  |  |  |
| 1 | Vitvi10g02306\_t001 |  | \| \| |  |  |  |  |  |  |  |  |  |  |
| 1 | Vitvi10g04116\_t001 |  | \| \| |  |  |  |  |  |  |  |  |  |  |
| 1 | Vitvi10g04117\_t001 |  | \| \| |  |  |  |  |  |  |  |  |  |  |
| 1 | Vitvi10g00258\_t002 |  | \| \| |  |  |  |  |  |  |  |  |  |  |
| 1 | Vitvi10g00256\_t001 |  | \| \| |  |  |  |  |  |  |  |  |  |  |
| 1 | Vitvi10g00255\_t001.2.6037826a |  | \| \| |  |  |  |  |  |  |  |  |  |  |
| 1 | Vitvi10g00259\_t001 |  | \| \| |  |  |  |  |  |  |  |  |  |  |
| 1 | Vitvi10g04118\_t001 |  | \| \| |  |  |  |  |  |  |  |  |  |  |
| 1 | Vitvi10g04119\_t001 |  | \| \| |  |  |  |  |  |  |  |  |  |  |
| 1 | Vitvi10g00264\_t001 |  | \| \| |  |  |  |  |  |  |  |  |  |  |
| 1 | Vitvi10g04120\_t002 |  | \| \| |  |  |  |  |  |  |  |  |  |  |
| 1 | Vitvi10g00263\_t001 |  | \| \| |  |  |  |  |  |  |  |  |  |  |
| 1 | Vitvi10g04121\_t001 |  | \| \| |  |  |  |  |  |  |  |  |  |  |
| 1 | Vitvi10g04122\_t001 |  | \| \| |  |  |  |  |  |  |  |  |  |  |
| 1 | Vitvi10g00261\_t001 |  | \| \| |  |  |  |  |  |  |  |  |  |  |
| 1 | Vitvi10g01702\_t001 |  | \| \| |  |  |  |  |  |  |  |  |  |  |
| 1 | Vitvi10g01703\_t001 |  | Vitvi10g02308\_t001 |  |  |  |  |  |  |  |  |  |  |
| 1 | Vitvi10g04123\_t001 |  | \| \| |  |  |  |  |  |  |  |  |  |  |
| 1 | Vitvi10g01704\_t001 |  | \| \| |  |  |  |  |  |  |  |  |  |  |
| 1 | Vitvi10g00239\_t001 |  | \| \| |  |  |  |  |  |  |  |  |  |  |
| 1 | Vitvi10g01706\_t001 |  | \| \| |  |  |  |  |  |  |  |  |  |  |
| 1 | Vitvi10g00241\_t001 |  | Vitvi00g04656\_t001 |  |  |  |  |  |  |  |  |  |  |
| 0 | Vitvi10g04124\_t001 |  |  |  |  |  |  |  |  |  |  |  |  |
| 0 | Vitvi10g00245\_t001 |  |  |  |  |  |  |  |  |  |  |  |  |
| 0 | Vitvi10g00246\_t001 |  |  |  |  |  |  |  |  |  |  |  |  |
| 0 | Vitvi10g04125\_t001 |  |  |  |  |  |  |  |  |  |  |  |  |
| 0 | Vitvi10g00247\_t001 |  |  |  |  |  |  |  |  |  |  |  |  |
| 0 | Vitvi10g00248\_t001 |  |  |  |  |  |  |  |  |  |  |  |  |
| 0 | Vitvi10g00249\_t001 |  |  |  |  |  |  |  |  |  |  |  |  |
| 0 | Vitvi10g01710\_t001 |  |  |  |  |  |  |  |  |  |  |  |  |
| 0 | Vitvi10g00251\_t001 |  |  |  |  |  |  |  |  |  |  |  |  |
| 0 | Vitvi10g00252\_t001 |  |  |  |  |  |  |  |  |  |  |  |  |
| 0 | Vitvi10g02271\_t001 |  |  |  |  |  |  |  |  |  |  |  |  |
| 0 | Vitvi10g02272\_t001 |  |  |  |  |  |  |  |  |  |  |  |  |
| 0 | Vitvi10g02273\_t001 |  |  |  |  |  |  |  |  |  |  |  |  |
| 0 | Vitvi10g04126\_t001 |  |  |  |  |  |  |  |  |  |  |  |  |
| 0 | Vitvi10g04127\_t001 |  |  |  |  |  |  |  |  |  |  |  |  |
| 0 | Vitvi10g04128\_t001 |  |  |  |  |  |  |  |  |  |  |  |  |
| 0 | Vitvi10g02267\_t001 |  |  |  |  |  |  |  |  |  |  |  |  |
| 0 | Vitvi10g02268\_t001 |  |  |  |  |  |  |  |  |  |  |  |  |
| 0 | Vitvi10g02269\_t001 |  |  |  |  |  |  |  |  |  |  |  |  |
| 0 | Vitvi10g04129\_t001 |  |  |  |  |  |  |  |  |  |  |  |  |
| 0 | Vitvi10g02386\_t002 |  |  |  |  |  |  |  |  |  |  |  |  |
| 0 | Vitvi10g04130\_t001 |  |  |  |  |  |  |  |  |  |  |  |  |
| 0 | Vitvi10g04131\_t001 |  |  |  |  |  |  |  |  |  |  |  |  |
| 0 | Vitvi10g04132\_t001 |  |  |  |  |  |  |  |  |  |  |  |  |
| 0 | Vitvi10g02282\_t001 |  |  |  |  |  |  |  |  |  |  |  |  |
| 0 | Vitvi10g02283\_t001 |  |  |  |  |  |  |  |  |  |  |  |  |
| 0 | Vitvi10g02284\_t001 |  |  |  |  |  |  |  |  |  |  |  |  |
| 0 | Vitvi10g04133\_t001 |  |  |  |  |  |  |  |  |  |  |  |  |
| 0 | Vitvi10g04134\_t001 |  |  |  |  |  |  |  |  |  |  |  |  |
| 0 | Vitvi10g00275\_t001 |  |  |  |  |  |  |  |  |  |  |  |  |
| 0 | Vitvi10g00274\_t001 |  |  |  |  |  |  |  |  |  |  |  |  |
| 0 | Vitvi10g04135\_t001 |  |  |  |  |  |  |  |  |  |  |  |  |
| 0 | Vitvi10g04136\_t001 |  |  |  |  |  |  |  |  |  |  |  |  |
| 0 | Vitvi10g04137\_t001 |  |  |  |  |  |  |  |  |  |  |  |  |
| 1 | Vitvi10g00271\_t001 |  | Vitvi12g00665\_t002 |  |  |  |  |  |  |  |  |  |  |
| 1 | Vitvi10g00270\_t001 |  | Vitvi12g00664\_t003 |  |  |  |  |  |  |  |  |  |  |
| 1 | Vitvi10g04138\_t001 |  | \| \| |  |  |  |  |  |  |  |  |  |  |
| 1 | Vitvi10g04139\_t001 |  | \| \| |  |  |  |  |  |  |  |  |  |  |
| 1 | Vitvi10g00278\_t001 |  | \| \| |  |  |  |  |  |  |  |  |  |  |
| 1 | Vitvi10g04140\_t001 |  | Vitvi12g00657\_t001 |  |  |  |  |  |  |  |  |  |  |
| 1 | Vitvi10g04141\_t001 |  | Vitvi12g00656\_t001 |  |  |  |  |  |  |  |  |  |  |
| 1 | Vitvi10g00282\_t001 |  | \| \| |  |  |  |  |  |  |  |  |  |  |
| 1 | Vitvi10g04142\_t001 |  | \| \| |  |  |  |  |  |  |  |  |  |  |
| 1 | Vitvi10g00288\_t001 |  | \| \| |  |  |  |  |  |  |  |  |  |  |
| 1 | Vitvi10g04143\_t001 |  | \| \| |  |  |  |  |  |  |  |  |  |  |
| 1 | Vitvi10g04144\_t001 |  | \| \| |  |  |  |  |  |  |  |  |  |  |
| 1 | Vitvi10g00286\_t001 |  | Vitvi12g00649\_t001 |  |  |  |  |  |  |  |  |  |  |
| 1 | Vitvi10g00285\_t002 |  | \| \| |  |  |  |  |  |  |  |  |  |  |
| 1 | Vitvi10g00284\_t001 |  | Vitvi12g00648\_t001 |  |  |  |  |  |  |  |  |  |  |
| 0 | Vitvi10g04145\_t001 |  |  |  |  |  |  |  |  |  |  |  |  |
| 0 | Vitvi10g02300\_t001 |  |  |  |  |  |  |  |  |  |  |  |  |
| 0 | Vitvi10g02302\_t001 |  |  |  |  |  |  |  |  |  |  |  |  |
| 0 | Vitvi10g02298\_t001 |  |  |  |  |  |  |  |  |  |  |  |  |
| 0 | Vitvi10g04146\_t001 |  |  |  |  |  |  |  |  |  |  |  |  |
| 0 | Vitvi10g04147\_t001 |  |  |  |  |  |  |  |  |  |  |  |  |
| 0 | Vitvi10g02296\_t001 |  |  |  |  |  |  |  |  |  |  |  |  |
| 0 | Vitvi10g02295\_t001 |  |  |  |  |  |  |  |  |  |  |  |  |
| 0 | Vitvi10g04148\_t001 |  |  |  |  |  |  |  |  |  |  |  |  |
| 0 | Vitvi10g00304\_t001 |  |  |  |  |  |  |  |  |  |  |  |  |
| 0 | Vitvi10g00306\_t001 |  |  |  |  |  |  |  |  |  |  |  |  |
| 0 | Vitvi10g04149\_t001 |  |  |  |  |  |  |  |  |  |  |  |  |
| 0 | Vitvi10g00307\_t003 |  |  |  |  |  |  |  |  |  |  |  |  |
| 0 | Vitvi10g04150\_t001 |  |  |  |  |  |  |  |  |  |  |  |  |
| 0 | Vitvi10g04151\_t001 |  |  |  |  |  |  |  |  |  |  |  |  |
| 0 | Vitvi10g04152\_t001 |  |  |  |  |  |  |  |  |  |  |  |  |
| 0 | Vitvi10g04153\_t001 |  |  |  |  |  |  |  |  |  |  |  |  |
| 0 | Vitvi10g04154\_t001 |  |  |  |  |  |  |  |  |  |  |  |  |
| 0 | Vitvi10g00302\_t001 |  |  |  |  |  |  |  |  |  |  |  |  |
| 0 | Vitvi10g04155\_t001 |  |  |  |  |  |  |  |  |  |  |  |  |
| 0 | Vitvi10g04156\_t001 |  |  |  |  |  |  |  |  |  |  |  |  |
| 0 | Vitvi10g04157\_t001 |  |  |  |  |  |  |  |  |  |  |  |  |
| 0 | Vitvi10g04158\_t001 |  |  |  |  |  |  |  |  |  |  |  |  |
| 0 | Vitvi10g04159\_t001 |  |  |  |  |  |  |  |  |  |  |  |  |
| 0 | Vitvi10g02133\_t001 |  |  |  |  |  |  |  |  |  |  |  |  |
| 0 | Vitvi10g04160\_t001 |  |  |  |  |  |  |  |  |  |  |  |  |
| 0 | Vitvi10g02135\_t001 |  |  |  |  |  |  |  |  |  |  |  |  |
| 0 | Vitvi10g04161\_t001 |  |  |  |  |  |  |  |  |  |  |  |  |
| 0 | Vitvi10g04162\_t001 |  |  |  |  |  |  |  |  |  |  |  |  |
| 0 | Vitvi10g04163\_t001 |  |  |  |  |  |  |  |  |  |  |  |  |
| 0 | Vitvi10g04164\_t001 |  |  |  |  |  |  |  |  |  |  |  |  |
| 0 | Vitvi10g04165\_t001 |  |  |  |  |  |  |  |  |  |  |  |  |
| 0 | Vitvi10g04166\_t001 |  |  |  |  |  |  |  |  |  |  |  |  |
| 0 | Vitvi10g04167\_t001 |  |  |  |  |  |  |  |  |  |  |  |  |
| 0 | Vitvi10g02145\_t001 |  |  |  |  |  |  |  |  |  |  |  |  |
| 0 | Vitvi10g04168\_t001 |  |  |  |  |  |  |  |  |  |  |  |  |
| 0 | Vitvi10g04169\_t001 |  |  |  |  |  |  |  |  |  |  |  |  |
| 0 | Vitvi10g04170\_t001 |  |  |  |  |  |  |  |  |  |  |  |  |
| 0 | Vitvi10g04171\_t001 |  |  |  |  |  |  |  |  |  |  |  |  |
| 0 | Vitvi10g04172\_t001 |  |  |  |  |  |  |  |  |  |  |  |  |
| 0 | Vitvi10g04173\_t001 |  |  |  |  |  |  |  |  |  |  |  |  |
| 0 | Vitvi10g04174\_t001 |  |  |  |  |  |  |  |  |  |  |  |  |
| 0 | Vitvi10g04175\_t001 |  |  |  |  |  |  |  |  |  |  |  |  |
| 0 | Vitvi10g04176\_t001 |  |  |  |  |  |  |  |  |  |  |  |  |
| 0 | Vitvi10g04177\_t001 |  |  |  |  |  |  |  |  |  |  |  |  |
| 0 | Vitvi10g02151\_t001 |  |  |  |  |  |  |  |  |  |  |  |  |
| 0 | Vitvi10g04178\_t001 |  |  |  |  |  |  |  |  |  |  |  |  |
| 0 | Vitvi10g04179\_t001 |  |  |  |  |  |  |  |  |  |  |  |  |
| 0 | Vitvi10g04180\_t001 |  |  |  |  |  |  |  |  |  |  |  |  |
| 0 | Vitvi10g04181\_t001 |  |  |  |  |  |  |  |  |  |  |  |  |
| 0 | Vitvi10g04182\_t001 |  |  |  |  |  |  |  |  |  |  |  |  |
| 0 | Vitvi10g04183\_t001 |  |  |  |  |  |  |  |  |  |  |  |  |
| 0 | Vitvi10g04184\_t001 |  |  |  |  |  |  |  |  |  |  |  |  |
| 0 | Vitvi10g04185\_t001 |  |  |  |  |  |  |  |  |  |  |  |  |
| 0 | Vitvi10g04186\_t001 |  |  |  |  |  |  |  |  |  |  |  |  |
| 0 | Vitvi10g04187\_t001 |  |  |  |  |  |  |  |  |  |  |  |  |
| 0 | Vitvi10g04188\_t001 |  |  |  |  |  |  |  |  |  |  |  |  |
| 0 | Vitvi10g04189\_t001 |  |  |  |  |  |  |  |  |  |  |  |  |
| 0 | Vitvi10g04190\_t001 |  |  |  |  |  |  |  |  |  |  |  |  |
| 0 | Vitvi10g04191\_t001 |  |  |  |  |  |  |  |  |  |  |  |  |
| 0 | Vitvi10g02176\_t001 |  |  |  |  |  |  |  |  |  |  |  |  |
| 1 | Vitvi10g02177\_t001 |  | Vitvi12g00572\_t001 |  |  |  |  |  |  |  |  |  |  |
| 1 | Vitvi10g02178\_t001 |  | Vitvi12g00569\_t002 |  |  |  |  |  |  |  |  |  |  |
| 1 | Vitvi10g04192\_t001 |  | \| \| |  |  |  |  |  |  |  |  |  |  |
| 1 | Vitvi10g04193\_t001 |  | \| \| |  |  |  |  |  |  |  |  |  |  |
| 1 | Vitvi10g02191\_t001 |  | \| \| |  |  |  |  |  |  |  |  |  |  |
| 2 | Vitvi10g02192\_t001 |  | \| \| |  | Vitvi19g00394\_t001 |  |  |  |  |  |  |  |  |
| 2 | Vitvi10g02193\_t001 |  | \| \| |  | \| \| |  |  |  |  |  |  |  |  |
| 2 | Vitvi10g04194\_t001 |  | \| \| |  | \| \| |  |  |  |  |  |  |  |  |
| 2 | Vitvi10g02195\_t001 |  | \| \| |  | \| \| |  |  |  |  |  |  |  |  |
| 2 | Vitvi10g04195\_t001 |  | \| \| |  | \| \| |  |  |  |  |  |  |  |  |
| 2 | Vitvi10g02185\_t001 |  | \| \| |  | \| \| |  |  |  |  |  |  |  |  |
| 2 | Vitvi10g04196\_t001 |  | \| \| |  | \| \| |  |  |  |  |  |  |  |  |
| 2 | Vitvi10g02187\_t001 |  | \| \| |  | \| \| |  |  |  |  |  |  |  |  |
| 2 | Vitvi10g00290\_t001 |  | \| \| |  | Vitvi19g00397\_t001 |  |  |  |  |  |  |  |  |
| 2 | Vitvi10g04197\_t001 |  | \| \| |  | \| \| |  |  |  |  |  |  |  |  |
| 2 | Vitvi10g02382\_t001 |  | \| \| |  | \| \| |  |  |  |  |  |  |  |  |
| 2 | Vitvi10g02202\_t001 |  | Vitvi12g00566\_t001 |  | Vitvi19g01977\_t001 |  |  |  |  |  |  |  |  |
| 2 | Vitvi10g02201\_t001 |  | \| \| |  | Vitvi19g00401\_t001 |  |  |  |  |  |  |  |  |
| 2 | Vitvi10g04198\_t001 |  | \| \| |  | \| \| |  |  |  |  |  |  |  |  |
| 2 | Vitvi10g02200\_t001 |  | Vitvi12g00561\_t001 |  | \| \| |  |  |  |  |  |  |  |  |
| 2 | Vitvi10g04199\_t001 |  | \| \| |  | \| \| |  |  |  |  |  |  |  |  |
| 2 | Vitvi10g04200\_t001 |  | \| \| |  | \| \| |  |  |  |  |  |  |  |  |
| 2 | Vitvi10g04201\_t001 |  | \| \| |  | \| \| |  |  |  |  |  |  |  |  |
| 2 | Vitvi10g02211\_t001 |  | \| \| |  | \| \| |  |  |  |  |  |  |  |  |
| 2 | Vitvi10g02212\_t001 |  | \| \| |  | Vitvi19g01981\_t001 |  |  |  |  |  |  |  |  |
| 2 | Vitvi10g02215\_t001 |  | \| \| |  | \| \| |  |  |  |  |  |  |  |  |
| 2 | Vitvi10g02216\_t001 |  | \| \| |  | Vitvi19g00405\_t001 |  |  |  |  |  |  |  |  |
| 2 | Vitvi10g02218\_t001 |  | Vitvi12g00556\_t002 |  | Vitvi19g00406\_t001 |  |  |  |  |  |  |  |  |
| 1 | Vitvi10g02219\_t001 |  | \| \| |  |  |  |  |  |  |  |  |  |  |
| 1 | Vitvi10g02220\_t001 |  | Vitvi12g02419\_t001 |  |  |  |  |  |  |  |  |  |  |
| 1 | Vitvi10g04202\_t001 |  | \| \| |  |  |  |  |  |  |  |  |  |  |
| 1 | Vitvi10g02222\_t001 |  | \| \| |  |  |  |  |  |  |  |  |  |  |
| 1 | Vitvi10g04203\_t001 |  | \| \| |  |  |  |  |  |  |  |  |  |  |
| 1 | Vitvi10g01729\_t001 |  | \| \| |  |  |  |  |  |  |  |  |  |  |
| 1 | Vitvi10g01728\_t001 |  | \| \| |  |  |  |  |  |  |  |  |  |  |
| 1 | Vitvi10g02227\_t001 |  | \| \| |  |  |  |  |  |  |  |  |  |  |
| 1 | Vitvi10g02229\_t001 |  | \| \| |  |  |  |  |  |  |  |  |  |  |
| 1 | Vitvi10g04204\_t001 |  | \| \| |  |  |  |  |  |  |  |  |  |  |
| 1 | Vitvi10g02225\_t001 |  | \| \| |  |  |  |  |  |  |  |  |  |  |
| 1 | Vitvi10g04205\_t001 |  | \| \| |  |  |  |  |  |  |  |  |  |  |
| 1 | Vitvi10g04206\_t001 |  | \| \| |  |  |  |  |  |  |  |  |  |  |
| 1 | Vitvi10g04207\_t001 |  | \| \| |  |  |  |  |  |  |  |  |  |  |
| 1 | Vitvi10g02236\_t001 |  | \| \| |  |  |  |  |  |  |  |  |  |  |
| 1 | Vitvi10g02237\_t001 |  | \| \| |  |  |  |  |  |  |  |  |  |  |
| 1 | Vitvi10g04208\_t001 |  | \| \| |  |  |  |  |  |  |  |  |  |  |
| 1 | Vitvi10g04209\_t001 |  | Vitvi12g00549\_t001 |  |  |  |  |  |  |  |  |  |  |
| 1 | Vitvi10g04210\_t001 |  | \| \| |  |  |  |  |  |  |  |  |  |  |
| 1 | Vitvi10g02240\_t001 |  | \| \| |  |  |  |  |  |  |  |  |  |  |
| 1 | Vitvi10g04211\_t001 |  | \| \| |  |  |  |  |  |  |  |  |  |  |
| 1 | Vitvi10g04212\_t001 |  | \| \| |  |  |  |  |  |  |  |  |  |  |
| 1 | Vitvi10g02245\_t001 |  | \| \| |  |  |  |  |  |  |  |  |  |  |
| 1 | Vitvi10g02246\_t001 |  | \| \| |  |  |  |  |  |  |  |  |  |  |
| 1 | Vitvi10g02247\_t001 |  | \| \| |  |  |  |  |  |  |  |  |  |  |
| 1 | Vitvi10g02248\_t001 |  | \| \| |  |  |  |  |  |  |  |  |  |  |
| 1 | Vitvi10g04213\_t001 |  | Vitvi12g04165\_t001 |  |  |  |  |  |  |  |  |  |  |
| 0 | Vitvi10g04214\_t001 |  |  |  |  |  |  |  |  |  |  |  |  |
| 0 | Vitvi10g04215\_t001 |  |  |  |  |  |  |  |  |  |  |  |  |
| 0 | Vitvi10g04216\_t001 |  |  |  |  |  |  |  |  |  |  |  |  |
| 0 | Vitvi10g02129\_t001 |  |  |  |  |  |  |  |  |  |  |  |  |
| 0 | Vitvi10g04217\_t001 |  |  |  |  |  |  |  |  |  |  |  |  |
| 0 | Vitvi10g04218\_t001 |  |  |  |  |  |  |  |  |  |  |  |  |
| 0 | Vitvi10g04219\_t001 |  |  |  |  |  |  |  |  |  |  |  |  |
| 0 | Vitvi10g04220\_t001 |  |  |  |  |  |  |  |  |  |  |  |  |
| 0 | Vitvi10g04221\_t001 |  |  |  |  |  |  |  |  |  |  |  |  |
| 0 | Vitvi10g04222\_t001 |  |  |  |  |  |  |  |  |  |  |  |  |
| 0 | Vitvi10g04223\_t001 |  |  |  |  |  |  |  |  |  |  |  |  |
| 0 | Vitvi10g04224\_t001 |  |  |  |  |  |  |  |  |  |  |  |  |
| 0 | Vitvi10g02166\_t001 |  |  |  |  |  |  |  |  |  |  |  |  |
| 0 | Vitvi10g02165\_t001 |  |  |  |  |  |  |  |  |  |  |  |  |
| 0 | Vitvi10g02164\_t001 |  |  |  |  |  |  |  |  |  |  |  |  |
| 0 | Vitvi10g00321\_t001 |  |  |  |  |  |  |  |  |  |  |  |  |
| 0 | Vitvi10g04225\_t001 |  |  |  |  |  |  |  |  |  |  |  |  |
| 0 | Vitvi10g00323\_t001 |  |  |  |  |  |  |  |  |  |  |  |  |
| 0 | Vitvi10g01734\_t001 |  |  |  |  |  |  |  |  |  |  |  |  |
| 0 | Vitvi10g01735\_t001 |  |  |  |  |  |  |  |  |  |  |  |  |
| 0 | Vitvi10g04226\_t001 |  |  |  |  |  |  |  |  |  |  |  |  |
| 0 | Vitvi10g04227\_t001 |  |  |  |  |  |  |  |  |  |  |  |  |
| 0 | Vitvi10g04228\_t001 |  |  |  |  |  |  |  |  |  |  |  |  |
| 0 | Vitvi10g04229\_t001 |  |  |  |  |  |  |  |  |  |  |  |  |
| 0 | Vitvi10g04230\_t001 |  |  |  |  |  |  |  |  |  |  |  |  |
| 0 | Vitvi10g04231\_t001 |  |  |  |  |  |  |  |  |  |  |  |  |
| 1 | Vitvi10g04232\_t001 |  | Vitvi12g00595\_t001 |  |  |  |  |  |  |  |  |  |  |
| 1 | Vitvi10g04233\_t001 |  | \| \| |  |  |  |  |  |  |  |  |  |  |
| 1 | Vitvi10g04234\_t001 |  | \| \| |  |  |  |  |  |  |  |  |  |  |
| 1 | Vitvi10g04235\_t001 |  | \| \| |  |  |  |  |  |  |  |  |  |  |
| 1 | Vitvi10g04236\_t001 |  | \| \| |  |  |  |  |  |  |  |  |  |  |
| 1 | Vitvi10g04237\_t001 |  | \| \| |  |  |  |  |  |  |  |  |  |  |
| 1 | Vitvi10g04238\_t001 |  | \| \| |  |  |  |  |  |  |  |  |  |  |
| 1 | Vitvi10g02322\_t001 |  | \| \| |  |  |  |  |  |  |  |  |  |  |
| 1 | Vitvi10g04239\_t001 |  | \| \| |  |  |  |  |  |  |  |  |  |  |
| 1 | Vitvi10g04240\_t001 |  | \| \| |  |  |  |  |  |  |  |  |  |  |
| 1 | Vitvi10g04241\_t001 |  | \| \| |  |  |  |  |  |  |  |  |  |  |
| 1 | Vitvi10g04242\_t001 |  | \| \| |  |  |  |  |  |  |  |  |  |  |
| 1 | Vitvi10g02328\_t001 |  | Vitvi12g02441\_t001 |  |  |  |  |  |  |  |  |  |  |
| 1 | Vitvi10g00352\_t001 |  | Vitvi12g00620\_t002 |  |  |  |  |  |  |  |  |  |  |
| 1 | Vitvi10g00350\_t001 |  | \| \| |  |  |  |  |  |  |  |  |  |  |
| 1 | Vitvi10g04243\_t001 |  | \| \| |  |  |  |  |  |  |  |  |  |  |
| 1 | Vitvi10g00345\_t001 |  | Vitvi12g00625\_t001 |  |  |  |  |  |  |  |  |  |  |
| 1 | Vitvi10g00344\_t001 |  | Vitvi12g00627\_t002 |  |  |  |  |  |  |  |  |  |  |
| 1 | Vitvi10g00343\_t001 |  | \| \| |  |  |  |  |  |  |  |  |  |  |
| 1 | Vitvi10g00342\_t001 |  | Vitvi12g00630\_t001 |  |  |  |  |  |  |  |  |  |  |
| 0 | Vitvi10g04244\_t001 |  |  |  |  |  |  |  |  |  |  |  |  |
| 0 | Vitvi10g00341\_t001 |  |  |  |  |  |  |  |  |  |  |  |  |
| 0 | Vitvi10g00339\_t001 |  |  |  |  |  |  |  |  |  |  |  |  |
| 0 | Vitvi10g00336\_t001.1.6037826a |  |  |  |  |  |  |  |  |  |  |  |  |
| 0 | Vitvi10g00334\_t001 |  |  |  |  |  |  |  |  |  |  |  |  |
| 0 | Vitvi10g04245\_t001 |  |  |  |  |  |  |  |  |  |  |  |  |
| 0 | Vitvi10g01738\_t001 |  |  |  |  |  |  |  |  |  |  |  |  |
| 0 | Vitvi10g01737\_t001 |  |  |  |  |  |  |  |  |  |  |  |  |
| 0 | Vitvi10g04246\_t001 |  |  |  |  |  |  |  |  |  |  |  |  |
| 0 | Vitvi10g04247\_t001 |  |  |  |  |  |  |  |  |  |  |  |  |
| 0 | Vitvi10g04248\_t001 |  |  |  |  |  |  |  |  |  |  |  |  |
| 0 | Vitvi10g04249\_t001 |  |  |  |  |  |  |  |  |  |  |  |  |
| 0 | Vitvi10g04250\_t001 |  |  |  |  |  |  |  |  |  |  |  |  |
| 0 | Vitvi10g04251\_t001 |  |  |  |  |  |  |  |  |  |  |  |  |
| 0 | Vitvi10g04252\_t001 |  |  |  |  |  |  |  |  |  |  |  |  |
| 0 | Vitvi10g04253\_t001 |  |  |  |  |  |  |  |  |  |  |  |  |
| 0 | Vitvi10g04254\_t001 |  |  |  |  |  |  |  |  |  |  |  |  |
| 0 | Vitvi10g04255\_t001 |  |  |  |  |  |  |  |  |  |  |  |  |
| 0 | Vitvi10g04256\_t001 |  |  |  |  |  |  |  |  |  |  |  |  |
| 0 | Vitvi10g00358\_t001 |  |  |  |  |  |  |  |  |  |  |  |  |
| 0 | Vitvi10g00359\_t001 |  |  |  |  |  |  |  |  |  |  |  |  |
| 0 | Vitvi10g01740\_t001 |  |  |  |  |  |  |  |  |  |  |  |  |
| 0 | Vitvi10g04257\_t001 |  |  |  |  |  |  |  |  |  |  |  |  |
| 0 | Vitvi10g04258\_t001 |  |  |  |  |  |  |  |  |  |  |  |  |
| 0 | Vitvi10g01746\_t001 |  |  |  |  |  |  |  |  |  |  |  |  |
| 0 | Vitvi10g00363\_t001 |  |  |  |  |  |  |  |  |  |  |  |  |
| 0 | Vitvi10g01742\_t001 |  |  |  |  |  |  |  |  |  |  |  |  |
| 0 | Vitvi10g01748\_t001 |  |  |  |  |  |  |  |  |  |  |  |  |
| 0 | Vitvi10g00364\_t001 |  |  |  |  |  |  |  |  |  |  |  |  |
| 0 | Vitvi10g00365\_t001 |  |  |  |  |  |  |  |  |  |  |  |  |
| 0 | Vitvi10g00366\_t001 |  |  |  |  |  |  |  |  |  |  |  |  |
| 0 | Vitvi10g01749\_t001 |  |  |  |  |  |  |  |  |  |  |  |  |
| 0 | Vitvi10g01751\_t001 |  |  |  |  |  |  |  |  |  |  |  |  |
| 0 | Vitvi10g00370\_t001 |  |  |  |  |  |  |  |  |  |  |  |  |
| 1 | Vitvi10g00372\_t001 |  | Vitvi19g00298\_t001 |  |  |  |  |  |  |  |  |  |  |
| 1 | Vitvi10g00379\_t001 |  | \| \| |  |  |  |  |  |  |  |  |  |  |
| 1 | Vitvi10g00380\_t001 |  | \| \| |  |  |  |  |  |  |  |  |  |  |
| 1 | Vitvi10g00381\_t001 |  | \| \| |  |  |  |  |  |  |  |  |  |  |
| 1 | Vitvi10g00382\_t001 |  | \| \| |  |  |  |  |  |  |  |  |  |  |
| 1 | Vitvi10g00383\_t001 |  | \| \| |  |  |  |  |  |  |  |  |  |  |
| 1 | Vitvi10g04259\_t002 |  | Vitvi19g04114\_t001 |  |  |  |  |  |  |  |  |  |  |
| 1 | Vitvi10g00385\_t001 |  | \| \| |  |  |  |  |  |  |  |  |  |  |
| 1 | Vitvi10g00386\_t002 |  | \| \| |  |  |  |  |  |  |  |  |  |  |
| 1 | Vitvi10g00387\_t001 |  | \| \| |  |  |  |  |  |  |  |  |  |  |
| 1 | Vitvi10g04260\_t001 |  | \| \| |  |  |  |  |  |  |  |  |  |  |
| 1 | Vitvi10g00390\_t001 |  | Vitvi19g00291\_t001 |  |  |  |  |  |  |  |  |  |  |
| 1 | Vitvi10g04261\_t001 |  | \| \| |  |  |  |  |  |  |  |  |  |  |
| 1 | Vitvi10g00391\_t001 |  | Vitvi19g00290\_t001 |  |  |  |  |  |  |  |  |  |  |
| 1 | Vitvi10g00394\_t001 |  | Vitvi19g00286\_t001 |  |  |  |  |  |  |  |  |  |  |
| 1 | Vitvi10g04262\_t001 |  | \| \| |  |  |  |  |  |  |  |  |  |  |
| 1 | Vitvi10g01757\_t001 |  | \| \| |  |  |  |  |  |  |  |  |  |  |
| 1 | Vitvi10g04263\_t001 |  | \| \| |  |  |  |  |  |  |  |  |  |  |
| 1 | Vitvi10g00406\_t001 |  | Vitvi19g00282\_t001 |  |  |  |  |  |  |  |  |  |  |
| 1 | Vitvi10g00405\_t001 |  | \| \| |  |  |  |  |  |  |  |  |  |  |
| 1 | Vitvi10g04264\_t001 |  | \| \| |  |  |  |  |  |  |  |  |  |  |
| 1 | Vitvi10g00402\_t001 |  | \| \| |  |  |  |  |  |  |  |  |  |  |
| 1 | Vitvi10g00401\_t001 |  | \| \| |  |  |  |  |  |  |  |  |  |  |
| 1 | Vitvi10g00400\_t001 |  | \| \| |  |  |  |  |  |  |  |  |  |  |
| 1 | Vitvi10g04265\_t001 |  | \| \| |  |  |  |  |  |  |  |  |  |  |
| 1 | Vitvi10g00399\_t001 |  | \| \| |  |  |  |  |  |  |  |  |  |  |
| 1 | Vitvi10g01756\_t001 |  | \| \| |  |  |  |  |  |  |  |  |  |  |
| 1 | Vitvi10g04266\_t001 |  | \| \| |  |  |  |  |  |  |  |  |  |  |
| 1 | Vitvi10g04267\_t001 |  | \| \| |  |  |  |  |  |  |  |  |  |  |
| 1 | Vitvi10g01754\_t001 |  | \| \| |  |  |  |  |  |  |  |  |  |  |
| 1 | Vitvi10g04268\_t001 |  | \| \| |  |  |  |  |  |  |  |  |  |  |
| 1 | Vitvi10g04269\_t001 |  | \| \| |  |  |  |  |  |  |  |  |  |  |
| 1 | Vitvi10g00398\_t001 |  | Vitvi19g00276\_t001 |  |  |  |  |  |  |  |  |  |  |
| 1 | Vitvi10g01753\_t001 |  | \| \| |  |  |  |  |  |  |  |  |  |  |
| 1 | Vitvi10g04270\_t001 |  | \| \| |  |  |  |  |  |  |  |  |  |  |
| 2 | Vitvi10g00397\_t001 |  | \| \| |  | Vitvi12g00484\_t001 |  |  |  |  |  |  |  |  |
| 2 | Vitvi10g04271\_t001 |  | \| \| |  | \| \| |  |  |  |  |  |  |  |  |
| 2 | Vitvi10g04272\_t001 |  | \| \| |  | \| \| |  |  |  |  |  |  |  |  |
| 2 | Vitvi10g04273\_t002 |  | \| \| |  | \| \| |  |  |  |  |  |  |  |  |
| 2 | Vitvi10g00417\_t002 |  | \| \| |  | Vitvi12g00483\_t003 |  |  |  |  |  |  |  |  |
| 2 | Vitvi10g04274\_t001 |  | \| \| |  | \| \| |  |  |  |  |  |  |  |  |
| 2 | Vitvi10g04275\_t001 |  | \| \| |  | \| \| |  |  |  |  |  |  |  |  |
| 2 | Vitvi10g01760\_t001 |  | \| \| |  | Vitvi12g04147\_t001 |  |  |  |  |  |  |  |  |
| 2 | Vitvi10g00422\_t001 |  | \| \| |  | Vitvi12g00482\_t001 |  |  |  |  |  |  |  |  |
| 2 | Vitvi10g00423\_t001 |  | \| \| |  | Vitvi12g00481\_t001 |  |  |  |  |  |  |  |  |
| 2 | Vitvi10g04276\_t001 |  | \| \| |  | \| \| |  |  |  |  |  |  |  |  |
| 2 | Vitvi10g04277\_t001 |  | \| \| |  | \| \| |  |  |  |  |  |  |  |  |
| 2 | Vitvi10g00425\_t001 |  | \| \| |  | \| \| |  |  |  |  |  |  |  |  |
| 2 | Vitvi10g00426\_t001 |  | \| \| |  | \| \| |  |  |  |  |  |  |  |  |
| 2 | Vitvi10g00427\_t001 |  | \| \| |  | \| \| |  |  |  |  |  |  |  |  |
| 2 | Vitvi10g01600\_t001 |  | \| \| |  | \| \| |  |  |  |  |  |  |  |  |
| 2 | Vitvi10g04278\_t001 |  | \| \| |  | \| \| |  |  |  |  |  |  |  |  |
| 2 | Vitvi10g00435\_t001 |  | \| \| |  | \| \| |  |  |  |  |  |  |  |  |
| 2 | Vitvi10g00436\_t001 |  | \| \| |  | \| \| |  |  |  |  |  |  |  |  |
| 2 | Vitvi10g04279\_t001 |  | \| \| |  | \| \| |  |  |  |  |  |  |  |  |
| 2 | Vitvi10g00437\_t001 |  | Vitvi19g00270\_t001 |  | \| \| |  |  |  |  |  |  |  |  |
| 2 | Vitvi10g04280\_t001 |  | \| \| |  | \| \| |  |  |  |  |  |  |  |  |
| 2 | Vitvi10g04281\_t001 |  | \| \| |  | \| \| |  |  |  |  |  |  |  |  |
| 2 | Vitvi10g04282\_t001 |  | \| \| |  | \| \| |  |  |  |  |  |  |  |  |
| 2 | Vitvi10g04283\_t001 |  | \| \| |  | \| \| |  |  |  |  |  |  |  |  |
| 2 | Vitvi10g04284\_t001 |  | \| \| |  | \| \| |  |  |  |  |  |  |  |  |
| 2 | Vitvi10g00420\_t001 |  | \| \| |  | \| \| |  |  |  |  |  |  |  |  |
| 2 | Vitvi10g02345\_t001 |  | \| \| |  | \| \| |  |  |  |  |  |  |  |  |
| 2 | Vitvi10g02346\_t001 |  | \| \| |  | \| \| |  |  |  |  |  |  |  |  |
| 2 | Vitvi10g02348\_t001 |  | \| \| |  | \| \| |  |  |  |  |  |  |  |  |
| 2 | Vitvi10g04285\_t001 |  | \| \| |  | \| \| |  |  |  |  |  |  |  |  |
| 2 | Vitvi10g02344\_t001 |  | \| \| |  | \| \| |  |  |  |  |  |  |  |  |
| 2 | Vitvi10g04286\_t002 |  | \| \| |  | \| \| |  |  |  |  |  |  |  |  |
| 2 | Vitvi10g02343\_t001 |  | \| \| |  | \| \| |  |  |  |  |  |  |  |  |
| 2 | Vitvi10g02342\_t001 |  | \| \| |  | \| \| |  |  |  |  |  |  |  |  |
| 2 | Vitvi10g02333\_t001 |  | \| \| |  | Vitvi12g00469\_t001 |  |  |  |  |  |  |  |  |
| 1 | Vitvi10g02334\_t001 |  | \| \| |  |  |  |  |  |  |  |  |  |  |
| 1 | Vitvi10g04287\_t001 |  | Vitvi19g01895\_t001 |  |  |  |  |  |  |  |  |  |  |
| 1 | Vitvi10g04288\_t001 |  | \| \| |  |  |  |  |  |  |  |  |  |  |
| 1 | Vitvi10g02336\_t001 |  | \| \| |  |  |  |  |  |  |  |  |  |  |
| 1 | Vitvi10g04289\_t001 |  | \| \| |  |  |  |  |  |  |  |  |  |  |
| 1 | Vitvi10g04290\_t001 |  | \| \| |  |  |  |  |  |  |  |  |  |  |
| 1 | Vitvi10g04291\_t001 |  | \| \| |  |  |  |  |  |  |  |  |  |  |
| 1 | Vitvi10g04292\_t001 |  | \| \| |  |  |  |  |  |  |  |  |  |  |
| 1 | Vitvi10g04293\_t001 |  | \| \| |  |  |  |  |  |  |  |  |  |  |
| 1 | Vitvi10g04294\_t002 |  | \| \| |  |  |  |  |  |  |  |  |  |  |
| 1 | Vitvi10g01762\_t001 |  | Vitvi19g00261\_t003 |  |  |  |  |  |  |  |  |  |  |
| 1 | Vitvi10g04295\_t001 |  | \| \| |  |  |  |  |  |  |  |  |  |  |
| 1 | Vitvi10g04296\_t001 |  | \| \| |  |  |  |  |  |  |  |  |  |  |
| 1 | Vitvi10g04297\_t001 |  | \| \| |  |  |  |  |  |  |  |  |  |  |
| 1 | Vitvi10g04298\_t001 |  | \| \| |  |  |  |  |  |  |  |  |  |  |
| 1 | Vitvi10g04299\_t001 |  | \| \| |  |  |  |  |  |  |  |  |  |  |
| 1 | Vitvi10g02340\_t001 |  | \| \| |  |  |  |  |  |  |  |  |  |  |
| 1 | Vitvi10g04300\_t001 |  | \| \| |  |  |  |  |  |  |  |  |  |  |
| 1 | Vitvi10g00450\_t001 |  | \| \| |  |  |  |  |  |  |  |  |  |  |
| 1 | Vitvi10g00451\_t001 |  | Vitvi19g00255\_t001 |  |  |  |  |  |  |  |  |  |  |
| 1 | Vitvi10g04301\_t001 |  | \| \| |  |  |  |  |  |  |  |  |  |  |
| 1 | Vitvi10g04302\_t001 |  | Vitvi19g00252\_t001 |  |  |  |  |  |  |  |  |  |  |
| 1 | Vitvi10g04303\_t001 |  | \| \| |  |  |  |  |  |  |  |  |  |  |
| 1 | Vitvi10g00415\_t001 |  | \| \| |  |  |  |  |  |  |  |  |  |  |
| 1 | Vitvi10g00414\_t001 |  | \| \| |  |  |  |  |  |  |  |  |  |  |
| 1 | Vitvi10g00413\_t001 |  | \| \| |  |  |  |  |  |  |  |  |  |  |
| 1 | Vitvi10g04304\_t001 |  | \| \| |  |  |  |  |  |  |  |  |  |  |
| 1 | Vitvi10g04305\_t001 |  | \| \| |  |  |  |  |  |  |  |  |  |  |
| 1 | Vitvi10g04306\_t001 |  | \| \| |  |  |  |  |  |  |  |  |  |  |
| 1 | Vitvi10g04307\_t001 |  | \| \| |  |  |  |  |  |  |  |  |  |  |
| 1 | Vitvi10g04308\_t001 |  | \| \| |  |  |  |  |  |  |  |  |  |  |
| 1 | Vitvi10g00473\_t003 |  | \| \| |  |  |  |  |  |  |  |  |  |  |
| 1 | Vitvi10g00471\_t001 |  | \| \| |  |  |  |  |  |  |  |  |  |  |
| 2 | Vitvi10g00470\_t001 |  | Vitvi19g00241\_t001 |  | Vitvi12g00322\_t001 |  |  |  |  |  |  |  |  |
| 2 | Vitvi10g00469\_t001 |  | \| \| |  | \| \| |  |  |  |  |  |  |  |  |
| 2 | Vitvi10g02375\_t001 |  | \| \| |  | \| \| |  |  |  |  |  |  |  |  |
| 2 | Vitvi10g02376\_t001 |  | \| \| |  | \| \| |  |  |  |  |  |  |  |  |
| 2 | Vitvi10g04309\_t001 |  | \| \| |  | \| \| |  |  |  |  |  |  |  |  |
| 2 | Vitvi10g00452\_t002 |  | Vitvi19g00239\_t001 |  | Vitvi12g00320\_t001 |  |  |  |  |  |  |  |  |
| 2 | Vitvi10g04310\_t001 |  | \| \| |  | \| \| |  |  |  |  |  |  |  |  |
| 2 | Vitvi10g04311\_t001 |  | \| \| |  | \| \| |  |  |  |  |  |  |  |  |
| 2 | Vitvi10g04312\_t001 |  | \| \| |  | Vitvi12g00312\_t001 |  |  |  |  |  |  |  |  |
| 2 | Vitvi10g04313\_t001 |  | \| \| |  | \| \| |  |  |  |  |  |  |  |  |
| 2 | Vitvi10g00467\_t001 |  | \| \| |  | \| \| |  |  |  |  |  |  |  |  |
| 2 | Vitvi10g04314\_t001 |  | \| \| |  | \| \| |  |  |  |  |  |  |  |  |
| 2 | Vitvi10g04315\_t001 |  | Vitvi19g00234\_t001 |  | \| \| |  |  |  |  |  |  |  |  |
| 2 | Vitvi10g00465\_t001 |  | \| \| |  | \| \| |  |  |  |  |  |  |  |  |
| 2 | Vitvi10g00462\_t002 |  | \| \| |  | Vitvi12g04069\_t001 |  |  |  |  |  |  |  |  |
| 2 | Vitvi10g04316\_t001 |  | \| \| |  | \| \| |  |  |  |  |  |  |  |  |
| 2 | Vitvi10g00461\_t001 |  | Vitvi19g01872\_t001 |  | \| \| |  |  |  |  |  |  |  |  |
| 2 | Vitvi10g01764\_t001 |  | Vitvi19g00228\_t001 |  | Vitvi12g00304\_t001 |  |  |  |  |  |  |  |  |
| 2 | Vitvi10g04317\_t001 |  | \| \| |  | \| \| |  |  |  |  |  |  |  |  |
| 2 | Vitvi10g04318\_t001 |  | \| \| |  | \| \| |  |  |  |  |  |  |  |  |
| 2 | Vitvi10g00459\_t001 |  | \| \| |  | \| \| |  |  |  |  |  |  |  |  |
| 2 | Vitvi10g04319\_t001 |  | \| \| |  | \| \| |  |  |  |  |  |  |  |  |
| 2 | Vitvi10g04320\_t001 |  | \| \| |  | \| \| |  |  |  |  |  |  |  |  |
| 2 | Vitvi10g04321\_t001 |  | \| \| |  | \| \| |  |  |  |  |  |  |  |  |
| 2 | Vitvi10g00457\_t001 |  | \| \| |  | \| \| |  |  |  |  |  |  |  |  |
| 2 | Vitvi10g04322\_t001 |  | \| \| |  | \| \| |  |  |  |  |  |  |  |  |
| 2 | Vitvi10g00455\_t001 |  | Vitvi19g00225\_t001 |  | \| \| |  |  |  |  |  |  |  |  |
| 2 | Vitvi10g00454\_t001 |  | \| \| |  | Vitvi12g00302\_t001 |  |  |  |  |  |  |  |  |
| 2 | Vitvi10g00453\_t001 |  | \| \| |  | \| \| |  |  |  |  |  |  |  |  |
| 2 | Vitvi10g04323\_t001 |  | \| \| |  | \| \| |  |  |  |  |  |  |  |  |
| 2 | Vitvi10g02253\_t001 |  | \| \| |  | Vitvi12g02307\_t001 |  |  |  |  |  |  |  |  |
| 2 | Vitvi10g02255\_t001 |  | \| \| |  | \| \| |  |  |  |  |  |  |  |  |
| 2 | Vitvi10g02256\_t001 |  | \| \| |  | \| \| |  |  |  |  |  |  |  |  |
| 2 | Vitvi10g02257\_t001 |  | Vitvi19g00209\_t001 |  | \| \| |  |  |  |  |  |  |  |  |
| 2 | Vitvi10g02258\_t001 |  | Vitvi19g01865\_t001 |  | \| \| |  |  |  |  |  |  |  |  |
| 2 | Vitvi10g04324\_t001 |  | \| \| |  | \| \| |  |  |  |  |  |  |  |  |
| 2 | Vitvi10g04325\_t001 |  | \| \| |  | \| \| |  |  |  |  |  |  |  |  |
| 2 | Vitvi10g04326\_t001 |  | \| \| |  | \| \| |  |  |  |  |  |  |  |  |
| 2 | Vitvi10g01767\_t001 |  | \| \| |  | \| \| |  |  |  |  |  |  |  |  |
| 2 | Vitvi10g00478\_t001 |  | \| \| |  | \| \| |  |  |  |  |  |  |  |  |
| 2 | Vitvi10g04327\_t001 |  | \| \| |  | \| \| |  |  |  |  |  |  |  |  |
| 2 | Vitvi10g00479\_t001 |  | \| \| |  | \| \| |  |  |  |  |  |  |  |  |
| 2 | Vitvi10g00480\_t001 |  | \| \| |  | \| \| |  |  |  |  |  |  |  |  |
| 2 | Vitvi10g04328\_t002 |  | Vitvi19g00200\_t001 |  | Vitvi12g00280\_t001 |  |  |  |  |  |  |  |  |
| 2 | Vitvi10g00482\_t001 |  | \| \| |  | \| \| |  |  |  |  |  |  |  |  |
| 2 | Vitvi10g00483\_t001 |  | \| \| |  | \| \| |  |  |  |  |  |  |  |  |
| 2 | Vitvi10g00484\_t001 |  | \| \| |  | Vitvi12g00279\_t001 |  |  |  |  |  |  |  |  |
| 2 | Vitvi10g00485\_t003 |  | \| \| |  | \| \| |  |  |  |  |  |  |  |  |
| 2 | Vitvi10g04329\_t001 |  | \| \| |  | \| \| |  |  |  |  |  |  |  |  |
| 2 | Vitvi10g00486\_t001 |  | \| \| |  | \| \| |  |  |  |  |  |  |  |  |
| 2 | Vitvi10g00487\_t001 |  | \| \| |  | \| \| |  |  |  |  |  |  |  |  |
| 2 | Vitvi10g00488\_t001 |  | Vitvi19g01784\_t001 |  | Vitvi12g00274\_t001 |  |  |  |  |  |  |  |  |
| 2 | Vitvi10g04330\_t001 |  | \| \| |  | \| \| |  |  |  |  |  |  |  |  |
| 2 | Vitvi10g01594\_t001 |  | \| \| |  | \| \| |  |  |  |  |  |  |  |  |
| 2 | Vitvi10g04331\_t001 |  | \| \| |  | \| \| |  |  |  |  |  |  |  |  |
| 2 | Vitvi10g00489\_t001 |  | \| \| |  | \| \| |  |  |  |  |  |  |  |  |
| 2 | Vitvi10g00490\_t001 |  | \| \| |  | \| \| |  |  |  |  |  |  |  |  |
| 2 | Vitvi10g04332\_t001 |  | \| \| |  | \| \| |  |  |  |  |  |  |  |  |
| 2 | Vitvi10g04333\_t001 |  | \| \| |  | \| \| |  |  |  |  |  |  |  |  |
| 2 | Vitvi10g00491\_t001 |  | Vitvi19g00184\_t001 |  | \| \| |  |  |  |  |  |  |  |  |
| 2 | Vitvi10g00492\_t001 |  | \| \| |  | Vitvi12g00268\_t001 |  |  |  |  |  |  |  |  |
| 2 | Vitvi10g01769\_t004 |  | \| \| |  | Vitvi12g00267\_t004 |  |  |  |  |  |  |  |  |
| 2 | Vitvi10g01770\_t001 |  | \| \| |  | \| \| |  |  |  |  |  |  |  |  |
| 2 | Vitvi10g01771\_t001 |  | \| \| |  | Vitvi12g00262\_t001 |  |  |  |  |  |  |  |  |
| 2 | Vitvi10g04334\_t001 |  | \| \| |  | \| \| |  |  |  |  |  |  |  |  |
| 2 | Vitvi10g01772\_t001 |  | \| \| |  | \| \| |  |  |  |  |  |  |  |  |
| 2 | Vitvi10g00494\_t001 |  | \| \| |  | \| \| |  |  |  |  |  |  |  |  |
| 2 | Vitvi10g00496\_t001 |  | \| \| |  | \| \| |  |  |  |  |  |  |  |  |
| 2 | Vitvi10g00497\_t001 |  | \| \| |  | Vitvi12g00258\_t003 |  |  |  |  |  |  |  |  |
| 2 | Vitvi10g00499\_t003 |  | \| \| |  | \| \| |  |  |  |  |  |  |  |  |
| 2 | Vitvi10g00501\_t002 |  | \| \| |  | Vitvi12g00257\_t001 |  |  |  |  |  |  |  |  |
| 2 | Vitvi10g00500\_t001 |  | \| \| |  | \| \| |  |  |  |  |  |  |  |  |
| 2 | Vitvi10g00503\_t001 |  | \| \| |  | Vitvi12g00256\_t001 |  |  |  |  |  |  |  |  |
| 2 | Vitvi10g00504\_t001 |  | \| \| |  | \| \| |  |  |  |  |  |  |  |  |
| 2 | Vitvi10g00505\_t001 |  | \| \| |  | Vitvi12g00255\_t001 |  |  |  |  |  |  |  |  |
| 2 | Vitvi10g00506\_t001 |  | \| \| |  | \| \| |  |  |  |  |  |  |  |  |
| 2 | Vitvi10g00507\_t001 |  | \| \| |  | \| \| |  |  |  |  |  |  |  |  |
| 2 | Vitvi10g01773\_t001 |  | Vitvi19g01855\_t001 |  | Vitvi12g00253\_t001 |  |  |  |  |  |  |  |  |
| 2 | Vitvi10g00508\_t001 |  | \| \| |  | Vitvi12g00251\_t001 |  |  |  |  |  |  |  |  |
| 2 | Vitvi10g00509\_t001 |  | \| \| |  | Vitvi12g00246\_t001 |  |  |  |  |  |  |  |  |
| 2 | Vitvi10g00510\_t002 |  | \| \| |  | \| \| |  |  |  |  |  |  |  |  |
| 2 | Vitvi10g00511\_t001 |  | Vitvi19g00161\_t001 |  | \| \| |  |  |  |  |  |  |  |  |
| 2 | Vitvi10g01775\_t001 |  | \| \| |  | Vitvi12g02281\_t001 |  |  |  |  |  |  |  |  |
| 2 | Vitvi10g01777\_t001 |  | \| \| |  | \| \| |  |  |  |  |  |  |  |  |
| 2 | Vitvi10g01778\_t001 |  | \| \| |  | Vitvi12g02277\_t001 |  |  |  |  |  |  |  |  |
| 2 | Vitvi10g01779\_t001 |  | \| \| |  | \| \| |  |  |  |  |  |  |  |  |
| 2 | Vitvi10g00514\_t001 |  | \| \| |  | Vitvi12g00236\_t001 |  |  |  |  |  |  |  |  |
| 2 | Vitvi10g00515\_t001 |  | \| \| |  | Vitvi12g00235\_t001 |  |  |  |  |  |  |  |  |
| 2 | Vitvi10g04335\_t001 |  | Vitvi19g01852\_t002 |  | \| \| |  |  |  |  |  |  |  |  |
| 2 | Vitvi10g00517\_t002 |  | \| \| |  | \| \| |  |  |  |  |  |  |  |  |
| 2 | Vitvi10g00518\_t001 |  | \| \| |  | \| \| |  |  |  |  |  |  |  |  |
| 2 | Vitvi10g01780\_t001 |  | \| \| |  | \| \| |  |  |  |  |  |  |  |  |
| 2 | Vitvi10g01781\_t001 |  | Vitvi19g00153\_t001 |  | Vitvi12g00233\_t001 |  |  |  |  |  |  |  |  |
| 2 | Vitvi10g00519\_t001 |  | \| \| |  | \| \| |  |  |  |  |  |  |  |  |
| 2 | Vitvi10g00520\_t001 |  | \| \| |  | Vitvi12g00231\_t001 |  |  |  |  |  |  |  |  |
| 2 | Vitvi10g00521\_t001 |  | \| \| |  | \| \| |  |  |  |  |  |  |  |  |
| 2 | Vitvi10g00522\_t001 |  | \| \| |  | \| \| |  |  |  |  |  |  |  |  |
| 2 | Vitvi10g04336\_t001 |  | \| \| |  | \| \| |  |  |  |  |  |  |  |  |
| 2 | Vitvi10g00523\_t001 |  | \| \| |  | \| \| |  |  |  |  |  |  |  |  |
| 2 | Vitvi10g00524\_t001 |  | \| \| |  | \| \| |  |  |  |  |  |  |  |  |
| 2 | Vitvi10g00525\_t001 |  | \| \| |  | \| \| |  |  |  |  |  |  |  |  |
| 2 | Vitvi10g00526\_t001 |  | \| \| |  | \| \| |  |  |  |  |  |  |  |  |
| 2 | Vitvi10g00527\_t001 |  | \| \| |  | \| \| |  |  |  |  |  |  |  |  |
| 2 | Vitvi10g04337\_t001 |  | \| \| |  | \| \| |  |  |  |  |  |  |  |  |
| 2 | Vitvi10g00528\_t001 |  | \| \| |  | \| \| |  |  |  |  |  |  |  |  |
| 2 | Vitvi10g00529\_t001 |  | Vitvi19g00148\_t001 |  | \| \| |  |  |  |  |  |  |  |  |
| 2 | Vitvi10g04338\_t001 |  | \| \| |  | \| \| |  |  |  |  |  |  |  |  |
| 2 | Vitvi10g04339\_t001 |  | \| \| |  | \| \| |  |  |  |  |  |  |  |  |
| 2 | Vitvi10g00531\_t001 |  | \| \| |  | \| \| |  |  |  |  |  |  |  |  |
| 2 | Vitvi10g01784\_t001 |  | \| \| |  | \| \| |  |  |  |  |  |  |  |  |
| 2 | Vitvi10g04340\_t001 |  | \| \| |  | \| \| |  |  |  |  |  |  |  |  |
| 2 | Vitvi10g04341\_t001 |  | \| \| |  | \| \| |  |  |  |  |  |  |  |  |
| 2 | Vitvi10g04342\_t001 |  | \| \| |  | \| \| |  |  |  |  |  |  |  |  |
| 2 | Vitvi10g04343\_t001 |  | \| \| |  | \| \| |  |  |  |  |  |  |  |  |
| 2 | Vitvi10g04344\_t001 |  | \| \| |  | \| \| |  |  |  |  |  |  |  |  |
| 2 | Vitvi10g01787\_t001 |  | Vitvi19g01847\_t001 |  | \| \| |  |  |  |  |  |  |  |  |
| 2 | Vitvi10g00535\_t001 |  | \| \| |  | Vitvi12g00225\_t002 |  |  |  |  |  |  |  |  |
| 2 | Vitvi10g00536\_t001 |  | \| \| |  | \| \| |  |  |  |  |  |  |  |  |
| 2 | Vitvi10g00537\_t001 |  | Vitvi19g00143\_t001 |  | Vitvi12g02265\_t001 |  |  |  |  |  |  |  |  |
| 2 | Vitvi10g04345\_t001 |  | Vitvi19g01844\_t001 |  | \| \| |  |  |  |  |  |  |  |  |
| 2 | Vitvi10g04346\_t001 |  | \| \| |  | \| \| |  |  |  |  |  |  |  |  |
| 2 | Vitvi10g01788\_t001 |  | \| \| |  | Vitvi12g00220\_t001 |  |  |  |  |  |  |  |  |
| 2 | Vitvi10g00539\_t001 |  | Vitvi19g00141\_t001 |  | \| \| |  |  |  |  |  |  |  |  |
| 2 | Vitvi10g00540\_t001 |  | Vitvi19g00140\_t001 |  | Vitvi12g00219\_t001 |  |  |  |  |  |  |  |  |
| 2 | Vitvi10g04347\_t001 |  | \| \| |  | \| \| |  |  |  |  |  |  |  |  |
| 2 | Vitvi10g00541\_t001 |  | \| \| |  | \| \| |  |  |  |  |  |  |  |  |
| 2 | Vitvi10g00542\_t001 |  | \| \| |  | \| \| |  |  |  |  |  |  |  |  |
| 2 | Vitvi10g00543\_t001 |  | \| \| |  | \| \| |  |  |  |  |  |  |  |  |
| 2 | Vitvi10g00544\_t001 |  | \| \| |  | \| \| |  |  |  |  |  |  |  |  |
| 2 | Vitvi10g01789\_t001 |  | \| \| |  | \| \| |  |  |  |  |  |  |  |  |
| 2 | Vitvi10g04348\_t001 |  | \| \| |  | Vitvi12g04054\_t001 |  |  |  |  |  |  |  |  |
| 2 | Vitvi10g04349\_t001 |  | \| \| |  | \| \| |  |  |  |  |  |  |  |  |
| 2 | Vitvi10g04350\_t001 |  | \| \| |  | \| \| |  |  |  |  |  |  |  |  |
| 2 | Vitvi10g04351\_t001 |  | \| \| |  | \| \| |  |  |  |  |  |  |  |  |
| 2 | Vitvi10g04352\_t001 |  | \| \| |  | \| \| |  |  |  |  |  |  |  |  |
| 2 | Vitvi10g04353\_t001 |  | \| \| |  | \| \| |  |  |  |  |  |  |  |  |
| 2 | Vitvi10g04354\_t001 |  | \| \| |  | \| \| |  |  |  |  |  |  |  |  |
| 2 | Vitvi10g04355\_t001 |  | \| \| |  | \| \| |  |  |  |  |  |  |  |  |
| 2 | Vitvi10g04356\_t001 |  | \| \| |  | \| \| |  |  |  |  |  |  |  |  |
| 2 | Vitvi10g04357\_t001 |  | \| \| |  | \| \| |  |  |  |  |  |  |  |  |
| 2 | Vitvi10g00551\_t002 |  | \| \| |  | \| \| |  |  |  |  |  |  |  |  |
| 2 | Vitvi10g00552\_t001 |  | \| \| |  | \| \| |  |  |  |  |  |  |  |  |
| 2 | Vitvi10g01790\_t001 |  | \| \| |  | Vitvi12g02262\_t001 |  |  |  |  |  |  |  |  |
| 2 | Vitvi10g00553\_t001 |  | \| \| |  | \| \| |  |  |  |  |  |  |  |  |
| 2 | Vitvi10g00554\_t001 |  | Vitvi19g00138\_t001 |  | \| \| |  |  |  |  |  |  |  |  |
| 2 | Vitvi10g00555\_t001 |  | \| \| |  | Vitvi12g00213\_t001 |  |  |  |  |  |  |  |  |
| 2 | Vitvi10g00557\_t001 |  | \| \| |  | \| \| |  |  |  |  |  |  |  |  |
| 2 | Vitvi10g04358\_t001 |  | \| \| |  | \| \| |  |  |  |  |  |  |  |  |
| 2 | Vitvi10g00558\_t001 |  | \| \| |  | \| \| |  |  |  |  |  |  |  |  |
| 2 | Vitvi10g00559\_t001 |  | \| \| |  | \| \| |  |  |  |  |  |  |  |  |
| 2 | Vitvi10g00561\_t001 |  | \| \| |  | Vitvi12g00212\_t001 |  |  |  |  |  |  |  |  |
| 2 | Vitvi10g00562\_t001 |  | \| \| |  | \| \| |  |  |  |  |  |  |  |  |
| 2 | Vitvi10g00563\_t001 |  | \| \| |  | \| \| |  |  |  |  |  |  |  |  |
| 2 | Vitvi10g00564\_t001 |  | \| \| |  | Vitvi12g00209\_t001 |  |  |  |  |  |  |  |  |
| 2 | Vitvi10g00565\_t001 |  | \| \| |  | \| \| |  |  |  |  |  |  |  |  |
| 2 | Vitvi10g04359\_t001 |  | \| \| |  | \| \| |  |  |  |  |  |  |  |  |
| 2 | Vitvi10g00566\_t001 |  | \| \| |  | \| \| |  |  |  |  |  |  |  |  |
| 2 | Vitvi10g00567\_t001 |  | \| \| |  | \| \| |  |  |  |  |  |  |  |  |
| 2 | Vitvi10g00568\_t001 |  | \| \| |  | \| \| |  |  |  |  |  |  |  |  |
| 2 | Vitvi10g01791\_t002 |  | \| \| |  | \| \| |  |  |  |  |  |  |  |  |
| 2 | Vitvi10g04360\_t001 |  | Vitvi19g00131\_t001 |  | \| \| |  |  |  |  |  |  |  |  |
| 2 | Vitvi10g00570\_t001 |  | \| \| |  | \| \| |  |  |  |  |  |  |  |  |
| 2 | Vitvi10g00571\_t001 |  | \| \| |  | \| \| |  |  |  |  |  |  |  |  |
| 2 | Vitvi10g00572\_t001 |  | \| \| |  | Vitvi12g00200\_t001 |  |  |  |  |  |  |  |  |
| 2 | Vitvi10g00573\_t001 |  | \| \| |  | Vitvi12g00198\_t001 |  |  |  |  |  |  |  |  |
| 2 | Vitvi10g01792\_t001 |  | \| \| |  | \| \| |  |  |  |  |  |  |  |  |
| 2 | Vitvi10g00574\_t001 |  | \| \| |  | \| \| |  |  |  |  |  |  |  |  |
| 2 | Vitvi10g00575\_t001 |  | \| \| |  | Vitvi12g02253\_t001 |  |  |  |  |  |  |  |  |
| 2 | Vitvi10g00576\_t001 |  | \| \| |  | \| \| |  |  |  |  |  |  |  |  |
| 2 | Vitvi10g00577\_t001 |  | \| \| |  | \| \| |  |  |  |  |  |  |  |  |
| 2 | Vitvi10g04361\_t001 |  | \| \| |  | \| \| |  |  |  |  |  |  |  |  |
| 2 | Vitvi10g00579\_t003 |  | Vitvi19g00115\_t001 |  | Vitvi12g00187\_t001 |  |  |  |  |  |  |  |  |
| 2 | Vitvi10g00580\_t001 |  | \| \| |  | \| \| |  |  |  |  |  |  |  |  |
| 2 | Vitvi10g00581\_t001 |  | \| \| |  | \| \| |  |  |  |  |  |  |  |  |
| 2 | Vitvi10g00582\_t001 |  | \| \| |  | \| \| |  |  |  |  |  |  |  |  |
| 2 | Vitvi10g00583\_t001 |  | Vitvi19g00108\_t001 |  | Vitvi12g00183\_t001 |  |  |  |  |  |  |  |  |
| 2 | Vitvi10g00584\_t002 |  | \| \| |  | Vitvi12g00182\_t001 |  |  |  |  |  |  |  |  |
| 2 | Vitvi10g00585\_t001 |  | \| \| |  | \| \| |  |  |  |  |  |  |  |  |
| 2 | Vitvi10g00586\_t001 |  | \| \| |  | \| \| |  |  |  |  |  |  |  |  |
| 2 | Vitvi10g00587\_t001 |  | \| \| |  | \| \| |  |  |  |  |  |  |  |  |
| 2 | Vitvi10g04362\_t001 |  | \| \| |  | \| \| |  |  |  |  |  |  |  |  |
| 2 | Vitvi10g04363\_t001 |  | \| \| |  | \| \| |  |  |  |  |  |  |  |  |
| 2 | Vitvi10g04364\_t001 |  | \| \| |  | Vitvi12g02250\_t001 |  |  |  |  |  |  |  |  |
| 2 | Vitvi10g00591\_t001 |  | \| \| |  | \| \| |  |  |  |  |  |  |  |  |
| 2 | Vitvi10g00592\_t001 |  | \| \| |  | Vitvi12g00179\_t001 |  |  |  |  |  |  |  |  |
| 2 | Vitvi10g01793\_t001 |  | \| \| |  | Vitvi12g00177\_t001 |  |  |  |  |  |  |  |  |
| 2 | Vitvi10g00593\_t001 |  | \| \| |  | Vitvi12g00175\_t001 |  |  |  |  |  |  |  |  |
| 2 | Vitvi10g04365\_t001 |  | \| \| |  | \| \| |  |  |  |  |  |  |  |  |
| 2 | Vitvi10g04366\_t001 |  | \| \| |  | \| \| |  |  |  |  |  |  |  |  |
| 2 | Vitvi10g04367\_t001 |  | \| \| |  | \| \| |  |  |  |  |  |  |  |  |
| 2 | Vitvi10g04368\_t001 |  | \| \| |  | \| \| |  |  |  |  |  |  |  |  |
| 2 | Vitvi10g00597\_t001 |  | Vitvi19g00098\_t001 |  | \| \| |  |  |  |  |  |  |  |  |
| 2 | Vitvi10g00598\_t001 |  | Vitvi19g01837\_t001 |  | \| \| |  |  |  |  |  |  |  |  |
| 2 | Vitvi10g00599\_t001 |  | \| \| |  | \| \| |  |  |  |  |  |  |  |  |
| 2 | Vitvi10g00600\_t001 |  | \| \| |  | \| \| |  |  |  |  |  |  |  |  |
| 2 | Vitvi10g00601\_t002 |  | \| \| |  | \| \| |  |  |  |  |  |  |  |  |
| 2 | Vitvi10g01794\_t004 |  | \| \| |  | \| \| |  |  |  |  |  |  |  |  |
| 2 | Vitvi10g00602\_t001 |  | \| \| |  | \| \| |  |  |  |  |  |  |  |  |
| 2 | Vitvi10g00603\_t001 |  | \| \| |  | \| \| |  |  |  |  |  |  |  |  |
| 2 | Vitvi10g00604\_t001 |  | \| \| |  | Vitvi12g00157\_t001 |  |  |  |  |  |  |  |  |
| 2 | Vitvi10g00605\_t001 |  | \| \| |  | \| \| |  |  |  |  |  |  |  |  |
| 2 | Vitvi10g04369\_t001 |  | \| \| |  | \| \| |  |  |  |  |  |  |  |  |
| 2 | Vitvi10g00607\_t001 |  | \| \| |  | \| \| |  |  |  |  |  |  |  |  |
| 2 | Vitvi10g04370\_t001 |  | \| \| |  | \| \| |  |  |  |  |  |  |  |  |
| 2 | Vitvi10g00608\_t001 |  | \| \| |  | \| \| |  |  |  |  |  |  |  |  |
| 2 | Vitvi10g00609\_t001 |  | \| \| |  | Vitvi12g00155\_t001 |  |  |  |  |  |  |  |  |
| 2 | Vitvi10g01796\_t001 |  | \| \| |  | \| \| |  |  |  |  |  |  |  |  |
| 2 | Vitvi10g00611\_t001 |  | \| \| |  | \| \| |  |  |  |  |  |  |  |  |
| 2 | Vitvi10g00612\_t001 |  | \| \| |  | \| \| |  |  |  |  |  |  |  |  |
| 2 | Vitvi10g00613\_t001 |  | \| \| |  | \| \| |  |  |  |  |  |  |  |  |
| 2 | Vitvi10g00614\_t001 |  | \| \| |  | \| \| |  |  |  |  |  |  |  |  |
| 2 | Vitvi10g04371\_t001 |  | \| \| |  | \| \| |  |  |  |  |  |  |  |  |
| 2 | Vitvi10g00615\_t001 |  | \| \| |  | Vitvi12g02233\_t001 |  |  |  |  |  |  |  |  |
| 2 | Vitvi10g00617\_t001 |  | \| \| |  | Vitvi12g00149\_t001 |  |  |  |  |  |  |  |  |
| 2 | Vitvi10g00618\_t001 |  | \| \| |  | Vitvi12g00148\_t001 |  |  |  |  |  |  |  |  |
| 2 | Vitvi10g00619\_t001 |  | Vitvi19g00091\_t001 |  | \| \| |  |  |  |  |  |  |  |  |
| 2 | Vitvi10g04372\_t001 |  | Vitvi19g01833\_t001 |  | \| \| |  |  |  |  |  |  |  |  |
| 2 | Vitvi10g04373\_t001 |  | \| \| |  | \| \| |  |  |  |  |  |  |  |  |
| 2 | Vitvi10g00622\_t001 |  | \| \| |  | \| \| |  |  |  |  |  |  |  |  |
| 2 | Vitvi10g01798\_t001 |  | Vitvi19g04048\_t001 |  | \| \| |  |  |  |  |  |  |  |  |
| 2 | Vitvi10g04374\_t001 |  | \| \| |  | \| \| |  |  |  |  |  |  |  |  |
| 2 | Vitvi10g01799\_t001 |  | \| \| |  | \| \| |  |  |  |  |  |  |  |  |
| 2 | Vitvi10g04375\_t001 |  | \| \| |  | \| \| |  |  |  |  |  |  |  |  |
| 2 | Vitvi10g00624\_t001 |  | \| \| |  | \| \| |  |  |  |  |  |  |  |  |
| 2 | Vitvi10g04376\_t001 |  | \| \| |  | \| \| |  |  |  |  |  |  |  |  |
| 2 | Vitvi10g00625\_t001 |  | \| \| |  | Vitvi12g00146\_t001 |  |  |  |  |  |  |  |  |
| 2 | Vitvi10g04377\_t001 |  | \| \| |  | \| \| |  |  |  |  |  |  |  |  |
| 2 | Vitvi10g00626\_t001 |  | Vitvi19g01830\_t002 |  | \| \| |  |  |  |  |  |  |  |  |
| 2 | Vitvi10g00627\_t001 |  | \| \| |  | \| \| |  |  |  |  |  |  |  |  |
| 2 | Vitvi10g00628\_t001 |  | Vitvi19g01829\_t001 |  | \| \| |  |  |  |  |  |  |  |  |
| 2 | Vitvi10g04378\_t001 |  | \| \| |  | \| \| |  |  |  |  |  |  |  |  |
| 2 | Vitvi10g00629\_t001 |  | \| \| |  | \| \| |  |  |  |  |  |  |  |  |
| 2 | Vitvi10g00630\_t001 |  | \| \| |  | Vitvi12g04041\_t001 |  |  |  |  |  |  |  |  |
| 1 | Vitvi10g00631\_t001 |  | \| \| |  |  |  |  |  |  |  |  |  |  |
| 1 | Vitvi10g00632\_t001 |  | \| \| |  |  |  |  |  |  |  |  |  |  |
| 1 | Vitvi10g00634\_t001 |  | \| \| |  |  |  |  |  |  |  |  |  |  |
| 1 | Vitvi10g00635\_t002 |  | \| \| |  |  |  |  |  |  |  |  |  |  |
| 1 | Vitvi10g00636\_t001 |  | Vitvi19g00061\_t001 |  |  |  |  |  |  |  |  |  |  |
| 1 | Vitvi10g00637\_t002 |  | Vitvi19g00060\_t001 |  |  |  |  |  |  |  |  |  |  |
| 1 | Vitvi10g04379\_t001 |  | \| \| |  |  |  |  |  |  |  |  |  |  |
| 1 | Vitvi10g00638\_t001 |  | \| \| |  |  |  |  |  |  |  |  |  |  |
| 1 | Vitvi10g00639\_t001 |  | \| \| |  |  |  |  |  |  |  |  |  |  |
| 1 | Vitvi10g00640\_t001 |  | Vitvi19g00058\_t001 |  |  |  |  |  |  |  |  |  |  |
| 1 | Vitvi10g00641\_t001 |  | \| \| |  |  |  |  |  |  |  |  |  |  |
| 1 | Vitvi10g01592\_t001 |  | \| \| |  |  |  |  |  |  |  |  |  |  |
| 1 | Vitvi10g04380\_t001 |  | \| \| |  |  |  |  |  |  |  |  |  |  |
| 1 | Vitvi10g00643\_t001 |  | \| \| |  |  |  |  |  |  |  |  |  |  |
| 1 | Vitvi10g01804\_t001 |  | \| \| |  |  |  |  |  |  |  |  |  |  |
| 1 | Vitvi10g00644\_t001 |  | \| \| |  |  |  |  |  |  |  |  |  |  |
| 1 | Vitvi10g00647\_t001 |  | \| \| |  |  |  |  |  |  |  |  |  |  |
| 1 | Vitvi10g04381\_t001 |  | \| \| |  |  |  |  |  |  |  |  |  |  |
| 1 | Vitvi10g04382\_t001 |  | Vitvi19g04027\_t001 |  |  |  |  |  |  |  |  |  |  |
| 0 | Vitvi10g04383\_t001 |  |  |  |  |  |  |  |  |  |  |  |  |
| 0 | Vitvi10g04384\_t001 |  |  |  |  |  |  |  |  |  |  |  |  |
| 0 | Vitvi10g00651\_t001 |  |  |  |  |  |  |  |  |  |  |  |  |
| 0 | Vitvi10g00652\_t001 |  |  |  |  |  |  |  |  |  |  |  |  |
| 0 | Vitvi10g00655\_t001 |  |  |  |  |  |  |  |  |  |  |  |  |
| 0 | Vitvi10g00656\_t002 |  |  |  |  |  |  |  |  |  |  |  |  |
| 0 | Vitvi10g00657\_t001 |  |  |  |  |  |  |  |  |  |  |  |  |
| 0 | Vitvi10g00659\_t001 |  |  |  |  |  |  |  |  |  |  |  |  |
| 0 | Vitvi10g00660\_t001 |  |  |  |  |  |  |  |  |  |  |  |  |
| 0 | Vitvi10g00661\_t002 |  |  |  |  |  |  |  |  |  |  |  |  |
| 0 | Vitvi10g00662\_t001 |  |  |  |  |  |  |  |  |  |  |  |  |
| 0 | Vitvi10g00663\_t001 |  |  |  |  |  |  |  |  |  |  |  |  |
| 0 | Vitvi10g00665\_t001 |  |  |  |  |  |  |  |  |  |  |  |  |
| 0 | Vitvi10g00666\_t001 |  |  |  |  |  |  |  |  |  |  |  |  |
| 0 | Vitvi10g04385\_t001 |  |  |  |  |  |  |  |  |  |  |  |  |
| 0 | Vitvi10g00667\_t001 |  |  |  |  |  |  |  |  |  |  |  |  |
| 0 | Vitvi10g00668\_t002 |  |  |  |  |  |  |  |  |  |  |  |  |
| 0 | Vitvi10g00669\_t001 |  |  |  |  |  |  |  |  |  |  |  |  |
| 0 | Vitvi10g04386\_t001 |  |  |  |  |  |  |  |  |  |  |  |  |
| 0 | Vitvi10g01811\_t001 |  |  |  |  |  |  |  |  |  |  |  |  |
| 0 | Vitvi10g04387\_t001 |  |  |  |  |  |  |  |  |  |  |  |  |
| 0 | Vitvi10g04388\_t001 |  |  |  |  |  |  |  |  |  |  |  |  |
| 0 | Vitvi10g01812\_t001 |  |  |  |  |  |  |  |  |  |  |  |  |
| 0 | Vitvi10g01813\_t001 |  |  |  |  |  |  |  |  |  |  |  |  |
| 0 | Vitvi10g00678\_t001 |  |  |  |  |  |  |  |  |  |  |  |  |
| 0 | Vitvi10g00679\_t001 |  |  |  |  |  |  |  |  |  |  |  |  |
| 0 | Vitvi10g00680\_t001 |  |  |  |  |  |  |  |  |  |  |  |  |
| 0 | Vitvi10g00681\_t001 |  |  |  |  |  |  |  |  |  |  |  |  |
| 0 | Vitvi10g01814\_t001 |  |  |  |  |  |  |  |  |  |  |  |  |
| 0 | Vitvi10g04389\_t001 |  |  |  |  |  |  |  |  |  |  |  |  |
| 0 | Vitvi10g00684\_t001 |  |  |  |  |  |  |  |  |  |  |  |  |
| 0 | Vitvi10g00685\_t001 |  |  |  |  |  |  |  |  |  |  |  |  |
| 0 | Vitvi10g00687\_t001 |  |  |  |  |  |  |  |  |  |  |  |  |
| 0 | Vitvi10g04390\_t001 |  |  |  |  |  |  |  |  |  |  |  |  |
| 0 | Vitvi10g04391\_t001 |  |  |  |  |  |  |  |  |  |  |  |  |
| 0 | Vitvi10g01817\_t001 |  |  |  |  |  |  |  |  |  |  |  |  |
| 0 | Vitvi10g04392\_t001 |  |  |  |  |  |  |  |  |  |  |  |  |
| 0 | Vitvi10g04393\_t001 |  |  |  |  |  |  |  |  |  |  |  |  |
| 0 | Vitvi10g01821\_t001 |  |  |  |  |  |  |  |  |  |  |  |  |
| 0 | Vitvi10g01822\_t001 |  |  |  |  |  |  |  |  |  |  |  |  |
| 0 | Vitvi10g04394\_t001 |  |  |  |  |  |  |  |  |  |  |  |  |
| 0 | Vitvi10g04395\_t001 |  |  |  |  |  |  |  |  |  |  |  |  |
| 0 | Vitvi10g01823\_t001 |  |  |  |  |  |  |  |  |  |  |  |  |
| 0 | Vitvi10g01824\_t001 |  |  |  |  |  |  |  |  |  |  |  |  |
| 0 | Vitvi10g04396\_t001 |  |  |  |  |  |  |  |  |  |  |  |  |
| 0 | Vitvi10g04397\_t001 |  |  |  |  |  |  |  |  |  |  |  |  |
| 0 | Vitvi10g00689\_t001 |  |  |  |  |  |  |  |  |  |  |  |  |
| 0 | Vitvi10g04398\_t001 |  |  |  |  |  |  |  |  |  |  |  |  |
| 0 | Vitvi10g01828\_t001 |  |  |  |  |  |  |  |  |  |  |  |  |
| 0 | Vitvi10g01829\_t001 |  |  |  |  |  |  |  |  |  |  |  |  |
| 0 | Vitvi10g01831\_t001 |  |  |  |  |  |  |  |  |  |  |  |  |
| 0 | Vitvi10g00693\_t001 |  |  |  |  |  |  |  |  |  |  |  |  |
| 0 | Vitvi10g04399\_t001 |  |  |  |  |  |  |  |  |  |  |  |  |
| 0 | Vitvi10g00694\_t001 |  |  |  |  |  |  |  |  |  |  |  |  |
| 0 | Vitvi10g01832\_t001 |  |  |  |  |  |  |  |  |  |  |  |  |
| 0 | Vitvi10g00697\_t001 |  |  |  |  |  |  |  |  |  |  |  |  |
| 0 | Vitvi10g01833\_t001 |  |  |  |  |  |  |  |  |  |  |  |  |
| 0 | Vitvi10g04400\_t001 |  |  |  |  |  |  |  |  |  |  |  |  |
| 0 | Vitvi10g04401\_t001 |  |  |  |  |  |  |  |  |  |  |  |  |
| 0 | Vitvi10g04402\_t001 |  |  |  |  |  |  |  |  |  |  |  |  |
| 0 | Vitvi10g04403\_t001 |  |  |  |  |  |  |  |  |  |  |  |  |
| 0 | Vitvi10g04404\_t001 |  |  |  |  |  |  |  |  |  |  |  |  |
| 0 | Vitvi10g00702\_t001 |  |  |  |  |  |  |  |  |  |  |  |  |
| 0 | Vitvi10g04405\_t001 |  |  |  |  |  |  |  |  |  |  |  |  |
| 0 | Vitvi10g04406\_t001 |  |  |  |  |  |  |  |  |  |  |  |  |
| 0 | Vitvi10g00703\_t001 |  |  |  |  |  |  |  |  |  |  |  |  |
| 0 | Vitvi10g00705\_t001 |  |  |  |  |  |  |  |  |  |  |  |  |
| 0 | Vitvi10g00706\_t001 |  |  |  |  |  |  |  |  |  |  |  |  |
| 0 | Vitvi10g00707\_t001 |  |  |  |  |  |  |  |  |  |  |  |  |
| 0 | Vitvi10g00708\_t001 |  |  |  |  |  |  |  |  |  |  |  |  |
| 0 | Vitvi10g00709\_t001 |  |  |  |  |  |  |  |  |  |  |  |  |
| 0 | Vitvi10g01835\_t001 |  |  |  |  |  |  |  |  |  |  |  |  |
| 0 | Vitvi10g04407\_t001 |  |  |  |  |  |  |  |  |  |  |  |  |
| 0 | Vitvi10g00711\_t001 |  |  |  |  |  |  |  |  |  |  |  |  |
| 0 | Vitvi10g00712\_t001 |  |  |  |  |  |  |  |  |  |  |  |  |
| 0 | Vitvi10g00713\_t001 |  |  |  |  |  |  |  |  |  |  |  |  |
| 1 | Vitvi10g00714\_t001 |  | Vitvi12g00033\_t001 |  |  |  |  |  |  |  |  |  |  |
| 1 | Vitvi10g01589\_t001 |  | \| \| |  |  |  |  |  |  |  |  |  |  |
| 1 | Vitvi10g00715\_t001 |  | \| \| |  |  |  |  |  |  |  |  |  |  |
| 1 | Vitvi10g04408\_t001 |  | \| \| |  |  |  |  |  |  |  |  |  |  |
| 1 | Vitvi10g00716\_t001 |  | \| \| |  |  |  |  |  |  |  |  |  |  |
| 1 | Vitvi10g00717\_t001 |  | \| \| |  |  |  |  |  |  |  |  |  |  |
| 1 | Vitvi10g00719\_t001 |  | \| \| |  |  |  |  |  |  |  |  |  |  |
| 1 | Vitvi10g00720\_t001 |  | Vitvi12g00038\_t001 |  |  |  |  |  |  |  |  |  |  |
| 1 | Vitvi10g01837\_t001 |  | \| \| |  |  |  |  |  |  |  |  |  |  |
| 1 | Vitvi10g00722\_t001 |  | Vitvi12g02191\_t001 |  |  |  |  |  |  |  |  |  |  |
| 1 | Vitvi10g00723\_t004 |  | \| \| |  |  |  |  |  |  |  |  |  |  |
| 1 | Vitvi10g00724\_t001 |  | \| \| |  |  |  |  |  |  |  |  |  |  |
| 1 | Vitvi10g00726\_t001 |  | \| \| |  |  |  |  |  |  |  |  |  |  |
| 1 | Vitvi10g00727\_t001 |  | \| \| |  |  |  |  |  |  |  |  |  |  |
| 1 | Vitvi10g00728\_t001 |  | \| \| |  |  |  |  |  |  |  |  |  |  |
| 1 | Vitvi10g04409\_t001 |  | Vitvi12g02192\_t001 |  |  |  |  |  |  |  |  |  |  |
| 1 | Vitvi10g00732\_t001 |  | Vitvi12g00048\_t001 |  |  |  |  |  |  |  |  |  |  |
| 1 | Vitvi10g00733\_t001 |  | \| \| |  |  |  |  |  |  |  |  |  |  |
| 1 | Vitvi10g00734\_t001 |  | \| \| |  |  |  |  |  |  |  |  |  |  |
| 1 | Vitvi10g04410\_t001 |  | \| \| |  |  |  |  |  |  |  |  |  |  |
| 1 | Vitvi10g00736\_t001 |  | \| \| |  |  |  |  |  |  |  |  |  |  |
| 1 | Vitvi10g00737\_t001 |  | \| \| |  |  |  |  |  |  |  |  |  |  |
| 1 | Vitvi10g00738\_t001 |  | \| \| |  |  |  |  |  |  |  |  |  |  |
| 1 | Vitvi10g01838\_t001 |  | \| \| |  |  |  |  |  |  |  |  |  |  |
| 1 | Vitvi10g00739\_t001 |  | \| \| |  |  |  |  |  |  |  |  |  |  |
| 1 | Vitvi10g01839\_t001 |  | Vitvi12g00050\_t001 |  |  |  |  |  |  |  |  |  |  |
| 0 | Vitvi10g00740\_t001 |  |  |  |  |  |  |  |  |  |  |  |  |
| 0 | Vitvi10g00741\_t001 |  |  |  |  |  |  |  |  |  |  |  |  |
| 0 | Vitvi10g00742\_t001 |  |  |  |  |  |  |  |  |  |  |  |  |
| 0 | Vitvi10g04411\_t001 |  |  |  |  |  |  |  |  |  |  |  |  |
| 0 | Vitvi10g04412\_t001 |  |  |  |  |  |  |  |  |  |  |  |  |
| 0 | Vitvi10g04413\_t001 |  |  |  |  |  |  |  |  |  |  |  |  |
| 0 | Vitvi10g00744\_t001 |  |  |  |  |  |  |  |  |  |  |  |  |
| 0 | Vitvi10g04414\_t001 |  |  |  |  |  |  |  |  |  |  |  |  |
| 0 | Vitvi10g00746\_t001 |  |  |  |  |  |  |  |  |  |  |  |  |
| 0 | Vitvi10g00747\_t001 |  |  |  |  |  |  |  |  |  |  |  |  |
| 0 | Vitvi10g00748\_t001 |  |  |  |  |  |  |  |  |  |  |  |  |
| 0 | Vitvi10g00749\_t001 |  |  |  |  |  |  |  |  |  |  |  |  |
| 0 | Vitvi10g01841\_t001 |  |  |  |  |  |  |  |  |  |  |  |  |
| 0 | Vitvi10g01588\_t001 |  |  |  |  |  |  |  |  |  |  |  |  |
| 0 | Vitvi10g01593\_t001 |  |  |  |  |  |  |  |  |  |  |  |  |
| 0 | Vitvi10g01591\_t001 |  |  |  |  |  |  |  |  |  |  |  |  |
| 0 | Vitvi10g01590\_t001 |  |  |  |  |  |  |  |  |  |  |  |  |
| 0 | Vitvi10g04415\_t001 |  |  |  |  |  |  |  |  |  |  |  |  |
| 0 | Vitvi10g00751\_t001 |  |  |  |  |  |  |  |  |  |  |  |  |
| 0 | Vitvi10g00752\_t001 |  |  |  |  |  |  |  |  |  |  |  |  |
| 0 | Vitvi10g01844\_t001 |  |  |  |  |  |  |  |  |  |  |  |  |
| 0 | Vitvi10g00753\_t001 |  |  |  |  |  |  |  |  |  |  |  |  |
| 0 | Vitvi10g00754\_t001 |  |  |  |  |  |  |  |  |  |  |  |  |
| 0 | Vitvi10g00755\_t001 |  |  |  |  |  |  |  |  |  |  |  |  |
| 0 | Vitvi10g04416\_t001 |  |  |  |  |  |  |  |  |  |  |  |  |
| 0 | Vitvi10g00756\_t001 |  |  |  |  |  |  |  |  |  |  |  |  |
| 0 | Vitvi10g00757\_t001 |  |  |  |  |  |  |  |  |  |  |  |  |
| 0 | Vitvi10g00759\_t001 |  |  |  |  |  |  |  |  |  |  |  |  |
| 0 | Vitvi10g00761\_t003 |  |  |  |  |  |  |  |  |  |  |  |  |
| 0 | Vitvi10g04417\_t001 |  |  |  |  |  |  |  |  |  |  |  |  |
| 0 | Vitvi10g00764\_t001 |  |  |  |  |  |  |  |  |  |  |  |  |
| 0 | Vitvi10g04418\_t001 |  |  |  |  |  |  |  |  |  |  |  |  |
| 0 | Vitvi10g00765\_t001 |  |  |  |  |  |  |  |  |  |  |  |  |
| 0 | Vitvi10g00767\_t002 |  |  |  |  |  |  |  |  |  |  |  |  |
| 0 | Vitvi10g04419\_t001 |  |  |  |  |  |  |  |  |  |  |  |  |
| 0 | Vitvi10g00769\_t001 |  |  |  |  |  |  |  |  |  |  |  |  |
| 0 | Vitvi10g04420\_t001 |  |  |  |  |  |  |  |  |  |  |  |  |
| 0 | Vitvi10g01848\_t001 |  |  |  |  |  |  |  |  |  |  |  |  |
| 0 | Vitvi10g00774\_t001 |  |  |  |  |  |  |  |  |  |  |  |  |
| 0 | Vitvi10g00775\_t001 |  |  |  |  |  |  |  |  |  |  |  |  |
| 0 | Vitvi10g00777\_t001 |  |  |  |  |  |  |  |  |  |  |  |  |
| 0 | Vitvi10g00778\_t001 |  |  |  |  |  |  |  |  |  |  |  |  |
| 0 | Vitvi10g01850\_t001 |  |  |  |  |  |  |  |  |  |  |  |  |
| 0 | Vitvi10g00780\_t001 |  |  |  |  |  |  |  |  |  |  |  |  |
| 0 | Vitvi10g00781\_t001 |  |  |  |  |  |  |  |  |  |  |  |  |
| 0 | Vitvi10g00782\_t001 |  |  |  |  |  |  |  |  |  |  |  |  |
| 0 | Vitvi10g00783\_t001 |  |  |  |  |  |  |  |  |  |  |  |  |
| 0 | Vitvi10g01851\_t001 |  |  |  |  |  |  |  |  |  |  |  |  |
| 0 | Vitvi10g04421\_t001 |  |  |  |  |  |  |  |  |  |  |  |  |
| 0 | Vitvi10g01852\_t001 |  |  |  |  |  |  |  |  |  |  |  |  |
| 0 | Vitvi10g01854\_t001 |  |  |  |  |  |  |  |  |  |  |  |  |
| 0 | Vitvi10g04422\_t001 |  |  |  |  |  |  |  |  |  |  |  |  |
| 0 | Vitvi10g04423\_t001 |  |  |  |  |  |  |  |  |  |  |  |  |
| 0 | Vitvi10g01856\_t001 |  |  |  |  |  |  |  |  |  |  |  |  |
| 0 | Vitvi10g01857\_t001 |  |  |  |  |  |  |  |  |  |  |  |  |
| 0 | Vitvi10g00787\_t001 |  |  |  |  |  |  |  |  |  |  |  |  |
| 0 | Vitvi10g00788\_t001 |  |  |  |  |  |  |  |  |  |  |  |  |
| 0 | Vitvi10g01858\_t001 |  |  |  |  |  |  |  |  |  |  |  |  |
| 0 | Vitvi10g01859\_t001 |  |  |  |  |  |  |  |  |  |  |  |  |
| 0 | Vitvi10g04424\_t001 |  |  |  |  |  |  |  |  |  |  |  |  |
| 0 | Vitvi10g01860\_t001 |  |  |  |  |  |  |  |  |  |  |  |  |
| 0 | Vitvi10g00790\_t001 |  |  |  |  |  |  |  |  |  |  |  |  |
| 0 | Vitvi10g04425\_t001 |  |  |  |  |  |  |  |  |  |  |  |  |
| 0 | Vitvi10g00793\_t001 |  |  |  |  |  |  |  |  |  |  |  |  |
| 0 | Vitvi10g04426\_t001 |  |  |  |  |  |  |  |  |  |  |  |  |
| 0 | Vitvi10g00795\_t001 |  |  |  |  |  |  |  |  |  |  |  |  |
| 0 | Vitvi10g00796\_t001 |  |  |  |  |  |  |  |  |  |  |  |  |
| 0 | Vitvi10g00797\_t002 |  |  |  |  |  |  |  |  |  |  |  |  |
| 0 | Vitvi10g00798\_t001 |  |  |  |  |  |  |  |  |  |  |  |  |
| 0 | Vitvi10g04427\_t001 |  |  |  |  |  |  |  |  |  |  |  |  |
| 0 | Vitvi10g01862\_t001 |  |  |  |  |  |  |  |  |  |  |  |  |
| 0 | Vitvi10g01875\_t001 |  |  |  |  |  |  |  |  |  |  |  |  |
| 0 | Vitvi10g01864\_t001 |  |  |  |  |  |  |  |  |  |  |  |  |
| 0 | Vitvi10g01865\_t001 |  |  |  |  |  |  |  |  |  |  |  |  |
| 0 | Vitvi10g01866\_t001 |  |  |  |  |  |  |  |  |  |  |  |  |
| 0 | Vitvi10g04428\_t001 |  |  |  |  |  |  |  |  |  |  |  |  |
| 0 | Vitvi10g04429\_t001 |  |  |  |  |  |  |  |  |  |  |  |  |
| 0 | Vitvi10g04430\_t001 |  |  |  |  |  |  |  |  |  |  |  |  |
| 0 | Vitvi10g01870\_t001 |  |  |  |  |  |  |  |  |  |  |  |  |
| 0 | Vitvi10g01871\_t001 |  |  |  |  |  |  |  |  |  |  |  |  |
| 0 | Vitvi10g04431\_t001 |  |  |  |  |  |  |  |  |  |  |  |  |
| 0 | Vitvi10g04432\_t001 |  |  |  |  |  |  |  |  |  |  |  |  |
| 0 | Vitvi10g00806\_t001 |  |  |  |  |  |  |  |  |  |  |  |  |
| 0 | Vitvi10g04433\_t001 |  |  |  |  |  |  |  |  |  |  |  |  |
| 0 | Vitvi10g04434\_t001 |  |  |  |  |  |  |  |  |  |  |  |  |
| 0 | Vitvi10g04435\_t001 |  |  |  |  |  |  |  |  |  |  |  |  |
| 0 | Vitvi10g00809\_t001 |  |  |  |  |  |  |  |  |  |  |  |  |
| 0 | Vitvi10g04436\_t001 |  |  |  |  |  |  |  |  |  |  |  |  |
| 0 | Vitvi10g00811\_t001 |  |  |  |  |  |  |  |  |  |  |  |  |
| 0 | Vitvi10g00813\_t001 |  |  |  |  |  |  |  |  |  |  |  |  |
| 0 | Vitvi10g00814\_t001 |  |  |  |  |  |  |  |  |  |  |  |  |
| 0 | Vitvi10g04437\_t001 |  |  |  |  |  |  |  |  |  |  |  |  |
| 0 | Vitvi10g00816\_t001 |  |  |  |  |  |  |  |  |  |  |  |  |
| 0 | Vitvi10g04438\_t001 |  |  |  |  |  |  |  |  |  |  |  |  |
| 0 | Vitvi10g04439\_t001 |  |  |  |  |  |  |  |  |  |  |  |  |
| 0 | Vitvi10g00821\_t001 |  |  |  |  |  |  |  |  |  |  |  |  |
| 0 | Vitvi10g04440\_t001 |  |  |  |  |  |  |  |  |  |  |  |  |
| 0 | Vitvi10g00825\_t002 |  |  |  |  |  |  |  |  |  |  |  |  |
| 0 | Vitvi10g00826\_t001 |  |  |  |  |  |  |  |  |  |  |  |  |
| 0 | Vitvi10g00827\_t001 |  |  |  |  |  |  |  |  |  |  |  |  |
| 0 | Vitvi10g00828\_t001 |  |  |  |  |  |  |  |  |  |  |  |  |
| 0 | Vitvi10g00829\_t001 |  |  |  |  |  |  |  |  |  |  |  |  |
| 0 | Vitvi10g01880\_t001 |  |  |  |  |  |  |  |  |  |  |  |  |
| 0 | Vitvi10g04441\_t001 |  |  |  |  |  |  |  |  |  |  |  |  |
| 0 | Vitvi10g00831\_t001 |  |  |  |  |  |  |  |  |  |  |  |  |
| 0 | Vitvi10g01881\_t001 |  |  |  |  |  |  |  |  |  |  |  |  |
| 0 | Vitvi10g00832\_t001 |  |  |  |  |  |  |  |  |  |  |  |  |
| 0 | Vitvi10g04442\_t001 |  |  |  |  |  |  |  |  |  |  |  |  |
| 0 | Vitvi10g00834\_t001 |  |  |  |  |  |  |  |  |  |  |  |  |
| 0 | Vitvi10g00835\_t001 |  |  |  |  |  |  |  |  |  |  |  |  |
| 0 | Vitvi10g00837\_t001 |  |  |  |  |  |  |  |  |  |  |  |  |
| 0 | Vitvi10g00838\_t001 |  |  |  |  |  |  |  |  |  |  |  |  |
| 0 | Vitvi10g04443\_t001 |  |  |  |  |  |  |  |  |  |  |  |  |
| 0 | Vitvi10g04444\_t001 |  |  |  |  |  |  |  |  |  |  |  |  |
| 0 | Vitvi10g01883\_t001 |  |  |  |  |  |  |  |  |  |  |  |  |
| 0 | Vitvi10g00839\_t001 |  |  |  |  |  |  |  |  |  |  |  |  |
| 0 | Vitvi10g01884\_t001 |  |  |  |  |  |  |  |  |  |  |  |  |
| 0 | Vitvi10g04445\_t001 |  |  |  |  |  |  |  |  |  |  |  |  |
| 0 | Vitvi10g00841\_t001 |  |  |  |  |  |  |  |  |  |  |  |  |
| 0 | Vitvi10g01885\_t001 |  |  |  |  |  |  |  |  |  |  |  |  |
| 0 | Vitvi10g00842\_t001 |  |  |  |  |  |  |  |  |  |  |  |  |
| 0 | Vitvi10g00843\_t001 |  |  |  |  |  |  |  |  |  |  |  |  |
| 0 | Vitvi10g00844\_t001 |  |  |  |  |  |  |  |  |  |  |  |  |
| 0 | Vitvi10g01886\_t001 |  |  |  |  |  |  |  |  |  |  |  |  |
| 0 | Vitvi10g04446\_t001 |  |  |  |  |  |  |  |  |  |  |  |  |
| 0 | Vitvi10g00845\_t001 |  |  |  |  |  |  |  |  |  |  |  |  |
| 0 | Vitvi10g01887\_t001 |  |  |  |  |  |  |  |  |  |  |  |  |
| 0 | Vitvi10g00848\_t001 |  |  |  |  |  |  |  |  |  |  |  |  |
| 0 | Vitvi10g00849\_t001 |  |  |  |  |  |  |  |  |  |  |  |  |
| 0 | Vitvi10g01888\_t001 |  |  |  |  |  |  |  |  |  |  |  |  |
| 0 | Vitvi10g00850\_t001 |  |  |  |  |  |  |  |  |  |  |  |  |
| 0 | Vitvi10g00851\_t002 |  |  |  |  |  |  |  |  |  |  |  |  |
| 0 | Vitvi10g00852\_t001 |  |  |  |  |  |  |  |  |  |  |  |  |
| 0 | Vitvi10g00853\_t001 |  |  |  |  |  |  |  |  |  |  |  |  |
| 0 | Vitvi10g00854\_t001 |  |  |  |  |  |  |  |  |  |  |  |  |
| 0 | Vitvi10g00855\_t001 |  |  |  |  |  |  |  |  |  |  |  |  |
| 0 | Vitvi10g04447\_t001 |  |  |  |  |  |  |  |  |  |  |  |  |
| 0 | Vitvi10g00858\_t001 |  |  |  |  |  |  |  |  |  |  |  |  |
| 0 | Vitvi10g00860\_t001 |  |  |  |  |  |  |  |  |  |  |  |  |
| 0 | Vitvi10g04448\_t001 |  |  |  |  |  |  |  |  |  |  |  |  |
| 0 | Vitvi10g00862\_t001 |  |  |  |  |  |  |  |  |  |  |  |  |
| 0 | Vitvi10g04449\_t001 |  |  |  |  |  |  |  |  |  |  |  |  |
| 0 | Vitvi10g04450\_t001 |  |  |  |  |  |  |  |  |  |  |  |  |
| 0 | Vitvi10g00866\_t001 |  |  |  |  |  |  |  |  |  |  |  |  |
| 0 | Vitvi10g00867\_t001 |  |  |  |  |  |  |  |  |  |  |  |  |
| 0 | Vitvi10g00868\_t001 |  |  |  |  |  |  |  |  |  |  |  |  |
| 0 | Vitvi10g00869\_t001 |  |  |  |  |  |  |  |  |  |  |  |  |
| 0 | Vitvi10g00870\_t001 |  |  |  |  |  |  |  |  |  |  |  |  |
| 0 | Vitvi10g04451\_t001 |  |  |  |  |  |  |  |  |  |  |  |  |
| 0 | Vitvi10g04452\_t001 |  |  |  |  |  |  |  |  |  |  |  |  |
| 0 | Vitvi10g04453\_t001 |  |  |  |  |  |  |  |  |  |  |  |  |
| 0 | Vitvi10g04454\_t001 |  |  |  |  |  |  |  |  |  |  |  |  |
| 0 | Vitvi10g04455\_t001 |  |  |  |  |  |  |  |  |  |  |  |  |
| 0 | Vitvi10g00872\_t001 |  |  |  |  |  |  |  |  |  |  |  |  |
| 0 | Vitvi10g00873\_t001 |  |  |  |  |  |  |  |  |  |  |  |  |
| 0 | Vitvi10g00876\_t001 |  |  |  |  |  |  |  |  |  |  |  |  |
| 0 | Vitvi10g04456\_t001 |  |  |  |  |  |  |  |  |  |  |  |  |
| 0 | Vitvi10g01892\_t001 |  |  |  |  |  |  |  |  |  |  |  |  |
| 0 | Vitvi10g04457\_t001 |  |  |  |  |  |  |  |  |  |  |  |  |
| 0 | Vitvi10g01893\_t001 |  |  |  |  |  |  |  |  |  |  |  |  |
| 0 | Vitvi10g01894\_t001 |  |  |  |  |  |  |  |  |  |  |  |  |
| 0 | Vitvi10g00877\_t001 |  |  |  |  |  |  |  |  |  |  |  |  |
| 0 | Vitvi10g00878\_t001 |  |  |  |  |  |  |  |  |  |  |  |  |
| 0 | Vitvi10g04458\_t001 |  |  |  |  |  |  |  |  |  |  |  |  |
| 0 | Vitvi10g04459\_t001 |  |  |  |  |  |  |  |  |  |  |  |  |
| 0 | Vitvi10g00879\_t001 |  |  |  |  |  |  |  |  |  |  |  |  |
| 0 | Vitvi10g04460\_t001 |  |  |  |  |  |  |  |  |  |  |  |  |
| 0 | Vitvi10g04461\_t001 |  |  |  |  |  |  |  |  |  |  |  |  |
| 0 | Vitvi10g00880\_t001 |  |  |  |  |  |  |  |  |  |  |  |  |
| 0 | Vitvi10g04462\_t001 |  |  |  |  |  |  |  |  |  |  |  |  |
| 0 | Vitvi10g00881\_t001 |  |  |  |  |  |  |  |  |  |  |  |  |
| 0 | Vitvi10g00882\_t001 |  |  |  |  |  |  |  |  |  |  |  |  |
| 0 | Vitvi10g00884\_t001 |  |  |  |  |  |  |  |  |  |  |  |  |
| 0 | Vitvi10g00885\_t001 |  |  |  |  |  |  |  |  |  |  |  |  |
| 0 | Vitvi10g00886\_t001 |  |  |  |  |  |  |  |  |  |  |  |  |
| 0 | Vitvi10g04463\_t001 |  |  |  |  |  |  |  |  |  |  |  |  |
| 0 | Vitvi10g04464\_t001 |  |  |  |  |  |  |  |  |  |  |  |  |
| 0 | Vitvi10g00888\_t001 |  |  |  |  |  |  |  |  |  |  |  |  |
| 0 | Vitvi10g00889\_t001 |  |  |  |  |  |  |  |  |  |  |  |  |
| 0 | Vitvi10g04465\_t001 |  |  |  |  |  |  |  |  |  |  |  |  |
| 0 | Vitvi10g00892\_t001 |  |  |  |  |  |  |  |  |  |  |  |  |
| 0 | Vitvi10g04466\_t001 |  |  |  |  |  |  |  |  |  |  |  |  |
| 0 | Vitvi10g00895\_t002 |  |  |  |  |  |  |  |  |  |  |  |  |
| 0 | Vitvi10g04467\_t001 |  |  |  |  |  |  |  |  |  |  |  |  |
| 0 | Vitvi10g04468\_t001 |  |  |  |  |  |  |  |  |  |  |  |  |
| 0 | Vitvi10g00899\_t001 |  |  |  |  |  |  |  |  |  |  |  |  |
| 0 | Vitvi10g00902\_t003 |  |  |  |  |  |  |  |  |  |  |  |  |
| 0 | Vitvi10g00903\_t001 |  |  |  |  |  |  |  |  |  |  |  |  |
| 0 | Vitvi10g00904\_t001 |  |  |  |  |  |  |  |  |  |  |  |  |
| 0 | Vitvi10g00905\_t001 |  |  |  |  |  |  |  |  |  |  |  |  |
| 0 | Vitvi10g04469\_t001 |  |  |  |  |  |  |  |  |  |  |  |  |
| 0 | Vitvi10g04470\_t001 |  |  |  |  |  |  |  |  |  |  |  |  |
| 0 | Vitvi10g00906\_t001 |  |  |  |  |  |  |  |  |  |  |  |  |
| 0 | Vitvi10g04471\_t001 |  |  |  |  |  |  |  |  |  |  |  |  |
| 0 | Vitvi10g00907\_t001 |  |  |  |  |  |  |  |  |  |  |  |  |
| 0 | Vitvi10g00908\_t002 |  |  |  |  |  |  |  |  |  |  |  |  |
| 0 | Vitvi10g00910\_t001 |  |  |  |  |  |  |  |  |  |  |  |  |
| 0 | Vitvi10g04472\_t001 |  |  |  |  |  |  |  |  |  |  |  |  |
| 0 | Vitvi10g00911\_t001 |  |  |  |  |  |  |  |  |  |  |  |  |
| 0 | Vitvi10g04473\_t001 |  |  |  |  |  |  |  |  |  |  |  |  |
| 0 | Vitvi10g00913\_t001 |  |  |  |  |  |  |  |  |  |  |  |  |
| 0 | Vitvi10g00914\_t001 |  |  |  |  |  |  |  |  |  |  |  |  |
| 0 | Vitvi10g01901\_t001 |  |  |  |  |  |  |  |  |  |  |  |  |
| 0 | Vitvi10g00915\_t001 |  |  |  |  |  |  |  |  |  |  |  |  |
| 0 | Vitvi10g00917\_t001 |  |  |  |  |  |  |  |  |  |  |  |  |
| 0 | Vitvi10g04474\_t001 |  |  |  |  |  |  |  |  |  |  |  |  |
| 0 | Vitvi10g04475\_t001 |  |  |  |  |  |  |  |  |  |  |  |  |
| 0 | Vitvi10g04476\_t001 |  |  |  |  |  |  |  |  |  |  |  |  |
| 0 | Vitvi10g04477\_t001 |  |  |  |  |  |  |  |  |  |  |  |  |
| 0 | Vitvi10g04478\_t001 |  |  |  |  |  |  |  |  |  |  |  |  |
| 0 | Vitvi10g04479\_t001 |  |  |  |  |  |  |  |  |  |  |  |  |
| 0 | Vitvi10g01906\_t001 |  |  |  |  |  |  |  |  |  |  |  |  |
| 0 | Vitvi10g01907\_t001 |  |  |  |  |  |  |  |  |  |  |  |  |
| 0 | Vitvi10g04480\_t001 |  |  |  |  |  |  |  |  |  |  |  |  |
| 0 | Vitvi10g04481\_t001 |  |  |  |  |  |  |  |  |  |  |  |  |
| 0 | Vitvi10g00928\_t001 |  |  |  |  |  |  |  |  |  |  |  |  |
| 0 | Vitvi10g04482\_t001 |  |  |  |  |  |  |  |  |  |  |  |  |
| 0 | Vitvi10g04483\_t001 |  |  |  |  |  |  |  |  |  |  |  |  |
| 0 | Vitvi10g04484\_t001 |  |  |  |  |  |  |  |  |  |  |  |  |
| 0 | Vitvi10g04485\_t001 |  |  |  |  |  |  |  |  |  |  |  |  |
| 0 | Vitvi10g01909\_t001 |  |  |  |  |  |  |  |  |  |  |  |  |
| 0 | Vitvi10g04486\_t001 |  |  |  |  |  |  |  |  |  |  |  |  |
| 0 | Vitvi10g04487\_t001 |  |  |  |  |  |  |  |  |  |  |  |  |
| 0 | Vitvi10g04488\_t001 |  |  |  |  |  |  |  |  |  |  |  |  |
| 0 | Vitvi10g00931\_t001 |  |  |  |  |  |  |  |  |  |  |  |  |
| 0 | Vitvi10g04489\_t001 |  |  |  |  |  |  |  |  |  |  |  |  |
| 0 | Vitvi10g01910\_t001 |  |  |  |  |  |  |  |  |  |  |  |  |
| 0 | Vitvi10g04490\_t001 |  |  |  |  |  |  |  |  |  |  |  |  |
| 0 | Vitvi10g04491\_t001 |  |  |  |  |  |  |  |  |  |  |  |  |
| 0 | Vitvi10g00934\_t001 |  |  |  |  |  |  |  |  |  |  |  |  |
| 0 | Vitvi10g00936\_t001 |  |  |  |  |  |  |  |  |  |  |  |  |
| 0 | Vitvi10g00937\_t001 |  |  |  |  |  |  |  |  |  |  |  |  |
| 0 | Vitvi10g00938\_t001 |  |  |  |  |  |  |  |  |  |  |  |  |
| 0 | Vitvi10g01912\_t001 |  |  |  |  |  |  |  |  |  |  |  |  |
| 0 | Vitvi10g01913\_t001 |  |  |  |  |  |  |  |  |  |  |  |  |
| 0 | Vitvi10g00940\_t001 |  |  |  |  |  |  |  |  |  |  |  |  |
| 0 | Vitvi10g01915\_t001 |  |  |  |  |  |  |  |  |  |  |  |  |
| 0 | Vitvi10g00941\_t002 |  |  |  |  |  |  |  |  |  |  |  |  |
| 0 | Vitvi10g00944\_t001 |  |  |  |  |  |  |  |  |  |  |  |  |
| 0 | Vitvi10g00946\_t001 |  |  |  |  |  |  |  |  |  |  |  |  |
| 0 | Vitvi10g00947\_t001 |  |  |  |  |  |  |  |  |  |  |  |  |
| 0 | Vitvi10g04492\_t001 |  |  |  |  |  |  |  |  |  |  |  |  |
| 0 | Vitvi10g00948\_t001 |  |  |  |  |  |  |  |  |  |  |  |  |
| 0 | Vitvi10g00949\_t001 |  |  |  |  |  |  |  |  |  |  |  |  |
| 0 | Vitvi10g00950\_t001 |  |  |  |  |  |  |  |  |  |  |  |  |
| 0 | Vitvi10g00952\_t001 |  |  |  |  |  |  |  |  |  |  |  |  |
| 0 | Vitvi10g04493\_t001 |  |  |  |  |  |  |  |  |  |  |  |  |
| 0 | Vitvi10g00954\_t001 |  |  |  |  |  |  |  |  |  |  |  |  |
| 0 | Vitvi10g00955\_t001 |  |  |  |  |  |  |  |  |  |  |  |  |
| 0 | Vitvi10g01916\_t001 |  |  |  |  |  |  |  |  |  |  |  |  |
| 0 | Vitvi10g00957\_t001 |  |  |  |  |  |  |  |  |  |  |  |  |
| 0 | Vitvi10g00959\_t001 |  |  |  |  |  |  |  |  |  |  |  |  |
| 0 | Vitvi10g00960\_t001 |  |  |  |  |  |  |  |  |  |  |  |  |
| 0 | Vitvi10g00961\_t001 |  |  |  |  |  |  |  |  |  |  |  |  |
| 0 | Vitvi10g00963\_t001 |  |  |  |  |  |  |  |  |  |  |  |  |
| 0 | Vitvi10g04494\_t001 |  |  |  |  |  |  |  |  |  |  |  |  |
| 0 | Vitvi10g00964\_t001 |  |  |  |  |  |  |  |  |  |  |  |  |
| 0 | Vitvi10g00966\_t001 |  |  |  |  |  |  |  |  |  |  |  |  |
| 0 | Vitvi10g04495\_t001 |  |  |  |  |  |  |  |  |  |  |  |  |
| 0 | Vitvi10g04496\_t001 |  |  |  |  |  |  |  |  |  |  |  |  |
| 0 | Vitvi10g00968\_t001 |  |  |  |  |  |  |  |  |  |  |  |  |
| 0 | Vitvi10g00969\_t001 |  |  |  |  |  |  |  |  |  |  |  |  |
| 0 | Vitvi10g04497\_t001 |  |  |  |  |  |  |  |  |  |  |  |  |
| 0 | Vitvi10g04498\_t001 |  |  |  |  |  |  |  |  |  |  |  |  |
| 0 | Vitvi10g00973\_t001 |  |  |  |  |  |  |  |  |  |  |  |  |
| 0 | Vitvi10g00977\_t001 |  |  |  |  |  |  |  |  |  |  |  |  |
| 0 | Vitvi10g01921\_t001 |  |  |  |  |  |  |  |  |  |  |  |  |
| 0 | Vitvi10g04499\_t001 |  |  |  |  |  |  |  |  |  |  |  |  |
| 0 | Vitvi10g04500\_t001 |  |  |  |  |  |  |  |  |  |  |  |  |
| 0 | Vitvi10g04501\_t001 |  |  |  |  |  |  |  |  |  |  |  |  |
| 0 | Vitvi10g00982\_t001 |  |  |  |  |  |  |  |  |  |  |  |  |
| 0 | Vitvi10g00984\_t001 |  |  |  |  |  |  |  |  |  |  |  |  |
| 0 | Vitvi10g04502\_t001 |  |  |  |  |  |  |  |  |  |  |  |  |
| 0 | Vitvi10g04503\_t001 |  |  |  |  |  |  |  |  |  |  |  |  |
| 0 | Vitvi10g04504\_t001 |  |  |  |  |  |  |  |  |  |  |  |  |
| 0 | Vitvi10g00988\_t001 |  |  |  |  |  |  |  |  |  |  |  |  |
| 0 | Vitvi10g00990\_t001 |  |  |  |  |  |  |  |  |  |  |  |  |
| 0 | Vitvi10g00992\_t001 |  |  |  |  |  |  |  |  |  |  |  |  |
| 0 | Vitvi10g00996\_t001 |  |  |  |  |  |  |  |  |  |  |  |  |
| 0 | Vitvi10g01926\_t001 |  |  |  |  |  |  |  |  |  |  |  |  |
| 0 | Vitvi10g01929\_t001 |  |  |  |  |  |  |  |  |  |  |  |  |
| 0 | Vitvi10g04505\_t001 |  |  |  |  |  |  |  |  |  |  |  |  |
| 0 | Vitvi10g04506\_t001 |  |  |  |  |  |  |  |  |  |  |  |  |
| 0 | Vitvi10g01005\_t001 |  |  |  |  |  |  |  |  |  |  |  |  |
| 0 | Vitvi10g01006\_t001 |  |  |  |  |  |  |  |  |  |  |  |  |
| 0 | Vitvi10g01931\_t001 |  |  |  |  |  |  |  |  |  |  |  |  |
| 0 | Vitvi10g01007\_t001 |  |  |  |  |  |  |  |  |  |  |  |  |
| 0 | Vitvi10g04507\_t001 |  |  |  |  |  |  |  |  |  |  |  |  |
| 0 | Vitvi10g04508\_t001 |  |  |  |  |  |  |  |  |  |  |  |  |
| 0 | Vitvi10g01012\_t001 |  |  |  |  |  |  |  |  |  |  |  |  |
| 0 | Vitvi10g01015\_t001 |  |  |  |  |  |  |  |  |  |  |  |  |
| 0 | Vitvi10g01016\_t001 |  |  |  |  |  |  |  |  |  |  |  |  |
| 0 | Vitvi10g01017\_t001 |  |  |  |  |  |  |  |  |  |  |  |  |
| 0 | Vitvi10g01019\_t001 |  |  |  |  |  |  |  |  |  |  |  |  |
| 0 | Vitvi10g01020\_t001 |  |  |  |  |  |  |  |  |  |  |  |  |
| 0 | Vitvi10g04509\_t001 |  |  |  |  |  |  |  |  |  |  |  |  |
| 0 | Vitvi10g01028\_t001 |  |  |  |  |  |  |  |  |  |  |  |  |
| 0 | Vitvi10g04510\_t001 |  |  |  |  |  |  |  |  |  |  |  |  |
| 0 | Vitvi10g01029\_t001 |  |  |  |  |  |  |  |  |  |  |  |  |
| 0 | Vitvi10g01032\_t001 |  |  |  |  |  |  |  |  |  |  |  |  |
| 0 | Vitvi10g01035\_t001 |  |  |  |  |  |  |  |  |  |  |  |  |
| 0 | Vitvi10g01037\_t001 |  |  |  |  |  |  |  |  |  |  |  |  |
| 0 | Vitvi10g04511\_t001 |  |  |  |  |  |  |  |  |  |  |  |  |
| 0 | Vitvi10g01040\_t001 |  |  |  |  |  |  |  |  |  |  |  |  |
| 0 | Vitvi10g01041\_t001 |  |  |  |  |  |  |  |  |  |  |  |  |
| 0 | Vitvi10g01042\_t001 |  |  |  |  |  |  |  |  |  |  |  |  |
| 0 | Vitvi10g04512\_t001 |  |  |  |  |  |  |  |  |  |  |  |  |
| 0 | Vitvi10g04513\_t001 |  |  |  |  |  |  |  |  |  |  |  |  |
| 0 | Vitvi10g01044\_t001 |  |  |  |  |  |  |  |  |  |  |  |  |
| 0 | Vitvi10g01045\_t001 |  |  |  |  |  |  |  |  |  |  |  |  |
| 0 | Vitvi10g01046\_t001 |  |  |  |  |  |  |  |  |  |  |  |  |
| 0 | Vitvi10g04514\_t001 |  |  |  |  |  |  |  |  |  |  |  |  |
| 0 | Vitvi10g01053\_t001 |  |  |  |  |  |  |  |  |  |  |  |  |
| 0 | Vitvi10g01055\_t001 |  |  |  |  |  |  |  |  |  |  |  |  |
| 0 | Vitvi10g01056\_t001 |  |  |  |  |  |  |  |  |  |  |  |  |
| 0 | Vitvi10g01935\_t001 |  |  |  |  |  |  |  |  |  |  |  |  |
| 0 | Vitvi10g01060\_t001 |  |  |  |  |  |  |  |  |  |  |  |  |
| 0 | Vitvi10g01061\_t001 |  |  |  |  |  |  |  |  |  |  |  |  |
| 0 | Vitvi10g01062\_t001 |  |  |  |  |  |  |  |  |  |  |  |  |
| 0 | Vitvi10g01065\_t001 |  |  |  |  |  |  |  |  |  |  |  |  |
| 0 | Vitvi10g01066\_t001 |  |  |  |  |  |  |  |  |  |  |  |  |
| 0 | Vitvi10g01067\_t001 |  |  |  |  |  |  |  |  |  |  |  |  |
| 0 | Vitvi10g01068\_t001 |  |  |  |  |  |  |  |  |  |  |  |  |
| 0 | Vitvi10g01070\_t001 |  |  |  |  |  |  |  |  |  |  |  |  |
| 0 | Vitvi10g01072\_t001 |  |  |  |  |  |  |  |  |  |  |  |  |
| 0 | Vitvi10g01936\_t001 |  |  |  |  |  |  |  |  |  |  |  |  |
| 0 | Vitvi10g01075\_t001 |  |  |  |  |  |  |  |  |  |  |  |  |
| 0 | Vitvi10g04515\_t001 |  |  |  |  |  |  |  |  |  |  |  |  |
| 0 | Vitvi10g01077\_t001 |  |  |  |  |  |  |  |  |  |  |  |  |
| 0 | Vitvi10g01078\_t001 |  |  |  |  |  |  |  |  |  |  |  |  |
| 0 | Vitvi10g04516\_t001 |  |  |  |  |  |  |  |  |  |  |  |  |
| 0 | Vitvi10g04517\_t001 |  |  |  |  |  |  |  |  |  |  |  |  |
| 0 | Vitvi10g04518\_t001 |  |  |  |  |  |  |  |  |  |  |  |  |
| 0 | Vitvi10g01940\_t001 |  |  |  |  |  |  |  |  |  |  |  |  |
| 0 | Vitvi10g01084\_t001 |  |  |  |  |  |  |  |  |  |  |  |  |
| 0 | Vitvi10g01942\_t001 |  |  |  |  |  |  |  |  |  |  |  |  |
| 0 | Vitvi10g01943\_t001 |  |  |  |  |  |  |  |  |  |  |  |  |
| 0 | Vitvi10g01086\_t001 |  |  |  |  |  |  |  |  |  |  |  |  |
| 0 | Vitvi10g01088\_t001 |  |  |  |  |  |  |  |  |  |  |  |  |
| 0 | Vitvi10g01089\_t001 |  |  |  |  |  |  |  |  |  |  |  |  |
| 0 | Vitvi10g01090\_t001 |  |  |  |  |  |  |  |  |  |  |  |  |
| 0 | Vitvi10g04519\_t002 |  |  |  |  |  |  |  |  |  |  |  |  |
| 0 | Vitvi10g01945\_t001 |  |  |  |  |  |  |  |  |  |  |  |  |
| 0 | Vitvi10g01092\_t001 |  |  |  |  |  |  |  |  |  |  |  |  |
| 0 | Vitvi10g01093\_t001 |  |  |  |  |  |  |  |  |  |  |  |  |
| 0 | Vitvi10g04520\_t001 |  |  |  |  |  |  |  |  |  |  |  |  |
| 0 | Vitvi10g01094\_t001 |  |  |  |  |  |  |  |  |  |  |  |  |
| 0 | Vitvi10g01095\_t001 |  |  |  |  |  |  |  |  |  |  |  |  |
| 0 | Vitvi10g04521\_t001 |  |  |  |  |  |  |  |  |  |  |  |  |
| 0 | Vitvi10g04522\_t001 |  |  |  |  |  |  |  |  |  |  |  |  |
| 0 | Vitvi10g01946\_t001 |  |  |  |  |  |  |  |  |  |  |  |  |
| 0 | Vitvi10g04523\_t001 |  |  |  |  |  |  |  |  |  |  |  |  |
| 0 | Vitvi10g01097\_t001 |  |  |  |  |  |  |  |  |  |  |  |  |
| 0 | Vitvi10g01947\_t001 |  |  |  |  |  |  |  |  |  |  |  |  |
| 0 | Vitvi10g01098\_t001 |  |  |  |  |  |  |  |  |  |  |  |  |
| 0 | Vitvi10g01099\_t001 |  |  |  |  |  |  |  |  |  |  |  |  |
| 0 | Vitvi10g04524\_t001 |  |  |  |  |  |  |  |  |  |  |  |  |
| 0 | Vitvi10g04525\_t001 |  |  |  |  |  |  |  |  |  |  |  |  |
| 0 | Vitvi10g04526\_t001 |  |  |  |  |  |  |  |  |  |  |  |  |
| 0 | Vitvi10g01103\_t001 |  |  |  |  |  |  |  |  |  |  |  |  |
| 0 | Vitvi10g01107\_t001 |  |  |  |  |  |  |  |  |  |  |  |  |
| 0 | Vitvi10g01948\_t001 |  |  |  |  |  |  |  |  |  |  |  |  |
| 0 | Vitvi10g01949\_t001 |  |  |  |  |  |  |  |  |  |  |  |  |
| 0 | Vitvi10g01110\_t001 |  |  |  |  |  |  |  |  |  |  |  |  |
| 0 | Vitvi10g01111\_t001 |  |  |  |  |  |  |  |  |  |  |  |  |
| 0 | Vitvi10g01112\_t001 |  |  |  |  |  |  |  |  |  |  |  |  |
| 0 | Vitvi10g01113\_t001 |  |  |  |  |  |  |  |  |  |  |  |  |
| 0 | Vitvi10g04527\_t001 |  |  |  |  |  |  |  |  |  |  |  |  |
| 0 | Vitvi10g04528\_t001 |  |  |  |  |  |  |  |  |  |  |  |  |
| 0 | Vitvi10g01116\_t001 |  |  |  |  |  |  |  |  |  |  |  |  |
| 0 | Vitvi10g04529\_t001 |  |  |  |  |  |  |  |  |  |  |  |  |
| 0 | Vitvi10g01120\_t001 |  |  |  |  |  |  |  |  |  |  |  |  |
| 0 | Vitvi10g01121\_t001 |  |  |  |  |  |  |  |  |  |  |  |  |
| 0 | Vitvi10g04530\_t001 |  |  |  |  |  |  |  |  |  |  |  |  |
| 0 | Vitvi10g01123\_t002 |  |  |  |  |  |  |  |  |  |  |  |  |
| 0 | Vitvi10g04531\_t001 |  |  |  |  |  |  |  |  |  |  |  |  |
| 0 | Vitvi10g01952\_t001 |  |  |  |  |  |  |  |  |  |  |  |  |
| 0 | Vitvi10g04532\_t001 |  |  |  |  |  |  |  |  |  |  |  |  |
| 0 | Vitvi10g04533\_t001 |  |  |  |  |  |  |  |  |  |  |  |  |
| 0 | Vitvi10g04534\_t001 |  |  |  |  |  |  |  |  |  |  |  |  |
| 0 | Vitvi10g04535\_t001 |  |  |  |  |  |  |  |  |  |  |  |  |
| 0 | Vitvi10g01955\_t001 |  |  |  |  |  |  |  |  |  |  |  |  |
| 0 | Vitvi10g01134\_t001 |  |  |  |  |  |  |  |  |  |  |  |  |
| 0 | Vitvi10g01135\_t001 |  |  |  |  |  |  |  |  |  |  |  |  |
| 0 | Vitvi10g01136\_t001 |  |  |  |  |  |  |  |  |  |  |  |  |
| 0 | Vitvi10g04536\_t001 |  |  |  |  |  |  |  |  |  |  |  |  |
| 0 | Vitvi10g04537\_t001 |  |  |  |  |  |  |  |  |  |  |  |  |
| 0 | Vitvi10g01138\_t001 |  |  |  |  |  |  |  |  |  |  |  |  |
| 0 | Vitvi10g01140\_t001 |  |  |  |  |  |  |  |  |  |  |  |  |
| 0 | Vitvi10g04538\_t001 |  |  |  |  |  |  |  |  |  |  |  |  |
| 0 | Vitvi10g01141\_t001 |  |  |  |  |  |  |  |  |  |  |  |  |
| 0 | Vitvi10g04539\_t001 |  |  |  |  |  |  |  |  |  |  |  |  |
| 0 | Vitvi10g04540\_t001 |  |  |  |  |  |  |  |  |  |  |  |  |
| 0 | Vitvi10g01151\_t001 |  |  |  |  |  |  |  |  |  |  |  |  |
| 0 | Vitvi10g04541\_t001 |  |  |  |  |  |  |  |  |  |  |  |  |
| 0 | Vitvi10g04542\_t001 |  |  |  |  |  |  |  |  |  |  |  |  |
| 0 | Vitvi10g01153\_t001 |  |  |  |  |  |  |  |  |  |  |  |  |
| 0 | Vitvi10g01154\_t001 |  |  |  |  |  |  |  |  |  |  |  |  |
| 0 | Vitvi10g04543\_t001 |  |  |  |  |  |  |  |  |  |  |  |  |
| 0 | Vitvi10g01957\_t001 |  |  |  |  |  |  |  |  |  |  |  |  |
| 0 | Vitvi10g01155\_t001 |  |  |  |  |  |  |  |  |  |  |  |  |
| 0 | Vitvi10g01156\_t001 |  |  |  |  |  |  |  |  |  |  |  |  |
| 0 | Vitvi10g04544\_t001 |  |  |  |  |  |  |  |  |  |  |  |  |
| 0 | Vitvi10g01161\_t001 |  |  |  |  |  |  |  |  |  |  |  |  |
| 0 | Vitvi10g04545\_t001 |  |  |  |  |  |  |  |  |  |  |  |  |
| 0 | Vitvi10g01162\_t001 |  |  |  |  |  |  |  |  |  |  |  |  |
| 0 | Vitvi10g01163\_t001 |  |  |  |  |  |  |  |  |  |  |  |  |
| 0 | Vitvi10g01164\_t001 |  |  |  |  |  |  |  |  |  |  |  |  |
| 0 | Vitvi10g01166\_t001 |  |  |  |  |  |  |  |  |  |  |  |  |
| 0 | Vitvi10g01958\_t001 |  |  |  |  |  |  |  |  |  |  |  |  |
| 0 | Vitvi10g01168\_t001 |  |  |  |  |  |  |  |  |  |  |  |  |
| 0 | Vitvi10g04546\_t001 |  |  |  |  |  |  |  |  |  |  |  |  |
| 0 | Vitvi10g04547\_t001 |  |  |  |  |  |  |  |  |  |  |  |  |
| 0 | Vitvi10g01173\_t002 |  |  |  |  |  |  |  |  |  |  |  |  |
| 0 | Vitvi10g04548\_t001 |  |  |  |  |  |  |  |  |  |  |  |  |
| 0 | Vitvi10g04549\_t001 |  |  |  |  |  |  |  |  |  |  |  |  |
| 0 | Vitvi10g01178\_t001 |  |  |  |  |  |  |  |  |  |  |  |  |
| 0 | Vitvi10g01181\_t001 |  |  |  |  |  |  |  |  |  |  |  |  |
| 0 | Vitvi10g01182\_t001 |  |  |  |  |  |  |  |  |  |  |  |  |
| 0 | Vitvi10g01183\_t001 |  |  |  |  |  |  |  |  |  |  |  |  |
| 0 | Vitvi10g01190\_t001 |  |  |  |  |  |  |  |  |  |  |  |  |
| 0 | Vitvi10g04551\_t001 |  |  |  |  |  |  |  |  |  |  |  |  |
| 0 | Vitvi10g04552\_t001 |  |  |  |  |  |  |  |  |  |  |  |  |
| 0 | Vitvi10g04553\_t001 |  |  |  |  |  |  |  |  |  |  |  |  |
| 0 | Vitvi10g04554\_t001 |  |  |  |  |  |  |  |  |  |  |  |  |
| 0 | Vitvi10g04555\_t001 |  |  |  |  |  |  |  |  |  |  |  |  |
| 0 | Vitvi10g01194\_t001 |  |  |  |  |  |  |  |  |  |  |  |  |
| 0 | Vitvi10g01195\_t001 |  |  |  |  |  |  |  |  |  |  |  |  |
| 0 | Vitvi10g04556\_t001 |  |  |  |  |  |  |  |  |  |  |  |  |
| 0 | Vitvi10g04557\_t001 |  |  |  |  |  |  |  |  |  |  |  |  |
| 0 | Vitvi10g04558\_t001 |  |  |  |  |  |  |  |  |  |  |  |  |
| 0 | Vitvi10g04559\_t001 |  |  |  |  |  |  |  |  |  |  |  |  |
| 0 | Vitvi10g01962\_t001 |  |  |  |  |  |  |  |  |  |  |  |  |
| 0 | Vitvi10g01963\_t001 |  |  |  |  |  |  |  |  |  |  |  |  |
| 0 | Vitvi10g04560\_t001 |  |  |  |  |  |  |  |  |  |  |  |  |
| 0 | Vitvi10g04561\_t001 |  |  |  |  |  |  |  |  |  |  |  |  |
| 0 | Vitvi10g04562\_t001 |  |  |  |  |  |  |  |  |  |  |  |  |
| 0 | Vitvi10g04563\_t001 |  |  |  |  |  |  |  |  |  |  |  |  |
| 0 | Vitvi10g04564\_t001 |  |  |  |  |  |  |  |  |  |  |  |  |
| 0 | Vitvi10g04565\_t001 |  |  |  |  |  |  |  |  |  |  |  |  |
| 0 | Vitvi10g04566\_t001 |  |  |  |  |  |  |  |  |  |  |  |  |
| 0 | Vitvi10g04567\_t001 |  |  |  |  |  |  |  |  |  |  |  |  |
| 0 | Vitvi10g04568\_t001 |  |  |  |  |  |  |  |  |  |  |  |  |
| 0 | Vitvi10g04569\_t001 |  |  |  |  |  |  |  |  |  |  |  |  |
| 0 | Vitvi10g04570\_t001 |  |  |  |  |  |  |  |  |  |  |  |  |
| 0 | Vitvi10g04571\_t001 |  |  |  |  |  |  |  |  |  |  |  |  |
| 0 | Vitvi10g04572\_t001 |  |  |  |  |  |  |  |  |  |  |  |  |
| 0 | Vitvi10g01207\_t001 |  |  |  |  |  |  |  |  |  |  |  |  |
| 0 | Vitvi10g04573\_t001 |  |  |  |  |  |  |  |  |  |  |  |  |
| 0 | Vitvi10g04574\_t001 |  |  |  |  |  |  |  |  |  |  |  |  |
| 0 | Vitvi10g04575\_t001 |  |  |  |  |  |  |  |  |  |  |  |  |
| 0 | Vitvi10g01211\_t001 |  |  |  |  |  |  |  |  |  |  |  |  |
| 0 | Vitvi10g01213\_t001 |  |  |  |  |  |  |  |  |  |  |  |  |
| 0 | Vitvi10g01216\_t001 |  |  |  |  |  |  |  |  |  |  |  |  |
| 0 | Vitvi10g01219\_t001 |  |  |  |  |  |  |  |  |  |  |  |  |
| 0 | Vitvi10g01970\_t001 |  |  |  |  |  |  |  |  |  |  |  |  |
| 0 | Vitvi10g01224\_t001 |  |  |  |  |  |  |  |  |  |  |  |  |
| 0 | Vitvi10g01227\_t001 |  |  |  |  |  |  |  |  |  |  |  |  |
| 0 | Vitvi10g01228\_t001 |  |  |  |  |  |  |  |  |  |  |  |  |
| 0 | Vitvi10g04576\_t001 |  |  |  |  |  |  |  |  |  |  |  |  |
| 0 | Vitvi10g01230\_t001 |  |  |  |  |  |  |  |  |  |  |  |  |
| 0 | Vitvi10g01232\_t001 |  |  |  |  |  |  |  |  |  |  |  |  |
| 0 | Vitvi10g01233\_t002 |  |  |  |  |  |  |  |  |  |  |  |  |
| 0 | Vitvi10g01236\_t001 |  |  |  |  |  |  |  |  |  |  |  |  |
| 0 | Vitvi10g01237\_t001 |  |  |  |  |  |  |  |  |  |  |  |  |
| 0 | Vitvi10g01239\_t001 |  |  |  |  |  |  |  |  |  |  |  |  |
| 0 | Vitvi10g01972\_t001 |  |  |  |  |  |  |  |  |  |  |  |  |
| 0 | Vitvi10g01973\_t001 |  |  |  |  |  |  |  |  |  |  |  |  |
| 0 | Vitvi10g01974\_t001 |  |  |  |  |  |  |  |  |  |  |  |  |
| 0 | Vitvi10g01240\_t001 |  |  |  |  |  |  |  |  |  |  |  |  |
| 0 | Vitvi10g01975\_t001 |  |  |  |  |  |  |  |  |  |  |  |  |
| 0 | Vitvi10g01245\_t001 |  |  |  |  |  |  |  |  |  |  |  |  |
| 0 | Vitvi10g01978\_t001 |  |  |  |  |  |  |  |  |  |  |  |  |
| 0 | Vitvi10g01247\_t001 |  |  |  |  |  |  |  |  |  |  |  |  |
| 0 | Vitvi10g01248\_t001 |  |  |  |  |  |  |  |  |  |  |  |  |
| 0 | Vitvi10g04577\_t001 |  |  |  |  |  |  |  |  |  |  |  |  |
| 0 | Vitvi10g04578\_t001 |  |  |  |  |  |  |  |  |  |  |  |  |
| 0 | Vitvi10g04579\_t001 |  |  |  |  |  |  |  |  |  |  |  |  |
| 0 | Vitvi10g04580\_t001 |  |  |  |  |  |  |  |  |  |  |  |  |
| 0 | Vitvi10g04581\_t001 |  |  |  |  |  |  |  |  |  |  |  |  |
| 1 | Vitvi10g01984\_t001 |  | Vitvi03g01765\_t001 |  |  |  |  |  |  |  |  |  |  |
| 1 | Vitvi10g04582\_t001 |  | Vitvi03g04327\_t001 |  |  |  |  |  |  |  |  |  |  |
| 2 | Vitvi10g01254\_t001 |  | Vitvi03g04329\_t001 |  | Vitvi16g04196\_t001 |  |  |  |  |  |  |  |  |
| 2 | Vitvi10g01255\_t001 |  | Vitvi03g04333\_t001 |  | Vitvi16g02172\_t001 |  |  |  |  |  |  |  |  |
| 2 | Vitvi10g01257\_t001 |  | Vitvi03g04335\_t001 |  | Vitvi16g04198\_t001 |  |  |  |  |  |  |  |  |
| 2 | Vitvi10g04583\_t001 |  | \| \| |  | \| \| |  |  |  |  |  |  |  |  |
| 2 | Vitvi10g04584\_t001 |  | Vitvi03g04336\_t001 |  | \| \| |  |  |  |  |  |  |  |  |
| 2 | Vitvi10g04585\_t001 |  | \| \| |  | Vitvi16g04199\_t001 |  |  |  |  |  |  |  |  |
| 2 | Vitvi10g04586\_t001 |  | \| \| |  | \| \| |  |  |  |  |  |  |  |  |
| 2 | Vitvi10g01987\_t001 |  | \| \| |  | Vitvi16g04200\_t001 |  |  |  |  |  |  |  |  |
| 2 | Vitvi10g04587\_t001 |  | \| \| |  | \| \| |  |  |  |  |  |  |  |  |
| 2 | Vitvi10g04588\_t001 |  | Vitvi03g04346\_t001 |  | \| \| |  |  |  |  |  |  |  |  |
| 1 | Vitvi10g04589\_t001 |  |  |  | \| \| |  |  |  |  |  |  |  |  |
| 1 | Vitvi10g04590\_t001 |  |  |  | \| \| |  |  |  |  |  |  |  |  |
| 1 | Vitvi10g04591\_t001 |  |  |  | \| \| |  |  |  |  |  |  |  |  |
| 1 | Vitvi10g04592\_t001 |  |  |  | \| \| |  |  |  |  |  |  |  |  |
| 1 | Vitvi10g01990\_t001 |  |  |  | \| \| |  |  |  |  |  |  |  |  |
| 1 | Vitvi10g04593\_t001 |  |  |  | \| \| |  |  |  |  |  |  |  |  |
| 1 | Vitvi10g04594\_t001 |  |  |  | \| \| |  |  |  |  |  |  |  |  |
| 1 | Vitvi10g04595\_t001 |  |  |  | \| \| |  |  |  |  |  |  |  |  |
| 1 | Vitvi10g04596\_t001 |  |  |  | Vitvi16g04202\_t001 |  |  |  |  |  |  |  |  |
| 1 | Vitvi10g04597\_t001 |  |  |  | Vitvi16g04203\_t001 |  |  |  |  |  |  |  |  |
| 1 | Vitvi10g04598\_t001 |  |  |  | \| \| |  |  |  |  |  |  |  |  |
| 1 | Vitvi10g04599\_t001 |  |  |  | \| \| |  |  |  |  |  |  |  |  |
| 1 | Vitvi10g04600\_t001 |  |  |  | \| \| |  |  |  |  |  |  |  |  |
| 1 | Vitvi10g01289\_t001 |  |  |  | \| \| |  |  |  |  |  |  |  |  |
| 1 | Vitvi10g04601\_t001 |  |  |  | \| \| |  |  |  |  |  |  |  |  |
| 1 | Vitvi10g04602\_t001 |  |  |  | \| \| |  |  |  |  |  |  |  |  |
| 1 | Vitvi10g04603\_t001 |  |  |  | \| \| |  |  |  |  |  |  |  |  |
| 1 | Vitvi10g04604\_t001 |  |  |  | \| \| |  |  |  |  |  |  |  |  |
| 1 | Vitvi10g04605\_t001 |  |  |  | \| \| |  |  |  |  |  |  |  |  |
| 1 | Vitvi10g04606\_t001 |  |  |  | \| \| |  |  |  |  |  |  |  |  |
| 1 | Vitvi10g04607\_t001 |  |  |  | Vitvi16g00561\_t001 |  |  |  |  |  |  |  |  |
| 0 | Vitvi10g04608\_t001 |  |  |  |  |  |  |  |  |  |  |  |  |
| 0 | Vitvi10g01318\_t001 |  |  |  |  |  |  |  |  |  |  |  |  |
| 0 | Vitvi10g01319\_t001 |  |  |  |  |  |  |  |  |  |  |  |  |
| 0 | Vitvi10g01325\_t001 |  |  |  |  |  |  |  |  |  |  |  |  |
| 0 | Vitvi10g04609\_t001 |  |  |  |  |  |  |  |  |  |  |  |  |
| 0 | Vitvi10g04610\_t001 |  |  |  |  |  |  |  |  |  |  |  |  |
| 0 | Vitvi10g01334\_t001 |  |  |  |  |  |  |  |  |  |  |  |  |
| 0 | Vitvi10g01335\_t001 |  |  |  |  |  |  |  |  |  |  |  |  |
| 0 | Vitvi10g01336\_t001 |  |  |  |  |  |  |  |  |  |  |  |  |
| 0 | Vitvi10g01337\_t001 |  |  |  |  |  |  |  |  |  |  |  |  |
| 0 | Vitvi10g01338\_t001 |  |  |  |  |  |  |  |  |  |  |  |  |
| 0 | Vitvi10g04611\_t001 |  |  |  |  |  |  |  |  |  |  |  |  |
| 0 | Vitvi10g01339\_t001 |  |  |  |  |  |  |  |  |  |  |  |  |
| 0 | Vitvi10g01341\_t001 |  |  |  |  |  |  |  |  |  |  |  |  |
| 0 | Vitvi10g04612\_t001 |  |  |  |  |  |  |  |  |  |  |  |  |
| 0 | Vitvi10g01342\_t001 |  |  |  |  |  |  |  |  |  |  |  |  |
| 0 | Vitvi10g04613\_t001 |  |  |  |  |  |  |  |  |  |  |  |  |
| 0 | Vitvi10g01346\_t001 |  |  |  |  |  |  |  |  |  |  |  |  |
| 0 | Vitvi10g04614\_t001 |  |  |  |  |  |  |  |  |  |  |  |  |
| 0 | Vitvi10g01350\_t001 |  |  |  |  |  |  |  |  |  |  |  |  |
| 0 | Vitvi10g04615\_t001 |  |  |  |  |  |  |  |  |  |  |  |  |
| 0 | Vitvi10g01351\_t001 |  |  |  |  |  |  |  |  |  |  |  |  |
| 0 | Vitvi10g04616\_t001 |  |  |  |  |  |  |  |  |  |  |  |  |
| 0 | Vitvi10g01356\_t001 |  |  |  |  |  |  |  |  |  |  |  |  |
| 0 | Vitvi10g01358\_t001 |  |  |  |  |  |  |  |  |  |  |  |  |
| 0 | Vitvi10g01359\_t001 |  |  |  |  |  |  |  |  |  |  |  |  |
| 0 | Vitvi10g01360\_t001 |  |  |  |  |  |  |  |  |  |  |  |  |
| 0 | Vitvi10g01361\_t001 |  |  |  |  |  |  |  |  |  |  |  |  |
| 0 | Vitvi10g02009\_t001 |  |  |  |  |  |  |  |  |  |  |  |  |
| 0 | Vitvi10g04617\_t001 |  |  |  |  |  |  |  |  |  |  |  |  |
| 0 | Vitvi10g01366\_t001 |  |  |  |  |  |  |  |  |  |  |  |  |
| 0 | Vitvi10g04618\_t001 |  |  |  |  |  |  |  |  |  |  |  |  |
| 0 | Vitvi10g04619\_t001 |  |  |  |  |  |  |  |  |  |  |  |  |
| 0 | Vitvi10g04620\_t001 |  |  |  |  |  |  |  |  |  |  |  |  |
| 0 | Vitvi10g04621\_t001 |  |  |  |  |  |  |  |  |  |  |  |  |
| 0 | Vitvi10g04622\_t001 |  |  |  |  |  |  |  |  |  |  |  |  |
| 0 | Vitvi10g01377\_t001 |  |  |  |  |  |  |  |  |  |  |  |  |
| 0 | Vitvi10g01381\_t001 |  |  |  |  |  |  |  |  |  |  |  |  |
| 0 | Vitvi10g01384\_t001 |  |  |  |  |  |  |  |  |  |  |  |  |
| 0 | Vitvi10g01386\_t001 |  |  |  |  |  |  |  |  |  |  |  |  |
| 0 | Vitvi10g01388\_t001 |  |  |  |  |  |  |  |  |  |  |  |  |
| 0 | Vitvi10g04623\_t001 |  |  |  |  |  |  |  |  |  |  |  |  |
| 0 | Vitvi10g04624\_t001 |  |  |  |  |  |  |  |  |  |  |  |  |
| 0 | Vitvi10g04625\_t001 |  |  |  |  |  |  |  |  |  |  |  |  |
| 0 | Vitvi10g04626\_t001 |  |  |  |  |  |  |  |  |  |  |  |  |
| 0 | Vitvi10g04627\_t001 |  |  |  |  |  |  |  |  |  |  |  |  |
| 0 | Vitvi10g01394\_t001 |  |  |  |  |  |  |  |  |  |  |  |  |
| 0 | Vitvi10g01395\_t001 |  |  |  |  |  |  |  |  |  |  |  |  |
| 0 | Vitvi10g01396\_t001 |  |  |  |  |  |  |  |  |  |  |  |  |
| 0 | Vitvi10g04628\_t001 |  |  |  |  |  |  |  |  |  |  |  |  |
| 0 | Vitvi10g04629\_t001 |  |  |  |  |  |  |  |  |  |  |  |  |
| 0 | Vitvi10g02015\_t001 |  |  |  |  |  |  |  |  |  |  |  |  |
| 0 | Vitvi10g04630\_t001 |  |  |  |  |  |  |  |  |  |  |  |  |
| 0 | Vitvi10g02016\_t001 |  |  |  |  |  |  |  |  |  |  |  |  |
| 0 | Vitvi10g04631\_t001 |  |  |  |  |  |  |  |  |  |  |  |  |
| 0 | Vitvi10g04632\_t001 |  |  |  |  |  |  |  |  |  |  |  |  |
| 0 | Vitvi10g04633\_t001 |  |  |  |  |  |  |  |  |  |  |  |  |
| 0 | Vitvi10g04634\_t001 |  |  |  |  |  |  |  |  |  |  |  |  |
| 0 | Vitvi10g04635\_t001 |  |  |  |  |  |  |  |  |  |  |  |  |
| 0 | Vitvi10g04636\_t001 |  |  |  |  |  |  |  |  |  |  |  |  |
| 0 | Vitvi10g01406\_t001 |  |  |  |  |  |  |  |  |  |  |  |  |
| 0 | Vitvi10g04637\_t001 |  |  |  |  |  |  |  |  |  |  |  |  |
| 0 | Vitvi10g01408\_t001 |  |  |  |  |  |  |  |  |  |  |  |  |
| 0 | Vitvi10g04638\_t001 |  |  |  |  |  |  |  |  |  |  |  |  |
| 0 | Vitvi10g01411\_t001 |  |  |  |  |  |  |  |  |  |  |  |  |
| 0 | Vitvi10g04639\_t001 |  |  |  |  |  |  |  |  |  |  |  |  |
| 0 | Vitvi10g04640\_t001 |  |  |  |  |  |  |  |  |  |  |  |  |
| 0 | Vitvi10g04641\_t001 |  |  |  |  |  |  |  |  |  |  |  |  |
| 0 | Vitvi10g04642\_t001 |  |  |  |  |  |  |  |  |  |  |  |  |
| 0 | Vitvi10g04643\_t001 |  |  |  |  |  |  |  |  |  |  |  |  |
| 0 | Vitvi10g04644\_t001 |  |  |  |  |  |  |  |  |  |  |  |  |
| 0 | Vitvi10g04645\_t001 |  |  |  |  |  |  |  |  |  |  |  |  |
| 0 | Vitvi10g02023\_t001 |  |  |  |  |  |  |  |  |  |  |  |  |
| 0 | Vitvi10g02024\_t001 |  |  |  |  |  |  |  |  |  |  |  |  |
| 0 | Vitvi10g04646\_t001 |  |  |  |  |  |  |  |  |  |  |  |  |
| 0 | Vitvi10g01419\_t002 |  |  |  |  |  |  |  |  |  |  |  |  |
| 0 | Vitvi10g01422\_t001 |  |  |  |  |  |  |  |  |  |  |  |  |
| 0 | Vitvi10g01423\_t001 |  |  |  |  |  |  |  |  |  |  |  |  |
| 0 | Vitvi10g01424\_t001 |  |  |  |  |  |  |  |  |  |  |  |  |
| 0 | Vitvi10g04647\_t001 |  |  |  |  |  |  |  |  |  |  |  |  |
| 0 | Vitvi10g04648\_t001 |  |  |  |  |  |  |  |  |  |  |  |  |
| 0 | Vitvi10g04649\_t001 |  |  |  |  |  |  |  |  |  |  |  |  |
| 0 | Vitvi10g04650\_t001 |  |  |  |  |  |  |  |  |  |  |  |  |
| 0 | Vitvi10g04651\_t001 |  |  |  |  |  |  |  |  |  |  |  |  |
| 0 | Vitvi10g01435\_t001 |  |  |  |  |  |  |  |  |  |  |  |  |
| 0 | Vitvi10g04652\_t001 |  |  |  |  |  |  |  |  |  |  |  |  |
| 0 | Vitvi10g04653\_t001 |  |  |  |  |  |  |  |  |  |  |  |  |
| 0 | Vitvi10g02029\_t001 |  |  |  |  |  |  |  |  |  |  |  |  |
| 0 | Vitvi10g04654\_t001 |  |  |  |  |  |  |  |  |  |  |  |  |
| 0 | Vitvi10g04655\_t001 |  |  |  |  |  |  |  |  |  |  |  |  |
| 0 | Vitvi10g04656\_t001 |  |  |  |  |  |  |  |  |  |  |  |  |
| 0 | Vitvi10g01438\_t001 |  |  |  |  |  |  |  |  |  |  |  |  |
| 0 | Vitvi10g04657\_t001 |  |  |  |  |  |  |  |  |  |  |  |  |
| 0 | Vitvi10g04658\_t001 |  |  |  |  |  |  |  |  |  |  |  |  |
| 0 | Vitvi10g04659\_t001 |  |  |  |  |  |  |  |  |  |  |  |  |
| 0 | Vitvi10g04660\_t001 |  |  |  |  |  |  |  |  |  |  |  |  |
| 0 | Vitvi10g04661\_t001 |  |  |  |  |  |  |  |  |  |  |  |  |
| 0 | Vitvi10g04662\_t001 |  |  |  |  |  |  |  |  |  |  |  |  |
| 0 | Vitvi10g04663\_t001 |  |  |  |  |  |  |  |  |  |  |  |  |
| 0 | Vitvi10g04664\_t001 |  |  |  |  |  |  |  |  |  |  |  |  |
| 0 | Vitvi10g04665\_t001 |  |  |  |  |  |  |  |  |  |  |  |  |
| 0 | Vitvi10g04666\_t001 |  |  |  |  |  |  |  |  |  |  |  |  |
| 0 | Vitvi10g04667\_t001 |  |  |  |  |  |  |  |  |  |  |  |  |
| 0 | Vitvi10g04668\_t001 |  |  |  |  |  |  |  |  |  |  |  |  |
| 0 | Vitvi10g01455\_t001 |  |  |  |  |  |  |  |  |  |  |  |  |
| 0 | Vitvi10g01456\_t001 |  |  |  |  |  |  |  |  |  |  |  |  |
| 0 | Vitvi10g04669\_t001 |  |  |  |  |  |  |  |  |  |  |  |  |
| 0 | Vitvi10g01457\_t001 |  |  |  |  |  |  |  |  |  |  |  |  |
| 0 | Vitvi10g01458\_t001 |  |  |  |  |  |  |  |  |  |  |  |  |
| 0 | Vitvi10g04670\_t001 |  |  |  |  |  |  |  |  |  |  |  |  |
| 0 | Vitvi10g04671\_t001 |  |  |  |  |  |  |  |  |  |  |  |  |
| 0 | Vitvi10g04672\_t001 |  |  |  |  |  |  |  |  |  |  |  |  |
| 0 | Vitvi10g01465\_t001 |  |  |  |  |  |  |  |  |  |  |  |  |
| 0 | Vitvi10g01466\_t001 |  |  |  |  |  |  |  |  |  |  |  |  |
| 0 | Vitvi10g04673\_t001 |  |  |  |  |  |  |  |  |  |  |  |  |
| 0 | Vitvi10g01468\_t001 |  |  |  |  |  |  |  |  |  |  |  |  |
| 0 | Vitvi10g04674\_t001 |  |  |  |  |  |  |  |  |  |  |  |  |
| 0 | Vitvi10g04675\_t001 |  |  |  |  |  |  |  |  |  |  |  |  |
| 0 | Vitvi10g01470\_t001 |  |  |  |  |  |  |  |  |  |  |  |  |
| 0 | Vitvi10g01471\_t001 |  |  |  |  |  |  |  |  |  |  |  |  |
| 0 | Vitvi10g04676\_t001 |  |  |  |  |  |  |  |  |  |  |  |  |
| 0 | Vitvi10g04677\_t001 |  |  |  |  |  |  |  |  |  |  |  |  |
| 0 | Vitvi10g01473\_t001 |  |  |  |  |  |  |  |  |  |  |  |  |
| 0 | Vitvi10g01476\_t001 |  |  |  |  |  |  |  |  |  |  |  |  |
| 0 | Vitvi10g04678\_t001 |  |  |  |  |  |  |  |  |  |  |  |  |
| 0 | Vitvi10g01477\_t001 |  |  |  |  |  |  |  |  |  |  |  |  |
| 0 | Vitvi10g01478\_t001 |  |  |  |  |  |  |  |  |  |  |  |  |
| 0 | Vitvi10g01479\_t002 |  |  |  |  |  |  |  |  |  |  |  |  |
| 0 | Vitvi10g02037\_t001 |  |  |  |  |  |  |  |  |  |  |  |  |
| 0 | Vitvi10g02038\_t001 |  |  |  |  |  |  |  |  |  |  |  |  |
| 0 | Vitvi10g04679\_t001 |  |  |  |  |  |  |  |  |  |  |  |  |
| 0 | Vitvi10g04680\_t001 |  |  |  |  |  |  |  |  |  |  |  |  |
| 0 | Vitvi10g01482\_t001 |  |  |  |  |  |  |  |  |  |  |  |  |
| 0 | Vitvi10g02041\_t001 |  |  |  |  |  |  |  |  |  |  |  |  |
| 0 | Vitvi10g04681\_t001 |  |  |  |  |  |  |  |  |  |  |  |  |
| 0 | Vitvi10g04682\_t001 |  |  |  |  |  |  |  |  |  |  |  |  |
| 0 | Vitvi10g04683\_t001 |  |  |  |  |  |  |  |  |  |  |  |  |
| 0 | Vitvi10g01483\_t001 |  |  |  |  |  |  |  |  |  |  |  |  |
| 0 | Vitvi10g04684\_t001 |  |  |  |  |  |  |  |  |  |  |  |  |
| 0 | Vitvi10g04685\_t001 |  |  |  |  |  |  |  |  |  |  |  |  |
| 0 | Vitvi10g02044\_t001 |  |  |  |  |  |  |  |  |  |  |  |  |
| 0 | Vitvi10g02045\_t001 |  |  |  |  |  |  |  |  |  |  |  |  |
| 0 | Vitvi10g04686\_t001 |  |  |  |  |  |  |  |  |  |  |  |  |
| 0 | Vitvi10g04687\_t001 |  |  |  |  |  |  |  |  |  |  |  |  |
| 0 | Vitvi10g04688\_t001 |  |  |  |  |  |  |  |  |  |  |  |  |
| 0 | Vitvi10g04689\_t001 |  |  |  |  |  |  |  |  |  |  |  |  |
| 0 | Vitvi10g01490\_t001 |  |  |  |  |  |  |  |  |  |  |  |  |
| 0 | Vitvi10g01492\_t001 |  |  |  |  |  |  |  |  |  |  |  |  |
| 0 | Vitvi10g04690\_t001 |  |  |  |  |  |  |  |  |  |  |  |  |
| 0 | Vitvi10g04691\_t001 |  |  |  |  |  |  |  |  |  |  |  |  |
| 0 | Vitvi10g02047\_t001 |  |  |  |  |  |  |  |  |  |  |  |  |
| 0 | Vitvi10g04692\_t001 |  |  |  |  |  |  |  |  |  |  |  |  |
| 0 | Vitvi10g04693\_t001 |  |  |  |  |  |  |  |  |  |  |  |  |
| 0 | Vitvi10g02048\_t001 |  |  |  |  |  |  |  |  |  |  |  |  |
| 0 | Vitvi10g04694\_t001 |  |  |  |  |  |  |  |  |  |  |  |  |
| 0 | Vitvi10g02049\_t001 |  |  |  |  |  |  |  |  |  |  |  |  |
| 0 | Vitvi10g04695\_t001 |  |  |  |  |  |  |  |  |  |  |  |  |
| 0 | Vitvi10g01495\_t001 |  |  |  |  |  |  |  |  |  |  |  |  |
| 0 | Vitvi10g01497\_t001 |  |  |  |  |  |  |  |  |  |  |  |  |
| 0 | Vitvi10g01499\_t001 |  |  |  |  |  |  |  |  |  |  |  |  |
| 0 | Vitvi10g04696\_t001 |  |  |  |  |  |  |  |  |  |  |  |  |
| 0 | Vitvi10g04697\_t001 |  |  |  |  |  |  |  |  |  |  |  |  |
| 0 | Vitvi10g04698\_t001 |  |  |  |  |  |  |  |  |  |  |  |  |
| 0 | Vitvi10g01505\_t001 |  |  |  |  |  |  |  |  |  |  |  |  |
| 0 | Vitvi10g02053\_t001 |  |  |  |  |  |  |  |  |  |  |  |  |
| 0 | Vitvi10g01506\_t001 |  |  |  |  |  |  |  |  |  |  |  |  |
| 0 | Vitvi10g04699\_t001 |  |  |  |  |  |  |  |  |  |  |  |  |
| 0 | Vitvi10g01507\_t001 |  |  |  |  |  |  |  |  |  |  |  |  |
| 0 | Vitvi10g01508\_t001 |  |  |  |  |  |  |  |  |  |  |  |  |
| 0 | Vitvi10g04700\_t001 |  |  |  |  |  |  |  |  |  |  |  |  |
| 0 | Vitvi10g04701\_t001 |  |  |  |  |  |  |  |  |  |  |  |  |
| 0 | Vitvi10g04702\_t001 |  |  |  |  |  |  |  |  |  |  |  |  |
| 0 | Vitvi10g04703\_t001 |  |  |  |  |  |  |  |  |  |  |  |  |
| 0 | Vitvi10g04704\_t001 |  |  |  |  |  |  |  |  |  |  |  |  |
| 0 | Vitvi10g04705\_t001 |  |  |  |  |  |  |  |  |  |  |  |  |
| 0 | Vitvi10g01513\_t001 |  |  |  |  |  |  |  |  |  |  |  |  |
| 0 | Vitvi10g04706\_t001 |  |  |  |  |  |  |  |  |  |  |  |  |
| 0 | Vitvi10g04707\_t001 |  |  |  |  |  |  |  |  |  |  |  |  |
| 0 | Vitvi10g04708\_t001 |  |  |  |  |  |  |  |  |  |  |  |  |
| 0 | Vitvi10g02068\_t001 |  |  |  |  |  |  |  |  |  |  |  |  |
| 0 | Vitvi10g04709\_t001 |  |  |  |  |  |  |  |  |  |  |  |  |
| 0 | Vitvi10g01517\_t001 |  |  |  |  |  |  |  |  |  |  |  |  |
| 0 | Vitvi10g01519\_t001 |  |  |  |  |  |  |  |  |  |  |  |  |
| 0 | Vitvi10g01520\_t001 |  |  |  |  |  |  |  |  |  |  |  |  |
| 0 | Vitvi10g04710\_t001 |  |  |  |  |  |  |  |  |  |  |  |  |
| 0 | Vitvi10g04711\_t001 |  |  |  |  |  |  |  |  |  |  |  |  |
| 0 | Vitvi10g04712\_t001 |  |  |  |  |  |  |  |  |  |  |  |  |
| 0 | Vitvi10g04713\_t001 |  |  |  |  |  |  |  |  |  |  |  |  |
| 0 | Vitvi10g02075\_t001 |  |  |  |  |  |  |  |  |  |  |  |  |
| 0 | Vitvi10g01525\_t001 |  |  |  |  |  |  |  |  |  |  |  |  |
| 0 | Vitvi10g01526\_t001 |  |  |  |  |  |  |  |  |  |  |  |  |
| 0 | Vitvi10g04714\_t001 |  |  |  |  |  |  |  |  |  |  |  |  |
| 0 | Vitvi10g01527\_t001 |  |  |  |  |  |  |  |  |  |  |  |  |
| 0 | Vitvi10g04715\_t001 |  |  |  |  |  |  |  |  |  |  |  |  |
| 0 | Vitvi10g04716\_t001 |  |  |  |  |  |  |  |  |  |  |  |  |
| 0 | Vitvi10g04717\_t001 |  |  |  |  |  |  |  |  |  |  |  |  |
| 0 | Vitvi10g04718\_t001 |  |  |  |  |  |  |  |  |  |  |  |  |
| 0 | Vitvi10g01533\_t001 |  |  |  |  |  |  |  |  |  |  |  |  |
| 0 | Vitvi10g01534\_t001 |  |  |  |  |  |  |  |  |  |  |  |  |
| 0 | Vitvi10g01535\_t001 |  |  |  |  |  |  |  |  |  |  |  |  |
| 0 | Vitvi10g01538\_t001 |  |  |  |  |  |  |  |  |  |  |  |  |
| 0 | Vitvi10g01540\_t001 |  |  |  |  |  |  |  |  |  |  |  |  |
| 0 | Vitvi10g04719\_t001 |  |  |  |  |  |  |  |  |  |  |  |  |
| 0 | Vitvi10g01541\_t001 |  |  |  |  |  |  |  |  |  |  |  |  |
| 0 | Vitvi10g01542\_t001 |  |  |  |  |  |  |  |  |  |  |  |  |
| 0 | Vitvi10g01543\_t001 |  |  |  |  |  |  |  |  |  |  |  |  |
| 0 | Vitvi10g01544\_t001 |  |  |  |  |  |  |  |  |  |  |  |  |
| 0 | Vitvi10g01545\_t001 |  |  |  |  |  |  |  |  |  |  |  |  |
| 0 | Vitvi10g04720\_t001 |  |  |  |  |  |  |  |  |  |  |  |  |
| 0 | Vitvi10g04721\_t001 |  |  |  |  |  |  |  |  |  |  |  |  |
| 0 | Vitvi10g01548\_t001 |  |  |  |  |  |  |  |  |  |  |  |  |
| 0 | Vitvi10g01549\_t001 |  |  |  |  |  |  |  |  |  |  |  |  |
| 0 | Vitvi10g01552\_t003 |  |  |  |  |  |  |  |  |  |  |  |  |
| 0 | Vitvi10g01553\_t001 |  |  |  |  |  |  |  |  |  |  |  |  |
| 0 | Vitvi10g04722\_t001 |  |  |  |  |  |  |  |  |  |  |  |  |
| 0 | Vitvi10g04723\_t001 |  |  |  |  |  |  |  |  |  |  |  |  |
| 0 | Vitvi10g04724\_t001 |  |  |  |  |  |  |  |  |  |  |  |  |
| 0 | Vitvi10g01554\_t001 |  |  |  |  |  |  |  |  |  |  |  |  |
| 0 | Vitvi10g01555\_t002 |  |  |  |  |  |  |  |  |  |  |  |  |
| 0 | Vitvi10g02083\_t001 |  |  |  |  |  |  |  |  |  |  |  |  |
| 0 | Vitvi10g01557\_t001 |  |  |  |  |  |  |  |  |  |  |  |  |
| 0 | Vitvi10g01558\_t001 |  |  |  |  |  |  |  |  |  |  |  |  |
| 0 | Vitvi10g04725\_t001 |  |  |  |  |  |  |  |  |  |  |  |  |
| 0 | Vitvi10g04726\_t001 |  |  |  |  |  |  |  |  |  |  |  |  |
| 0 | Vitvi10g04727\_t001 |  |  |  |  |  |  |  |  |  |  |  |  |
| 0 | Vitvi10g01563\_t001 |  |  |  |  |  |  |  |  |  |  |  |  |
| 0 | Vitvi10g01564\_t001 |  |  |  |  |  |  |  |  |  |  |  |  |
| 0 | Vitvi10g01565\_t007 |  |  |  |  |  |  |  |  |  |  |  |  |
| 0 | Vitvi10g01566\_t001 |  |  |  |  |  |  |  |  |  |  |  |  |
| 0 | Vitvi10g04728\_t001 |  |  |  |  |  |  |  |  |  |  |  |  |
| 0 | Vitvi10g01568\_t001 |  |  |  |  |  |  |  |  |  |  |  |  |
| 0 | Vitvi10g04729\_t001 |  |  |  |  |  |  |  |  |  |  |  |  |
| 0 | Vitvi10g04730\_t001 |  |  |  |  |  |  |  |  |  |  |  |  |
| 0 | Vitvi10g01569\_t001 |  |  |  |  |  |  |  |  |  |  |  |  |
| 0 | Vitvi10g01571\_t001 |  |  |  |  |  |  |  |  |  |  |  |  |
| 0 | Vitvi10g01573\_t001 |  |  |  |  |  |  |  |  |  |  |  |  |
| 0 | Vitvi10g02085\_t001 |  |  |  |  |  |  |  |  |  |  |  |  |
| 0 | Vitvi10g02086\_t001 |  |  |  |  |  |  |  |  |  |  |  |  |
| 0 | Vitvi10g01577\_t001 |  |  |  |  |  |  |  |  |  |  |  |  |
| 0 | Vitvi10g01578\_t001 |  |  |  |  |  |  |  |  |  |  |  |  |
| 0 | Vitvi10g01579\_t001 |  |  |  |  |  |  |  |  |  |  |  |  |
| 0 | Vitvi10g01581\_t001 |  |  |  |  |  |  |  |  |  |  |  |  |
| 0 | Vitvi10g04731\_t001 |  |  |  |  |  |  |  |  |  |  |  |  |
| 0 | Vitvi10g01582\_t001 |  |  |  |  |  |  |  |  |  |  |  |  |
| 0 | Vitvi10g04732\_t001 |  |  |  |  |  |  |  |  |  |  |  |  |
| 0 | Vitvi10g01584\_t002 |  |  |  |  |  |  |  |  |  |  |  |  |
| 0 | Vitvi10g04733\_t001 |  |  |  |  |  |  |  |  |  |  |  |  |
| 0 | Vitvi10g01585\_t001 |  |  |  |  |  |  |  |  |  |  |  |  |
| 0 | Vitvi10g04734\_t001 |  |  |  |  |  |  |  |  |  |  |  |  |
| 0 | Vitvi10g04735\_t001 |  |  |  |  |  |  |  |  |  |  |  |  |
| 0 | Vitvi10g04736\_t001 |  |  |  |  |  |  |  |  |  |  |  |  |
| 0 | Vitvi10g01587\_t001 |  |  |  |  |  |  |  |  |  |  |  |  |
| 0 | Vitvi10g04737\_t001 |  |  |  |  |  |  |  |  |  |  |  |  |
| 0 | Vitvi10g04738\_t001 |  |  |  |  |  |  |  |  |  |  |  |  |
| 0 | Vitvi10g04739\_t001 |  |  |  |  |  |  |  |  |  |  |  |  |
